# Impact of multi-drug resistant bacteria on economic and clinical outcomes of healthcare-associated infections in adults: Systematic review and meta-analysis

**DOI:** 10.1371/journal.pone.0227139

**Published:** 2020-01-10

**Authors:** Miquel Serra-Burriel, Matthew Keys, Carlos Campillo-Artero, Antonella Agodi, Martina Barchitta, Achilleas Gikas, Carlos Palos, Guillem López-Casasnovas

**Affiliations:** 1 Center for Research in Health and Economics, Pompeu Fabra University, Barcelona, Spain; 2 Balearic Islands Health Service, Palma de Mallorca, Balearic Islands, Spain; 3 Department of Medical and Surgical Sciences and Advanced Technologies “GF Ingrassia”, University of Catania, Catania, Italy; 4 Internal Medicine Department, Infectious Diseases Unit, University Hospital of Heraklion, Crete, Greece; 5 School of Medicine, University of Crete, Heraklion, Greece; 6 Hospital Beatriz Ângelo, Loures, Lisbon, Portugal; University of Mississippi Medical Center, UNITED STATES

## Abstract

**Background:**

Infections with multidrug resistant (MDR) bacteria in hospital settings have substantial implications in terms of clinical and economic outcomes. However, due to clinical and methodological heterogeneity, estimates about the attributable economic and clinical effects of healthcare-associated infections (HAI) due to MDR microorganisms (MDR HAI) remain unclear. The objective was to review and synthesize the evidence on the impact of MDR HAI in adults on hospital costs, length of stay, and mortality at discharge.

**Methods and findings:**

Literature searches were conducted in PubMed/MEDLINE, and Google Scholar databases to select studies that evaluated the impact of MDR HAI on economic and clinical outcomes. Eligible studies were conducted in adults, in order to ensure homogeneity of populations, used propensity score matched cohorts or included explicit confounding control, and had confirmed antibiotic susceptibility testing. Risk of bias was evaluated, and effects were measured with ratios of means (ROM) for cost and length of stay, and risk ratios (RR) for mortality. A systematic search was performed on 14^th^ March 2019, re-run on the 10^th^ of June 2019 and extended the 3^rd^ of September 2019. Small effect sizes were assessed by examination of funnel plots. Sixteen articles (6,122 patients with MDR HAI and 8,326 patients with non-MDR HAI) were included in the systematic review of which 12 articles assessed cost, 19 articles length of stay, and 14 mortality. Compared to susceptible infections, MDR HAI were associated with increased cost (ROM 1.33, 95%CI [1.15; 1.54]), prolonged length of stay (ROM 1.27, 95%CI [1.18; 1.37]), and excess in-hospital mortality (RR 1.61, 95%CI [1.36; 1.90]) in the random effects models. Risk of publication bias was only found to be significant for mortality, and overall study quality good.

**Conclusions:**

MDR HAI appears to be strongly associated with increases in direct cost, prolonged length of stay and increased mortality. However, further comprehensive studies in this setting are warranted.

**Trial registration:**

PROSPERO (CRD42019126288).

## Introduction

The growing prevalence of bacterial infections that cannot be adequately treated by existing effective antimicrobial therapies poses a considerable threat to the effectiveness, efficiency of healthcare systems[1–6], and is currently reaching societal consequences[7–9] limiting the achievement of WHO’s Sustainable Development Goals[10,11].

Much work has been done on understanding and estimating the impact of antimicrobial resistance (AMR) in various settings. A recent article[12] underlines that AMR is one of the greatest challenges for public health and highlights the high impact of healthcare-associated infections (HAIs) due to antimicrobial resistant bacteria in terms of number of cases, attributable deaths, and disability-adjusted life-years. 700.000 deaths due to infections caused by Multi-drug resistant (MDR) bacteria occur yearly worldwide and this number could increase to 10 million in 2050[13] depending upon resistance patterns evolution and effective antibiotic discovery. Estimated associated costs are estimated to be 3.8% of annual GDP (additional 1.2 trillion USD) and as a results, 28.3 million people are condemned to extreme poverty. “*Three out of four deaths from superbug infections could be averted by spending just USD 2 per person a year on measures as simple as hand washing and more prudent prescription of antibiotics*”[14].

With respect to AMR, the most commonly reported characterization is multidrug resistance (MDR)[15–19], which occurs when a bacterial infection is resistant to treatment with multiple appropriate antimicrobial drugs. MDR is currently characterized as non-susceptibility to at least one agent in three antimicrobial categories [20]. Associations between resistant infections and a wide arrange of outcomes has been reported and their direct effects are mainly driven by the ineffectiveness of available treatment of such infections. However, the relative magnitude of effects remains unclear. Moreover, estimates of the iatrogenic burden of MDR in hospital nosocomial infections could have been biased due to small sample sizes studies, inadequate research methodology and measurement error.

The objective of this study was to systematically review the literature and to provide a quantitative synthesis of the available evidence regarding the adjusted association between HAI due to MDR bacteria and direct cost of care, extended length of stay and mortality in observational studies with the highest degree of internal validity.

## Materials and methods

This systematic review and meta-analysis adheres to the PRISMA recommendations[21]. Furthermore, specific recommendations for systematic review and metanalysis of observational studies were followed[22]. This metanalysis was registered with PROSPERO (CRD42019126288).

### Study eligibility criteria

Studies met eligibility criteria if they were case-control or cohort studies conducted in adults evaluating the effects of MDR HAI vs non-MDR HAI. We excluded non-adjusted studies due to the excess risk of bias within the observational setting of this analysis. Two consecutive rounds of eligibility criteria were applied to ensure comparability across studies.

First, each included study must: i) have been published between 1st January 1980 - 3rd September 2019 in English, ii) attempt to estimate the direct impact of MDR (MDR was defined in accordance to the current available interim expert proposal[20]) HAIs on either clinical or economic outcomes using original data with a case-control or cohort study design, and, iii) attempt to control for confounding either through matching[23], inclusion of sensible covariates in a multivariate analysis, or both[24].

A second round of eligibility criteria was applied upon examination of the full text of each study for inclusion in the systematic review. In particular, each study must: i) have a control group that differs in the antibiotic susceptibility pattern of the microorganism causing infections (confirmed by susceptibility testing), ii) present comprehensive descriptive statistics (gender composition, age, microorganism distribution) of the case and control groups with estimated variance parameters of the dependent variables, iii) examine at least one of the following outcomes: mortality at discharge, direct hospital cost/charges or length of stay, and, iv) in case of inclusion of community-acquired infection (CAI) cases, results had to be reported separately for CAI and HAI cases while fulfilling the previous inclusion conditions.

### Identification and selection of studies

A systematic search was performed on 14^th^ March 2019,re-run on the 10^th^ of June 2019 and extended the 3^rd^ of September 2019. We searched PubMed/MEDLINE and Google Scholar databases for eligible studies. Two authors (MSB and MK) performed the title screening to find retrospective or prospective studies reporting an adjusted association of MDR HAI with mortality, length of stay and/or direct cost of care. A specific-search strategy was tailored to each database by using MeSH/Map terms and is presented as supplementary S3 Table. The degree of agreement across the two reviewers for inclusion assessment was assessed using kappa statistics.

**Fig 1:**
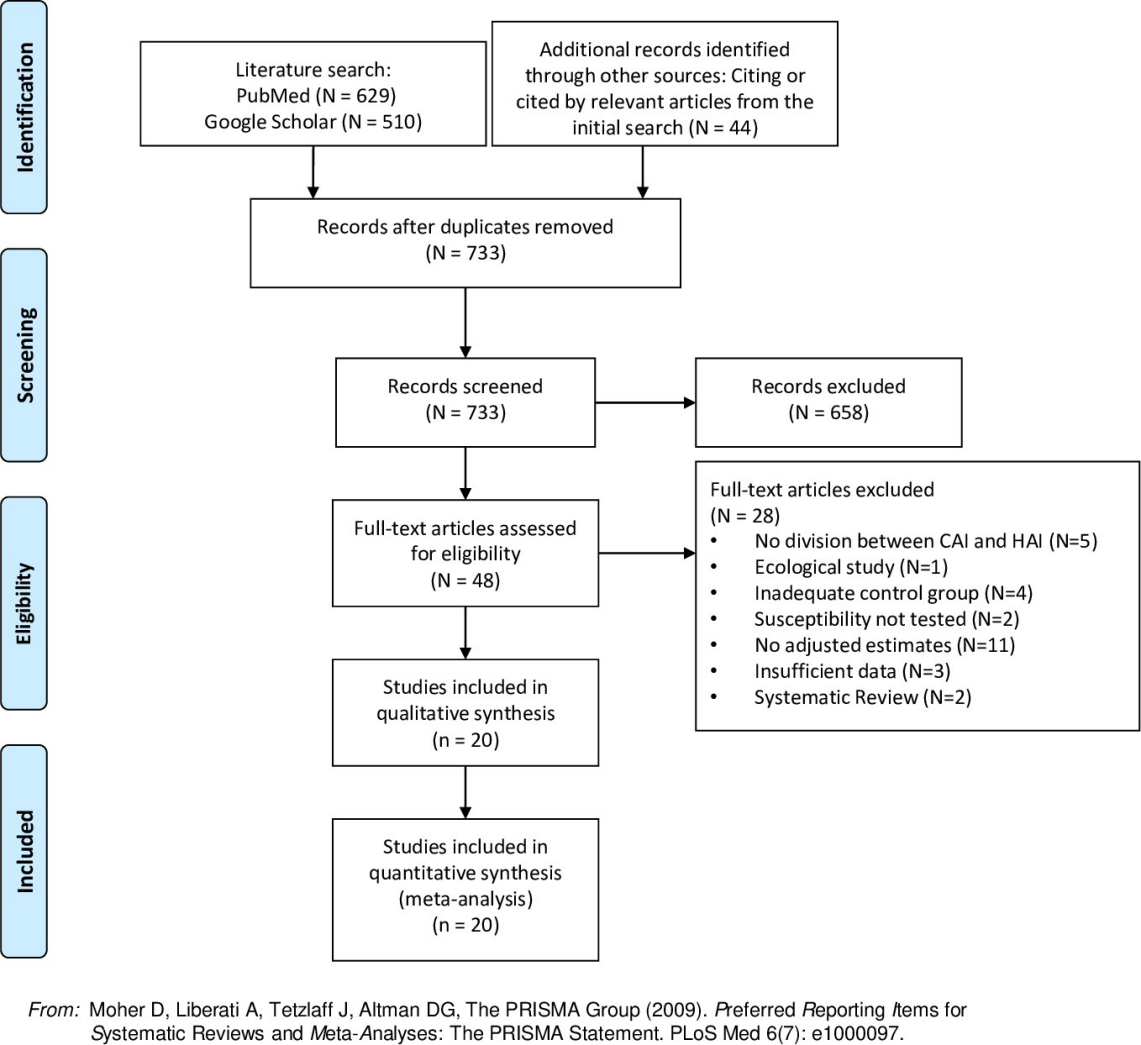
PRISMA flow diagram.

Additional papers were also collected through expert consultation, and references of individual papers and previous meta-analyses. The search flow chart is presented in Fig 1.

### Data extraction and risk of bias assessment

Two predefined tables were used to summarize information from the final eligible studies. In the first one, for each paper, we recorded the basic results about the estimated impact of MDR HAI on each clinical and economic outcome (adjusted difference, outcomes mean, and, sample sizes). In the second one, we recorded the descriptive statistics provided on the study samples used for the final analysis. Specific Items were extracted for each study: clinical setting, study design (prospective or retrospective), origin of infection (hospital and/or community), infection type. These data extraction procedures were performed by two authors, and disagreement was resolved by discussion with a third author (CC). Three primary outcomes were evaluated: i) direct hospitalization cost or hospital charges, ii) length of stay, and iii) mortality at discharge.

Direct hospitalization cost was defined as the total direct cost from the hospital perspective of the inpatient admission, when unavailable, hospital charges were used as a proxy. Length of stay was defined as the admission length of stay following infection up to discharge. Mortality was defined as patient death at the time of discharge.

Various methodologies are regularly employed to control for confounding and to approximate unbiased, causal estimates. In order to assess the risk of bias per study we used the Newcastle-Ottawa Scale designed for assessing the quality of non-randomized studies in a meta-analysis[25–27]. Publication bias was assessed with funnel plots.

### Data synthesis

All statistical analyses were performed in R, version 3.5.1 (2018-07-02)[28] with the meta package[29]. Difference in outcomes was expressed as ratio of means (ROM) for cost and length of stay outcomes, and risk ratio (RR) for mortality at discharge. The rationale for expressing outcomes as ROM, was to eliminate between-studies variability between the reporting of direct hospital cost or charges, both random-effects (RE) and fixed-effects (FE) models were fitted for statistical pooling of the data. Given the clinical heterogeneity present in the analysis, a random effects model with the Hartung-Knapp-Sidik-Jonkman method was chosen as the primary estimation method[30]. The weighting of the results was performed in relation to the sample size of each study. Given the low likelihood of obtaining 10 or more studies by covariate, meta-regression was not performed[31].

Statistical heterogeneity for each pooled outcome was examined with the I^2^ statistic[32] and Q-test[33]. Significance level was established at the 5% level, and all results are expressed with 95% confidence intervals (CI). Prediction interval was also estimated in the pooling[34]. Risk of publication bias was visually examined through funnel plots and assessed with a linear regression test of funnel plot asymmetry[35].

## Results

Fig 1 depicts a flow diagram of the search, following PRISMA guidelines, screening and eligibility procedures and the loss of studies that occurred throughout. Table 1 describes the studies that were included after the second round of eligibility criteria applied to the full-text studies. Out of 733 selected studies, 658 (93.5%) were excluded after screening of the abstract for the first round of inclusion criteria (kappa = 0.73). A total of 48 full-text manuscripts were assessed for the second round of inclusion criteria, with 28 (58.3%) being excluded for not fulfilling the criteria (kappa = 0.96). A total of 20 studies were included in the review, with 14,448 patients included[36–55]. Out of the 20 studies, 19 presented in hospital length of stay, 14 presented mortality data and 12 cost outcomes.

**Table 1:** Summary characteristics of selected studies.

Study ID	Authors	Year	Clinical Setting	Study Design	Origin of Infection	Infection Types	Country of study	Mean Age	Sample size (*N*)
1	R. K. Pelz et al.	2002	Single	Prospective	H	Any	US	56	34
2	L. F. Barat et al.	2017	Single	Prospective	ICU	PN	Spain	66	64
3	B. J. Kopp et al.	2004	Single	Retrospective	H/C	Any	US	57	72
4	R. Tedja et al.	2014	Single	Retrospective	H	PN	US	62	107
5	P. O. Depuydt	2008	Multicenter	Retrospective	H/C	Any	Belgium	59	192
6	I. M. Loeches et al.	2014	Multicenter	Prospective	ICU	PN	Spain	62	171
7	P. D. Mauldin et al.	2010	Single	Retrospective	H	Any	US	N/A*	662
8	J. J. Engemann et al.	2003	Single	Prospective	H	SSI	US^Ω^	59	286
9	E. E. Magira et al.	2017	Single	Retrospective	ICU	Any	US	72	300
10	Y. Carmeli et al.	1999	Single	Retrospective	H/C	Any	US^Ω^	63	489
11	M. Riu et al.	2016	Single	Retrospective	H	BSI	Spain	66	575
12	R. R. Roberts et al.	2009	Single	Retrospective	H/C	Any	US	54	338
13	S.T. Micek et al.	2015	Multicenter	Retrospective	H	PN	EU	56	740
14	Z. Chen et al.	2018	Single	Retrospective	H	PN	China	70	540
15	A. Resch et al.	2009	Multicenter	Retrospective	H/C	Any (MRSA)	Germany	68	2,052
16	M. J. Neidell et al.	2012	Multicenter	Retrospective	H/C	BSI, UTI, PN	US^Ω^	64	1,775
17	L. Puchter et al.	2018	Single	Retrospective	H/ICU	Any	Germany	54	84
18	R. Nelson et al.	2018	Multicenter	Retrospective	H	Any	US VA	70	405
19	E. Cowie et al.	2005	Single	Retrospective	H	SSI	Canada	68	37
20	Bonnet et al.	2019	Multicenter	Retrospective	ICU	Any	France	61	5,525

Abbreviations: H: Hospital, C: community, ICU: Intensive Care Unit, SSI: Surgical Site Infection, BSI: Bloodstream Infection, MRSA: Methicillin-Resistant Staphylococcus Aureus, UTI: Urinary Tract Infection, PN: Pneumonia.

*Study presents discretized age.

^Ω^Hospital charges instead of cost reported.

Most studies were single center (k = 13). Four studies were prospective cohort studies, while the rest (k = 16) were retrospective. Only four studies were conducted in the intensive care units (ICU), while the remaining studies were conducted in other wards, with some including both community-acquired infections and HAI reported separately (k = 6). In terms of type of infection, most studies included any HAI (k = 11), while the rest, pneumonia (k = 6), bloodstream infections (BSI, k = 2), surgical site infections (SSI, k = 2), and urinary tract infections (UTI, k = 1).

### Risk of bias

Risk of bias was rated according to the Newcastle-Ottawa scale and is summarized in S1 Table. The majority of studies were rated as good quality according to the AHQR[26], (k = 9). Six studies were classified as fair and five of poor quality. Case definition was adequately portrayed in most studies (k = 14), selection of controls (k = 17), definition of controls (k = 12), and representativeness of the sample (k = 11). Comparability of cases and controls on the was rated at least with one point (*) in all studies (k = 20). Ascertainment of exposure was adequate in almost all studies (k = 18). Overall, the risk of bias in included studies was found to be low.

### Effects of MDR-HAI

#### Cost

**Fig 2:**
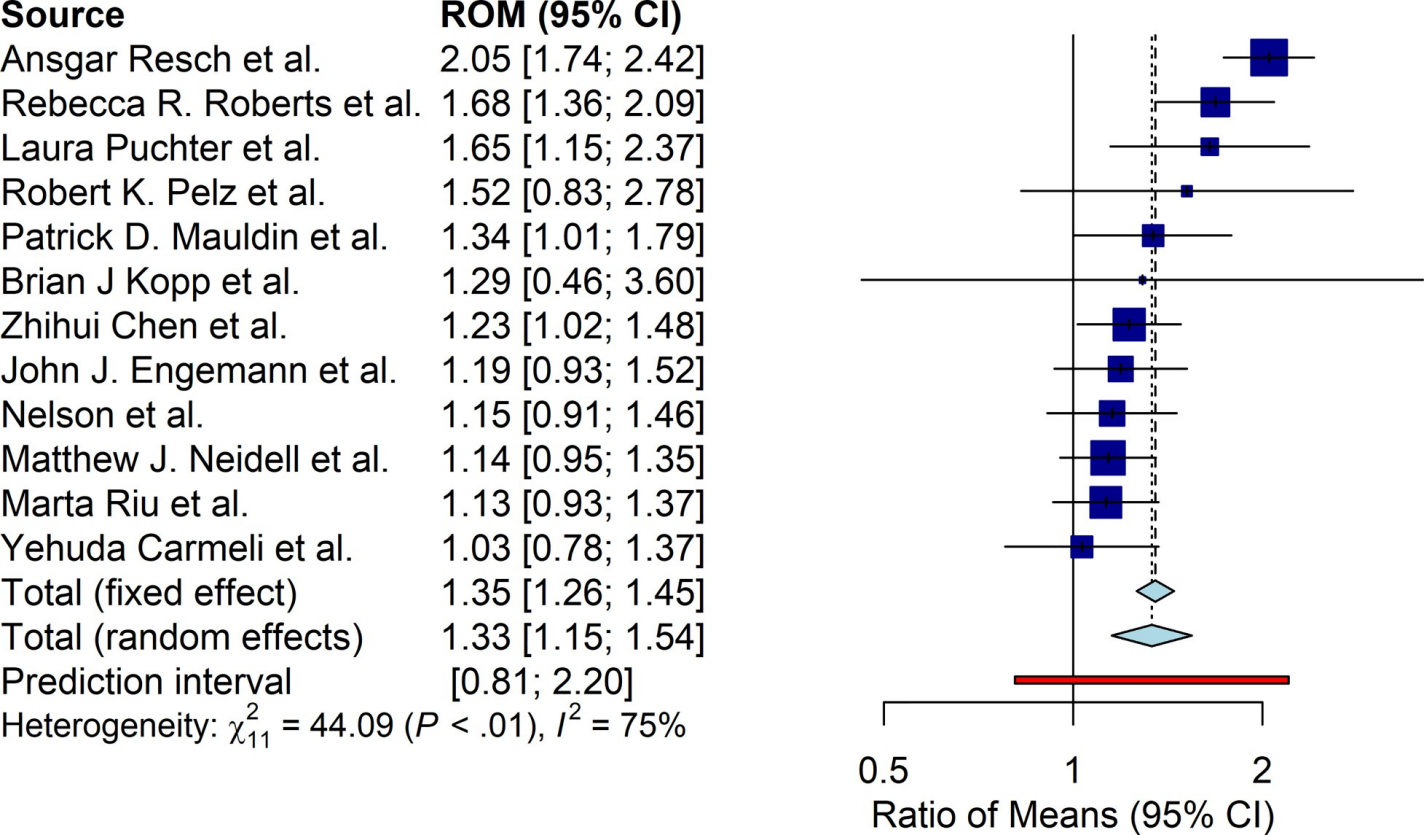
Cost of care ratio of means (ROM) estimates.

Excess attributable cost of MDR HAI was found to be statistically significant in the RE pooling (ROM 1.33 95%CI [1.15; 1.54]). Fig 2 presents the results of the pooling. FE pooling displayed similar results (ROM 1.35 95%CI [1.26; 1.44]), and the prediction interval ranged from 0.81 to 2.20. Significant heterogeneity across studies was found to be present with a Q-statistic value of 44.09 (p<0.01) and an I^2^ statistic of 75%. There was no evidence of publication bias in the 11 studies that reported cost outcomes with the linear regression test of funnel plot asymmetry (p = 0.7715).

#### Length of stay

**Fig 3:**
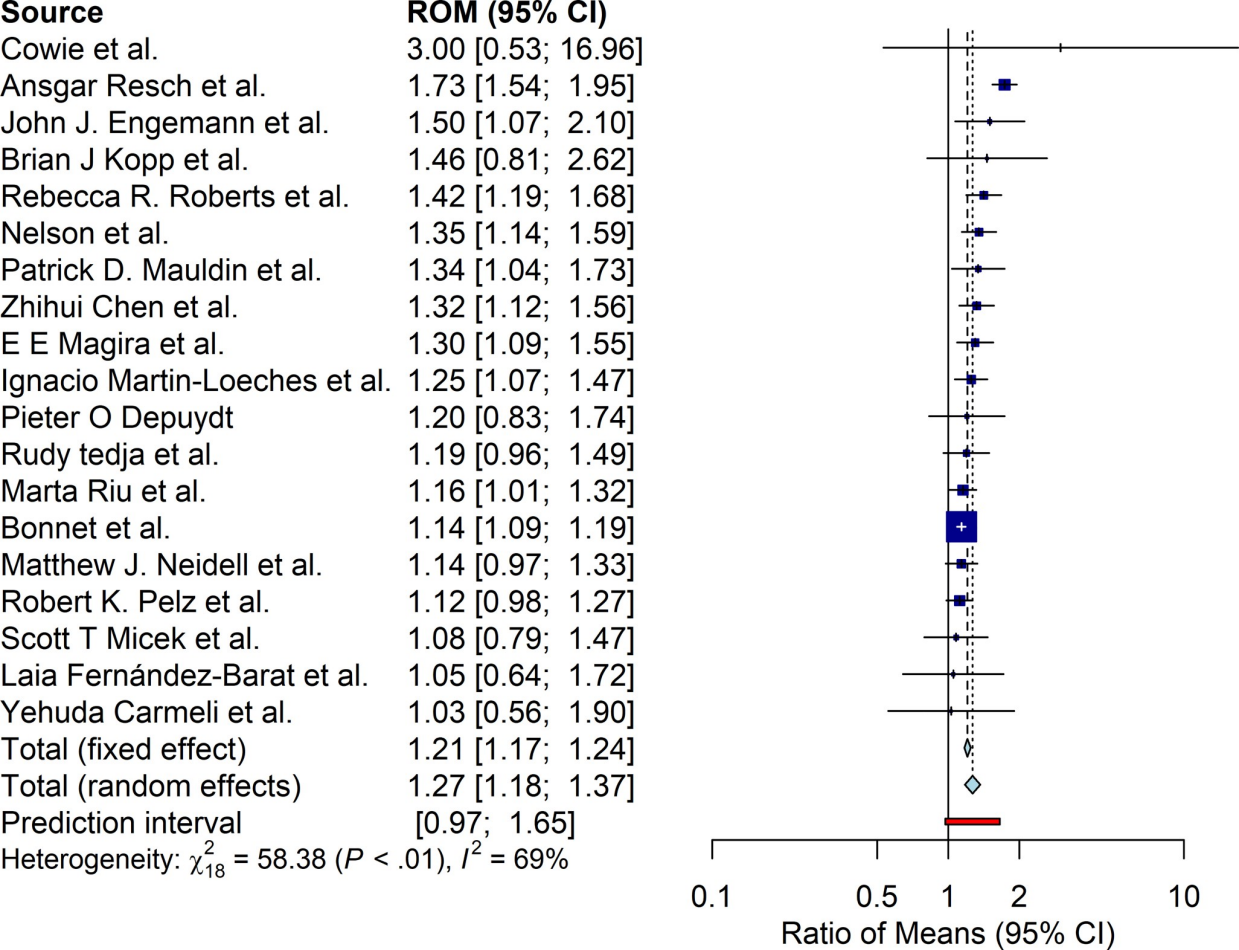
Length of stay ratio of means (ROM) estimates.

Prolonged length of stay of MDR HAI was found to be also significant in the RE pooling (ROM 1.27 95%CI [1.17; 1.39]). FE model yielded similar results (ROM 1.21 [1.17; 1.24]). The prediction interval was found to be narrower than for cost with values ranging from 0.97 to 1.65. Fig 3 presents the results. Heterogeneity was also found to be present, Q-statistic value of 59.38 (p<0.01) and I^2^ value of 69%. We were not able to reject the null hypothesis of symmetry in the funnel plot (p = 0.1258).

#### Mortality at discharge

**Fig 4:**
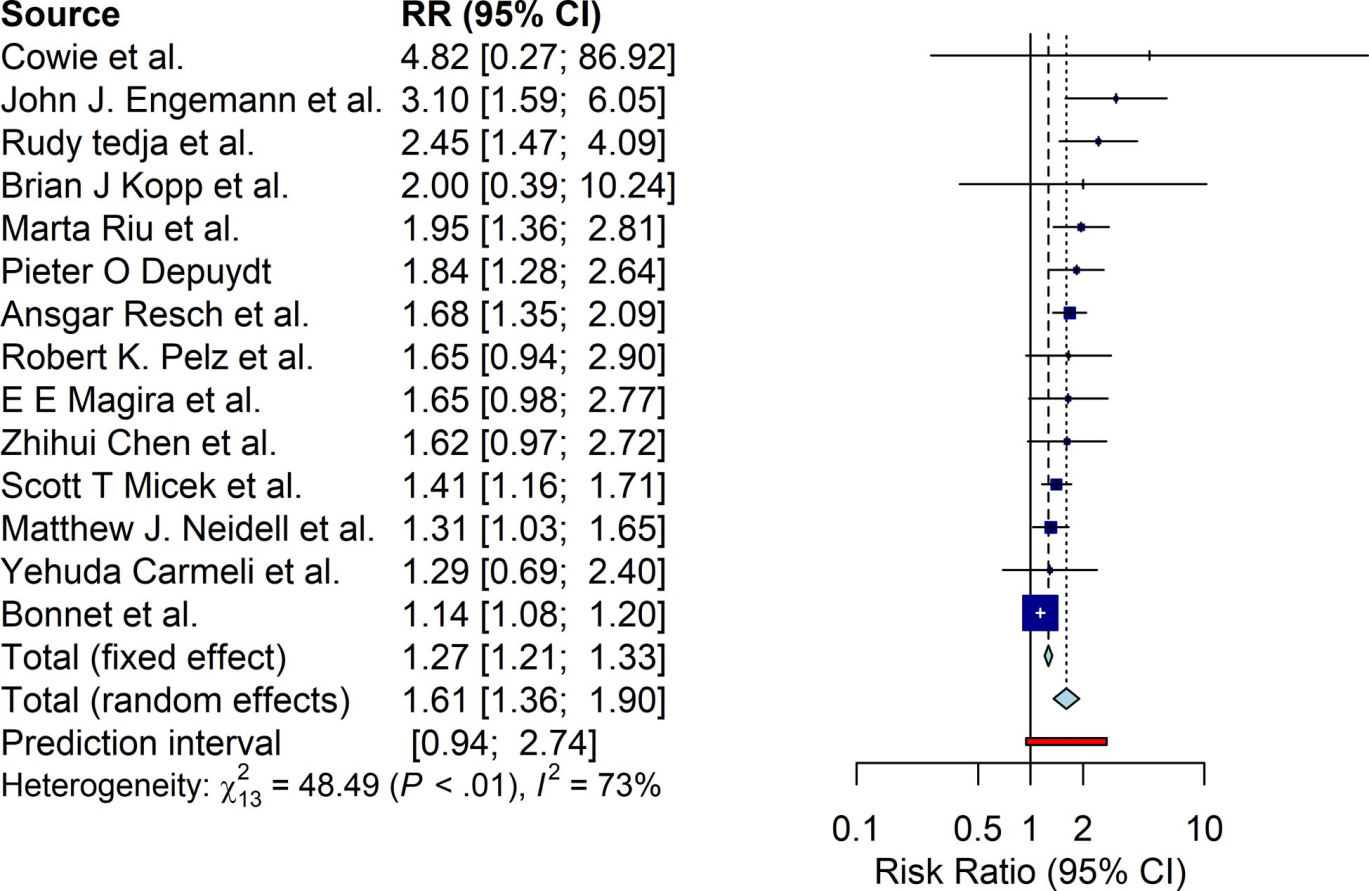
Mortality at discharge relative risk estimates.

Excess mortality results from the 14 eligible studies are displayed in Fig 4. RE pooling shows a significant increase (RR 1.61 95%CI [1.36; 1.90]). FE pooled estimates were much lower than RE (RR 1.27 95%CI [1.21; 1.33]), while the prediction interval ranges from 0.94 to 2.74. Significant heterogeneity across studies was found, Q-statistic 48.49 (p<0.01) and I^2^ statistic of 73%.

**Fig 5:**
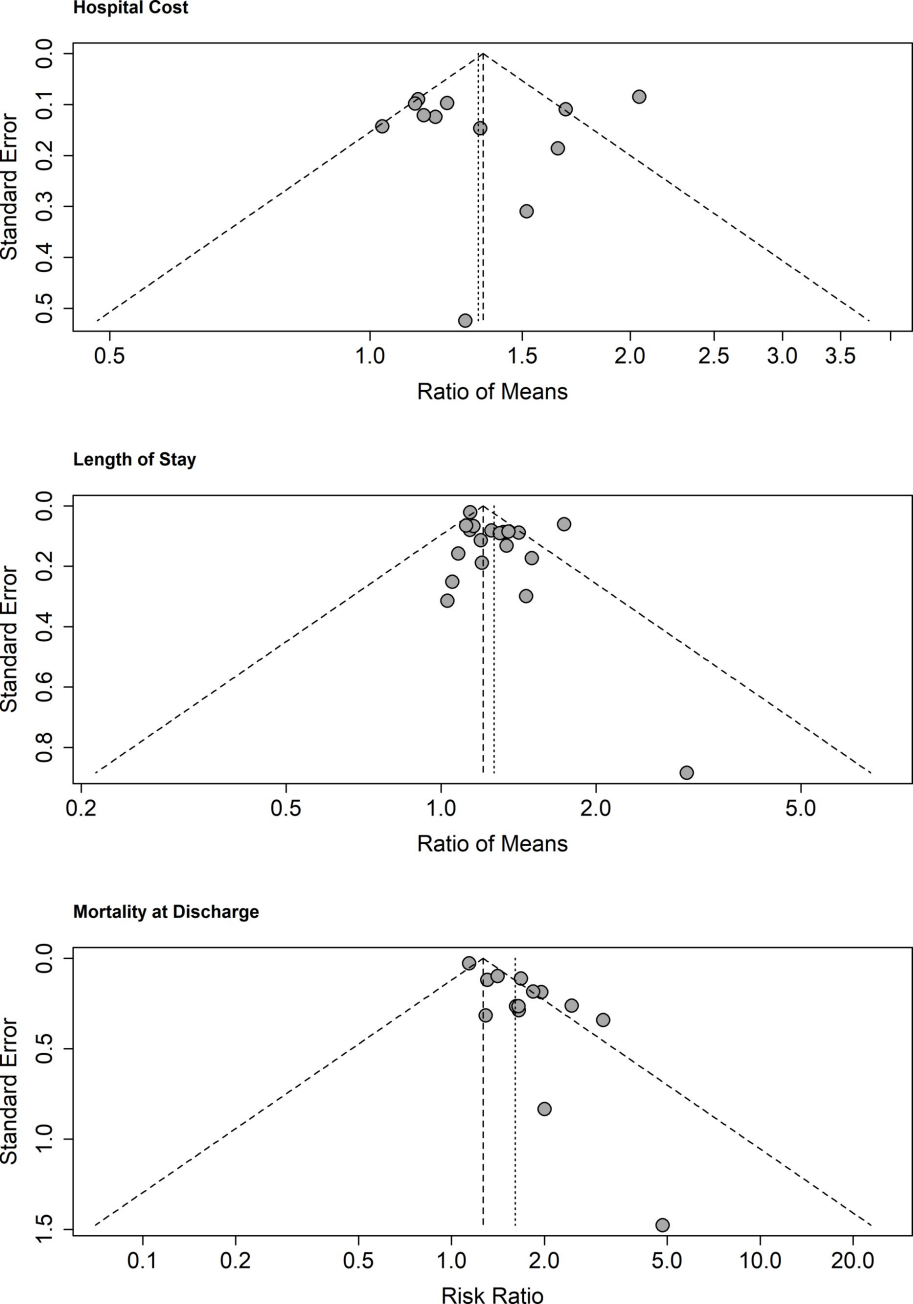
Funnel plots.

Fig 5 displays the funnel plots of all three outcomes. Symmetry was confirmed for cost and length of stay outcomes, while for mortality a significant asymmetry towards increased RR was noted (p<0.01), highlighting a high risk of publication bias in this particular outcome.

## Discussion

HAIs can occur in almost every step of hospital care and are highly heterogeneous in their cause and clinical presentation. It follows that a prominent feature of the literature on the economic and clinical impact of MDR is a large degree of heterogeneity across study settings. Here we reviewed and synthesized the available evidence presented with the highest observational methodological standards due to well understood confounding problems[56]. We limited the analysis to cohort and case-control studies in order to alleviate selection bias, excluding a significant portion of studies, and yielding a small number of studies. Overall, the quality of studies was good, we did, however, found indication of publication bias in mortality estimates.

We found consistent patterns that indicate increased mortality and resource utilization in terms of length of stay and direct cost which are associated with MDR HAI vs those associated with susceptible microorganism acquired infections. We report 1.27, 1.33 and 1.62-fold increases in excess length of stay, cost and mortality at discharge risk associated with MDR respectively. The effect size of length and cost does not vary significantly depending upon the estimation method. However, for mortality, there is a two-fold difference between RE (RR 1.62) and FE (RR 1.27). This difference highlights the potential unobserved heterogeneity across studies in terms of patient prognosis and publication bias. The interrelation of these three outcomes plays a crucial role in the interpretation of the results. Higher inpatient mortality could, theoretically, even reduce inpatient stay and direct hospital cost. However, our estimates point to an overall increase around 30% for resource utilization.

As is the case with observational studies where the exposure is not randomized, there is risk of bias in estimating the impact of exposure. Only 4 out of 20 eligible studies presented a prospective cohort study design that could mitigate selection bias in the estimates. Remaining studies presented retrospective designs and attempted to overcome other sources of bias through a process matching and/or multivariate modelling with appropriate selection of control variables.

The range of costs presented varies considerably according to the setting in which MDR HAIs were studied, ranging from average increases of 3,000 USD to 40,000 USD[36,37]. The reported mechanisms of cost impact also vary across settings. We tried to address this between-studies heterogeneity by using a ROM metric as outcome, rendering interpretable estimates regardless of institutional and national setting[57]. The most consistent mechanism across studies refers to secondary antibiotic treatment, followed by prolonged length of stay and increased labor costs. Furthermore, the MDR cost impact has an average incremental estimate of a 1.33-fold magnitude, highlighting the relative magnitude of the problem.

Further well-designed studies, that use either a consistent control, matching or time-to-infection control, like Nelson et al. (2018) are needed in order to best approximate unbiased and consistent estimates. Their clinical and economic implications are crucial for both the development of novel antibiotics alongside their potential cost-effectiveness estimates.

### Limitations

There are several limitations to this systematic review. There is a general lack of comparability across studies examining the impact of MDR HAI on clinical and economic outcomes, namely in setting, MDR bacteria and infections studied, data presentation, and methodology. Given the potential lack of exogenous variation in the allocation of resistant bacterial infections, confounding may be a problem, which would require elaborate and uniform covariate choice across participating studies so that a comprehensive set of covariates could be used in a multivariate pooled analysis. No restrictions regarding time to HAI were applied for its definition. However, most studies used a 48H window since admission.

Furthermore, there are subtle differences in the definitions of primary endpoints utilized across the eligible studies and MDR definitions, which makes comparison less reliable. For example, one may formulate cost from either the patient or hospital perspective, which may be an estimate of cost rather than the true figure, which poses measurement error. Lastly, a complex grouping of studies was chosen for this meta-analysis. We considered all bacteria types presenting with resistant and many types of infections. Comparisons between these different settings are made more difficult by the inconsistencies across studies. We were also unable to perform reliable subgroup analyses due to the small sample size of eligible studies in each predefined category.

**Fig 6:**
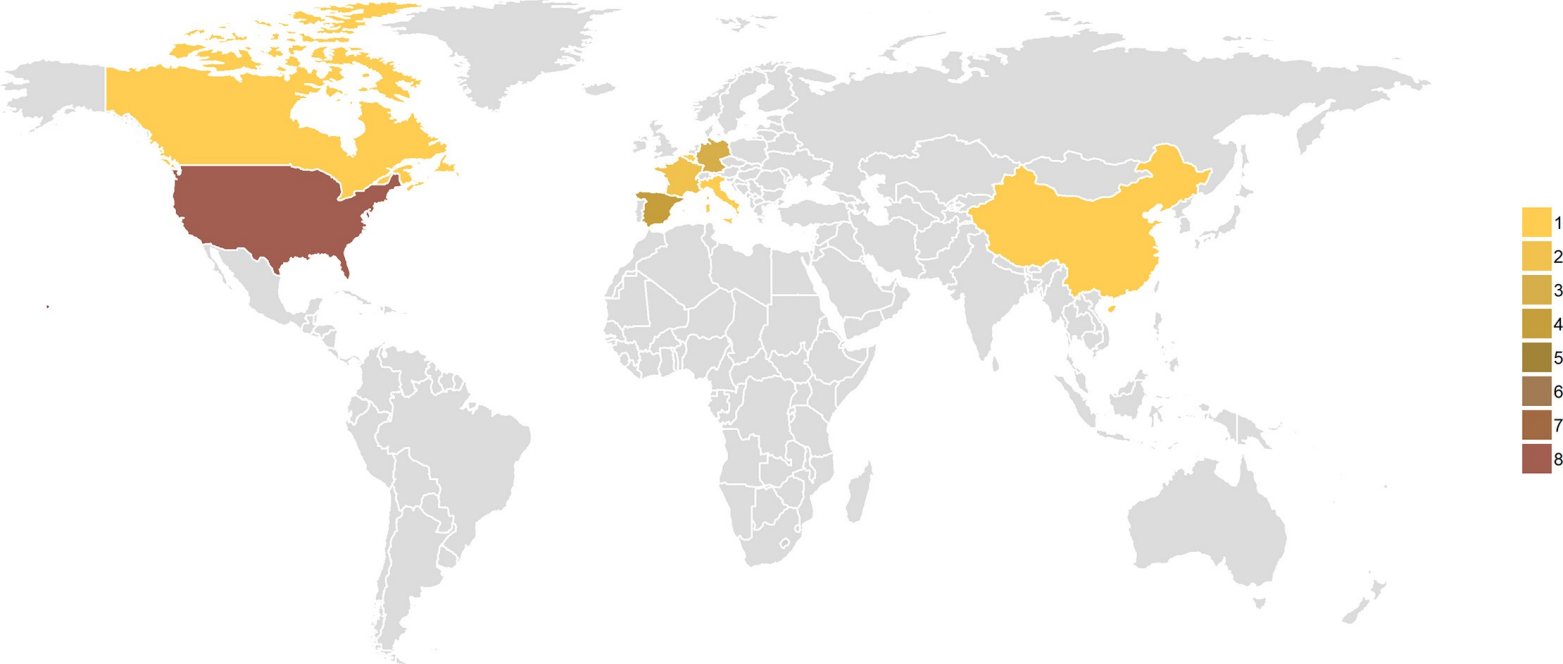
Geographical distribution of included studies.

Finally, our search strategy did not include studies published in non-English languages, potentially yielding under representation of geographic areas where AMR presents an even higher burden. Fig 6 presents the geographical distribution of included studies, mainly in North America and Europe. Further research estimating the pooled burden in non-English speaking countries is warranted in order to provide accurate estimates of the magnitude of the burden.

### Conclusions

MDR HAI appears to be strongly associated with increased resource utilization and health outcomes, as assessed by direct provider cost and/or charges, prolonged length of stay and mortality of patients in a wide array of healthcare settings. However, more research is required to fully elucidate the relationship between MDR and economic and clinical outcomes and its dependence on various settings, routes of infection and pathogens involved. Further applied research should focus on the development of prospective registries with standardized susceptibility testing and longer follow-up outside of hospital settings to better understand the actual societal burden of such a rising phenomenon.

## Supporting information

S1 Checklist PRISMA checklist. (DOC)

S1 Code (R)

S1 Data (XLSX)

S1 Results (DOCX)

S1 Table Summary of Newcastle-Ottawa risk of bias judgements for each study. (DOCX)

S2 Table Quantitative summary of included studies. (DOCX)

S3 Table Search strategy by database. (DOCX)

## Abbreviations

MDR: multidrug resistance
HAI: hospital acquired infection
ICU: intensive care unit
ECDC: European Center for Disease Prevention and Control
CAI: community acquired infection
HAI: healthcare acquired infection
SSI: surgical site infections
CL-BSI: central line bloodstream infections
CAUTIs: catheter associated urinary tract infection
VAP: ventilator associated pneumonia
WHO: World Health Organization
XDR: extensively drug resistance
PDR: pan drug resistance
