# Supplementary material for: Impact of multi-drug resistant bacteria on economic and clinical outcomes of healthcare-associated infections in adults: Systematic review and meta-analysis
Source: PLoS One. 2020 Jan 10;15(1):e0227139. doi: 10.1371/journal.pone.0227139 (PMC6953842; doi:10.1371/journal.pone.0227139)
Supplement: S1 Results — (DOCX) [file pone.0227139.s004.docx]

**Appendix S2.** Full list of screened abstracts

1. Westwood JC, Legace S, Mitchell MA. Hospital-acquired infection: present and future impact and need for positive action. Can Med Assoc J. Canada; 1974;110: 769–774. [0,0]

2. Prevention and control of herpesvirus diseases. Part 1. Clinical and laboratory diagnosis and chemotherapy. A WHO meeting. Bull World Health Organ. Switzerland; 1985;63: 185-201 contd. [0,0]

3. Onesko KM, Wienke EC. The analysis of the impact of a mild, low-iodine, lotion soap on the reduction of nosocomial methicillin-resistant Staphylococcus aureus: a new opportunity for surveillance by objectives. Infect Control. United States; 1987;8: 284–288. [0,0]

4. Wakefield DS, Helms CM, Massanari RM, Mori M, Pfaller M. Cost of nosocomial infection: relative contributions of laboratory, antibiotic, and per diem costs in serious Staphylococcus aureus infections. Am J Infect Control. United States; 1988;16: 185–192. doi:10.1016/0196-6553(88)90058-2[0,0]

5. Goldmann DA. The bacterial flora of neonates in intensive care-monitoring and manipulation. J Hosp Infect. England; 1988;11 Suppl A: 340–351. doi:10.1016/0195-6701(88)90209-5 [0,0]

6. Nakagomi O, Tobita M, Hirasawa H, Kobayashi N, Uesugi S, Takahashi M. [Nosocomial infections and clinical microbiology]. Rinsho Byori. Japan; 1989;37: 1231–1237. [0,0]

7. Harle A. [The importance of nosocomial infections for patients and socio-political representatives]. Z Orthop Ihre Grenzgeb. Germany; 1989;127: 463–466. doi:10.1055/s-2008-1044703 [0,0]

8. Gallo IA, Petrosillo N, Celletti S, Tersigni I, Antonelli L, Maddaluno R, et al. [Microbiological control of hospital infections]. Ann Ig. Italy; 1989;1: 761–767. [0,0]

9. Carton JA, Gomez Moro MB, Gonzalez Lopez B, Maradona JA, de Diego I, Carcaba V, et al. [Nosocomially acquired infection of the urinary tract]. Enferm Infecc Microbiol Clin. Spain; 1989;7: 408–414. [0,0]

10. Meers PD, Leong KY. The impact of methicillin- and aminoglycoside-resistant Staphylococcus aureus on the pattern of hospital-acquired infection in an acute hospital. J Hosp Infect. England; 1990;16: 231–239. doi:10.1016/0195-6701(90)90111-z [0,0]

11. Holzheimer RG, Quoika P, Patzmann D, Fussle R. Nosocomial infections in general surgery: surveillance report from a German university clinic. Infection. Germany; 1990;18: 219–225. doi:10.1007/bf01643391 [0,0]

12. Velasco ED, Martins CA, Vidal E, Carvalho AD, Gaglianone TC. [Hospital infections at an oncology hospital]. Rev Paul Med. Brazil; 1990;108: 61–70. [0,0]

13. Grosserode MH, Wenzel RP. The continuing importance of staphylococci as major hospital pathogens. J Hosp Infect. England; 1991;19 Suppl B: 3–17. doi:10.1016/0195-6701(91)90197-g [0,0]

14. Iwai S, Sato T, Kunimatsu M, Tanaka H, Kato K, Akutsu M, et al. [Hospital acquired infection in surgical field and its countermeasure present situation of anaerobes, P. aeruginosa and MRSA infection]. Nihon Geka Gakkai Zasshi. Japan; 1992;93: 906–909. [0,0]

15. Rossello J, Olona M, Campins M, del Valle O, Bermejo B, Armadans L, et al. Investigation of an outbreak of nosocomial infection due to a multiply drug-resistant strain of Pseudomonas aeruginosa. J Hosp Infect. England; 1992;20: 87–96. doi:10.1016/0195-6701(92)90110-8 [0,0]

16. Winter R, Humphreys H, Pick A, MacGowan AP, Willatts SM, Speller DC. A controlled trial of selective decontamination of the digestive tract in intensive care and its effect on nosocomial infection. J Antimicrob Chemother. England; 1992;30: 73–87. doi:10.1093/jac/30.1.73 [0,0]

17. Gomez EC, Markowsky SJ, Rotschafer JC. Selective decontamination of the digestive tract in intensive care patients: review and commentary. Ann Pharmacother. United States; 1992;26: 963–976. doi:10.1177/106002809202600721 [0,0]

18. Tetteroo GW, Wagenvoort JH, Bruining HA. Role of selective decontamination in surgery. Br J Surg. England; 1992;79: 300–304. doi:10.1002/bjs.1800790405 [0,0]

19. Carlier Y, Rivera MT, Truyens C, Ontivero M, Flament J, Van Marck E, et al. Chagas’ disease: decreased resistance to Trypanosoma cruzi acquired infection in offspring of infected mice. Am J Trop Med Hyg. United States; 1992;46: 116–122. doi:10.4269/ajtmh.1992.46.116 [0,0]

20. Occhipinti DJ, Itokazu G, Danziger LH. Selective decontamination of the digestive tract as an infection-control measure in intensive care unit patients. Pharmacotherapy. United States; 1992;12: 50S-63S. [0,0]

21. Carton JA, Maradona JA, Asensi V, Perez Gonzalez F, Lopez Ponga B, de la Iglesia P, et al. [Hospital infection caused by enterococci. The previous use of antibiotics as risk factors in a case-control study]. Med Clin (Barc). Spain; 1993;101: 769–773. [0,0]

22. Regnier B. [Resources for the campaign against nosocomial infection in hospitals]. Bull Acad Natl Med. Netherlands; 1993;177: 728–729.

23. Cockerill FR 3rd. Indications for selective decontamination of the digestive tract. Semin Respir Infect. United States; 1993;8: 300–307.

24. Monteiro JA. [Nosocomial infections. Some aspects]. Acta Med Port. Portugal; 1993;6: 135–140.

25. Quinio B, Albanese J, Durbec O, Martin C. [Selective digestive decontamination in patients under reanimation]. Ann Fr Anesth Reanim. France; 1994;13: 826–838. doi:10.1016/s0750-7658(05)80920-7

26. Vincent JL, Bihari DJ, Suter PM, Bruining HA, White J, Nicolas-Chanoin MH, et al. The prevalence of nosocomial infection in intensive care units in Europe. Results of the European Prevalence of Infection in Intensive Care (EPIC) Study. EPIC International Advisory Committee. JAMA. United States; 1995;274: 639–644.

27. Gould D, Chamberlaine A. Staphylococcus aureus: a review of the literature. J Clin Nurs. England; 1995;4: 5–12.

28. Potgieter PD, Hammond JM. Prophylactic use of the new quinolones for prevention of nosocomial infection in the intensive care unit. Drugs. New Zealand; 1995;49 Suppl 2: 86–91. doi:10.2165/00003495-199500492-00014

29. Wiener J, Itokazu G, Nathan C, Kabins SA, Weinstein RA. A randomized, double-blind, placebo-controlled trial of selective digestive decontamination in a medical-surgical intensive care unit. Clin Infect Dis. United States; 1995;20: 861–867. doi:10.1093/clinids/20.4.861

30. Torda AJ, Gottlieb T, Bradbury R. Pyogenic vertebral osteomyelitis: analysis of 20 cases and review. Clin Infect Dis. United States; 1995;20: 320–328. doi:10.1093/clinids/20.2.320

31. Barton AL, Doern G V. Selective media for detecting gastrointestinal carriage of vancomycin-resistant enterococci. Diagn Microbiol Infect Dis. United States; 1995;23: 119–122. doi:10.1016/0732-8893(95)00174-3

32. N’Doye B, Hugard L, Dieme Y, Candito D. [Nosocomial septicemias due to multiresistant enterobacteria: preliminary considerations in 32 cases observed in the African hospital environment]. Med Trop (Mars). France; 1995;55: 354–356.

33. Papanicolaou GA, Meyers BR, Meyers J, Mendelson MH, Lou W, Emre S, et al. Nosocomial infections with vancomycin-resistant Enterococcus faecium in liver transplant recipients: risk factors for acquisition and mortality. Clin Infect Dis. United States; 1996;23: 760–766. doi:10.1093/clinids/23.4.760

34. DeRiso AJ 2nd, Ladowski JS, Dillon TA, Justice JW, Peterson AC. Chlorhexidine gluconate 0.12% oral rinse reduces the incidence of total nosocomial respiratory infection and nonprophylactic systemic antibiotic use in patients undergoing heart surgery. Chest. United States; 1996;109: 1556–1561. doi:10.1378/chest.109.6.1556

35. Newman J. The resurgence of TB and its implications for radiology. Radiol Technol. United States; 1996;67: 379–400.

36. Vossing M, Eckel HE, Schlesinger-Raab A, Guntinas-Lichius O, Jansen B. [Nosocomial infections in head and neck surgery. 2. A prospective study]. HNO. Germany; 1996;44: 85–88.

37. Stroud L, Edwards J, Danzing L, Culver D, Gaynes R. Risk factors for mortality associated with enterococcal bloodstream infections. Infect Control Hosp Epidemiol. United States; 1996;17: 576–580. doi:10.1086/647386

38. Yinnon AM, Butnaru A, Raveh D, Jerassy Z, Rudensky B. Klebsiella bacteraemia: community versus nosocomial infection. QJM. England; 1996;89: 933–941. doi:10.1093/qjmed/89.12.933

39. Brucker G. [Status of public health in hospitals in France]. Zentralbl Hyg Umweltmed. Germany; 1996;199: 156–167.

40. Bhutta ZA. Enterobacter sepsis in the newborn--a growing problem in Karachi. J Hosp Infect. England; 1996;34: 211–216. doi:10.1016/s0195-6701(96)90068-7

41. Salama OE. A study of nosocomial infection in a general hospital. J Egypt Public Health Assoc. England; 1996;71: 31–46.

42. Mahon BE, Mintz ED, Greene KD, Wells JG, Tauxe R V. Reported cholera in the United States, 1992-1994: a reflection of global changes in cholera epidemiology. JAMA. United States; 1996;276: 307–312.

43. Holton D, Paton S, Gibson H, Taylor G, Whyman C, Yang T. Comparison of tuberculosis infection control programs in Canadian hospitals categorized by size and risk of exposure to tuberculosis patients, 1989 to 1993 - Part 2. Can J Infect Dis. Canada; 1997;8: 195–201. doi:10.1155/1997/138348

44. Fridkin SK, Welbel SF, Weinstein RA. Magnitude and prevention of nosocomial infections in the intensive care unit. Infect Dis Clin North Am. United States; 1997;11: 479–496.

45. Sopena N, Sabria M, Pedro-Botet ML, Gimenez M, Esteve M, Caraballo M, et al. [Impact of control measures on the course of an outbreak of methicillin-resistant Staphylococcus aureus]. Med Clin (Barc). Spain; 1997;108: 401–404.

46. Opravil M. Epidemiological and clinical aspects of mycobacterial infections. Infection. Germany; 1997;25: 56–59. doi:10.1007/bf02113516

47. Pawa AK, Ramji S, Prakash K, Thirupuram S. Neonatal nosocomial infection: profile and risk factors. Indian Pediatr. India; 1997;34: 297–302.

48. Verwaest C, Verhaegen J, Ferdinande P, Schetz M, Van den Berghe G, Verbist L, et al. Randomized, controlled trial of selective digestive decontamination in 600 mechanically ventilated patients in a multidisciplinary intensive care unit. Crit Care Med. United States; 1997;25: 63–71. doi:10.1097/00003246-199701000-00014

49. de la Cal MA, Cerda E. [Surveillance and control of infections in the intensive care unit: rates, resistance, and carrier state]. Enferm Infecc Microbiol Clin. Spain; 1997;15 Suppl 3: 47–52.

50. Sebille V, Chevret S, Valleron AJ. Modeling the spread of resistant nosocomial pathogens in an intensive-care unit. Infect Control Hosp Epidemiol. United States; 1997;18: 84–92. doi:10.1086/647560

51. Cosseron-Zerbib M, Roque Afonso AM, Naas T, Durand P, Meyer L, Costa Y, et al. A control programme for MRSA (methicillin-resistant Staphylococcus aureus) containment in a paediatric intensive care unit: evaluation and impact on infections caused by other micro-organisms. J Hosp Infect. England; 1998;40: 225–235. doi:10.1016/s0195-6701(98)90140-2

52. Climo MW, Israel DS, Wong ES, Williams D, Coudron P, Markowitz SM. Hospital-wide restriction of clindamycin: effect on the incidence of Clostridium difficile-associated diarrhea and cost. Ann Intern Med. United States; 1998;128: 989–995. doi:10.7326/0003-4819-128-12_part_1-199806150-00005

53. Dieckhaus KD, Cooper BW. Infection control concepts in critical care. Crit Care Clin. United States; 1998;14: 55–70.

54. Kim WJ, Park SC. Bacterial resistance to antimicrobial agents: an overview from Korea. Yonsei Med J. Korea (South); 1998;39: 488–494. doi:10.3349/ymj.1998.39.6.488

55. Ng SP, Gomez JM, Lim SH, Ho NK. Reduction of nosocomial infection in a neonatal intensive care unit (NICU). Singapore Med J. Singapore; 1998;39: 319–323.

56. Martone WJ. Spread of vancomycin-resistant enterococci: why did it happen in the United States? Infect Control Hosp Epidemiol. United States; 1998;19: 539–545. doi:10.1086/647870

57. Cook N. Methicillin-resistant Staphylococcus aureus versus the burn patient. Burns. Netherlands; 1998;24: 91–98. doi:10.1016/s0305-4179(97)00114-9

58. Tokunaga Y, Nakayama N, Nishitai R, Hata K, Kaganoi J, Ohsumi K. Effects of closed-system drain in surgery: focus on methicillin-resistant Staphylococcus aureus. Dig Surg. Switzerland; 1998;15: 352–356. doi:10.1159/000018631

59. Perl TM, Golub JE. New approaches to reduce Staphylococcus aureus nosocomial infection rates: treating S. aureus nasal carriage. Ann Pharmacother. United States; 1998;32: S7-16. doi:10.1177/106002809803200104

60. O’Kane GM, Gottlieb T, Bradbury R. Staphylococcal bacteraemia: the hospital or the home? A review of Staphylococcus aureus bacteraemia at Concord Hospital in 1993. Aust N Z J Med. Australia; 1998;28: 23–27. doi:10.1111/j.1445-5994. 1998.tb04454.x

61. Villari P, Iacuzio L, Torre I, Scarcella A. Molecular epidemiology as an effective tool in the surveillance of infections in the neonatal intensive care unit. J Infect. England; 1998;37: 274–281. doi:10.1016/s0163-4453(98)92107-7

62. Ramsay G, van Saene RH. Selective gut decontamination in intensive care and surgical practice: where are we? World J Surg. United States; 1998;22: 164–170. doi:10.1007/s002689900365

63. Weingarten CM, Rybak MJ, Jahns BE, Stevenson JG, Brown WJ, Levine DP. Evaluation of Acinetobacter baumannii infection and colonization, and antimicrobial treatment patterns in an urban teaching hospital. Pharmacotherapy. United States; 1999;19: 1080–1085. doi:10.1592/phco.19.13.1080.31597

64. Nikodemski T. [Bacteriologic and clinical analysis of nosocomial infections in patients from the intensive care unit]. Ann Acad Med Stetin. Poland; 1999;45: 211–226.

65. Murphy OM, Gould FK. Prevention of nosocomial infection in solid organ transplantation. J Hosp Infect. England; 1999;42: 177–183. doi:10.1053/jhin.1999.0599

66. Rangel-Frausto MS, Morales-Garcia D, Baez-Martinez R, Ibarra-Blancas J, Ponce de Leon-Rosales S. [Evaluation of a nosocomial infection surveillance program]. Salud Publica Mex. Mexico; 1999;41 Suppl 1: S59-63.

67. Dancer SJ. Mopping up hospital infection. J Hosp Infect. England; 1999;43: 85–100. doi:10.1053/jhin.1999.0616

68. Kahla-Clemenceau N, Barre E, Prat H, Thibault M, Bourret C, Richardin F, et al. [Outbreak of methicillin-resistant Staphylococcus aureus in general hospital intensive care unit]. Pathol Biol (Paris). France; 1999;47: 449–456.

69. Kollef MH. Epidemiology and risk factors for nosocomial pneumonia. Emphasis on prevention. Clin Chest Med. United States; 1999;20: 653–670.

70. Jaqua-Stewart MJ, Tjaden J, Humphreys DW, Bade P, Tille PM, Peterson KG, et al. Reduction in methicillin-resistant Staphylococcus aureus infection rate in a nursing home by aggressive containment strategies. S D J Med. United States; 1999;52: 241–247.

71. Pittet D, Hugonnet S, Harbarth S, Mourouga P, Sauvan V, Touveneau S, et al. Effectiveness of a hospital-wide programme to improve compliance with hand hygiene. Infection Control Programme. Lancet (London, England). England; 2000;356: 1307–1312. doi:10.1016/s0140-6736(00)02814-2

72. Vincent JL. Microbial resistance: lessons from the EPIC study. European Prevalence of Infection. Intensive Care Med. United States; 2000;26 Suppl 1: S3-8. doi:10.1007/s001340051111

73. Akbar DH, Mushtaq MA, El-Tahawi AT, Bahnasy AA. Staphylococcus aureus bacteremia. Saudi Med J. Saudi Arabia; 2000;21: 171–174.

74. Rampling A, Wiseman S, Davis L, Hyett AP, Walbridge AN, Payne GC, et al. Evidence that hospital hygiene is important in the control of methicillin-resistant Staphylococcus aureus. J Hosp Infect. England; 2001;49: 109–116. doi:10.1053/jhin.2001.1013

75. Martinez-Aguilar G, Alpuche-Aranda CM, Anaya C, Alcantar-Curiel D, Gayosso C, Daza C, et al. Outbreak of nosocomial sepsis and pneumonia in a newborn intensive care unit by multiresistant extended-spectrum beta-lactamase-producing Klebsiella pneumoniae: high impact on mortality. Infect Control Hosp Epidemiol. United States; 2001;22: 725–728. doi:10.1086/501855

76. Capdevila O, Pallares R, Grau I, Tubau F, Linares J, Ariza J, et al. Pneumococcal peritonitis in adult patients: report of 64 cases with special reference to emergence of antibiotic resistance. Arch Intern Med. United States; 2001;161: 1742–1748. doi:10.1001/archinte.161.14.1742

77. Jones RN. Resistance patterns among nosocomial pathogens: trends over the past few years. Chest. United States; 2001;119: 397S-404S. doi: 10.1378/chest.119.2_suppl.397s

78. Frohloff G. Why is transmission of vancomycin-resistant enterococci on the increase? Prog Transplant. United States; 2001;11: 17–22.

79. Rubinovitch B, Pittet D. Screening for methicillin-resistant Staphylococcus aureus in the endemic hospital: what have we learned? J Hosp Infect. England; 2001;47: 9–18. doi:10.1053/jhin.2000.0873

80. Takezawa J. [Surveillance system for nosocomial infections due to drug-resistant organisms in Japan and other countries]. Nihon Rinsho. Japan; 2001;59: 652–659.

81. Curran E. Reducing the risk of healthcare-acquired infection. Nurs Stand. England; 2001;16: 45. doi:10.7748/ns2001.09.16.1.45.c3086

82. Cisterna R, Cabezas V, Gomez E, Busto C, Atutxa I, Ezpeleta C. [Community-acquired bacteremia]. Rev Esp Quimioter. Spain; 2001;14: 369–382.

83. Tohme A, Karam-Sarkis D, El-Rassi R, Chelala D, Ghayad E. [Agents and consequences of nosocomial infections in a Lebanese university hospital. Retrospective study over a two-year period]. Ann Med Interne (Paris). France; 2001;152: 77–83.

84. Girouard S, Levine G, Goodrich K, Jones S, Keyserling H, Rathore M, et al. Pediatric Prevention Network: a multicenter collaboration to improve health care outcomes. Am J Infect Control. United States; 2001;29: 158–161. doi:10.1067/mic.2001.115405

85. Akbar DH. Urinary tract infection. Diabetics and non-diabetic patients. Saudi Med J. Saudi Arabia; 2001;22: 326–329.

86. Mussi-Pinhata MM, Nascimento SD. [Neonatal nosocomial infections]. J Pediatr (Rio J). Brazil; 2001;77 Suppl 1: S81-96.

87. Schierholz JM, Beuth J, Rump A, Konig DP, Pulverer G. Novel strategies to prevent catheter-associated infections in oncology patients. J Chemother. England; 2001;13 Spec No 1: 239–250. doi: 10.1179/joc.2001.13.Supplement-2.239

88. Allegranzi B, Luzzati R, Luzzani A, Girardini F, Antozzi L, Raiteri R, et al. Impact of antibiotic changes in empirical therapy on antimicrobial resistance in intensive care unit-acquired infections. J Hosp Infect. England; 2002;52: 136–140. doi:10.1053/jhin.2002.1277

89. Sattler CA, Mason EOJ, Kaplan SL. Prospective comparison of risk factors and demographic and clinical characteristics of community-acquired, methicillin-resistant versus methicillin-susceptible Staphylococcus aureus infection in children. Pediatr Infect Dis J. United States; 2002;21: 910–917. doi:10.1097/00006454-200210000-00005

90. Nyamogoba H, Obala AA. Nosocomial infections in developing countries: cost effective control and prevention. East Afr Med J. Kenya; 2002;79: 435–441. doi:10.4314/eamj.v79i8.8831

91. Oncul O, Yuksel F, Altunay H, Acikel C, Celikoz B, Cavuslu S. The evaluation of nosocomial infection during 1-year-period in the burn unit of a training hospital in Istanbul, Turkey. Burns. Netherlands; 2002;28: 738–744. doi:10.1016/s0305-4179(02)00106-7

92. Toltzis P, Dul MJ, Hoyen C, Salvator A, Walsh M, Zetts L, et al. The effect of antibiotic rotation on colonization with antibiotic-resistant bacilli in a neonatal intensive care unit. Pediatrics. United States; 2002;110: 707–711. doi:10.1542/peds.110.4.707

93. Bilal NE, Gedebou M, Al-Ghamdi S. Endemic nosocomial infections and misuse of antibiotics in a maternity hospital in Saudi Arabia. APMIS. Denmark; 2002;110: 140–147. doi:10.1034/j.1600-0463.2002.110204.x

94. Salas Coronas J, Cabezas Fernandez T, Alvarez-Ossorio Garcia de Soria R, Rogado Gonzalez MC, Delgado Fernandez M, Diez Garcia F. [Nosocomial infection/colonization of the respiratory tract caused by Acinetobacter baumannii in an Internal Medicine ward]. An Med Interna. Spain; 2002;19: 511–514.

95. Brown SM, Benneyan JC, Theobald DA, Sands K, Hahn MT, Potter-Bynoe GA, et al. Binary cumulative sums and moving averages in nosocomial infection cluster detection. Emerg Infect Dis. United States; 2002;8: 1426–1432. doi:10.3201/eid0812.010514

96. Iinuma Y. [Acinetobacter]. Nihon Rinsho. Japan; 2002;60: 2161–2165.

97. Velazco E, Nieves B, Araque M, Calderas Z. [Epidemiology of Staphylococcus aureus nosocomial infections in a high-risk neonatal unit]. Enferm Infecc Microbiol Clin. Spain; 2002;20: 321–325. doi:10.1016/s0213-005x(02)72808-5

98. Nambiar S, Singh N. Change in epidemiology of health care-associated infections in a neonatal intensive care unit. Pediatr Infect Dis J. United States; 2002;21: 839–842. doi:10.1097/00006454-200209000-00011

99. Wolff M. [Changes in the epidemiology of infectious diseases in Chile from 1990 to 2000]. Rev Med Chil. Chile; 2002;130: 353–362.

100. Parra Moreno ML, Arias Rivera S, de la Cal Lopez MA, Frutos Vivar F, Cerda Cerda E, Garcia Hierro P, et al. [Effect of selective digestive decontamination on the nosocomial infection and multiresistant microorganisms incidence in critically ill patients]. Med Clin (Barc). Spain; 2002;118: 361–364.

101. Zoutman DE, Ford BD, Bryce E, Gourdeau M, Hebert G, Henderson E, et al. The state of infection surveillance and control in Canadian acute care hospitals. Am J Infect Control. United States; 2003;31: 263–266. doi:10.1067/mic.2003.88

102. Dantas SRPE, Moretti-Branchini ML. Impact of antibiotic-resistant pathogens colonizing the respiratory secretions of patients in an extended-care area of the emergency department. Infect Control Hosp Epidemiol. United States; 2003;24: 351–355. doi:10.1086/502210

103. Evans HL, Sawyer RG. Cycling chemotherapy: a promising approach to reducing the morbidity and mortality of nosocomial infections. Drugs Today (Barc). Spain; 2003;39: 733–738.

104. Melzer M, Eykyn SJ, Gransden WR, Chinn S. Is methicillin-resistant Staphylococcus aureus more virulent than methicillin-susceptible S. aureus? A comparative cohort study of British patients with nosocomial infection and bacteremia. Clin Infect Dis. United States; 2003;37: 1453–1460. doi:10.1086/379321

105. Maugein J, Guillemot D, Dupont MJ, Fosse T, Laurans G, Roussel-Delvallez M, et al. Clinical and microbiological epidemiology of Streptococcus pneumoniae bacteremia in eight French counties. Clin Microbiol Infect. England; 2003;9: 280–288. doi:10.1046/j.1469-0691.2003.00520.x

106. Bantar C, Sartori B, Vesco E, Heft C, Saul M, Salamone F, et al. A hospitalwide intervention program to optimize the quality of antibiotic use: impact on prescribing practice, antibiotic consumption, cost savings, and bacterial resistance. Clin Infect Dis. United States; 2003;37: 180–186. doi:10.1086/375818

107. Jin R, Li X, Kong H, Li G, Wang W. [A case-control study on risk factors for nosocomial infection by extended-spectrum beta-lactamases-producing bacteria]. Zhonghua Yu Fang Yi Xue Za Zhi. China; 2003;37: 41–44.

108. Trindade PA, McCulloch JA, Oliveira GA, Mamizuka EM. Molecular techniques for MRSA typing: current issues and perspectives. Braz J Infect Dis. Brazil; 2003;7: 32–43.

109. Ellis SL, Finn P, Noone M, Leaper DJ. Eradication of methicillin-resistant Staphylococcus aureus from pressure sores using warming therapy. Surg Infect (Larchmt). United States; 2003;4: 53–55. doi:10.1089/109629603764655281

110. Martres P, Thibault M, Lemann F. [Significant decrease of rate and incidence of methicillin resistant Staphylococcus aureus in a French general hospital between 1999 and 2001]. Pathol Biol (Paris). France; 2003;51: 474–478.

111. Banbury MK. Experience in prevention of sternal wound infections in nasal carriers of Staphylococcus aureus. Surgery. United States; 2003;134: S18-22; quiz S23-5. doi:10.1016/s0039-6060(03)00389-1

112. Pilvinis V, Stirbiene I. [Ventilator associated pneumonia: risk factors, diagnosis, treatment and prevention]. Medicina (Kaunas). Switzerland; 2003;39: 1057–1064.

113. Chastre J. Infections due to Acinetobacter baumannii in the ICU. Semin Respir Crit Care Med. United States; 2003;24: 69–78. doi:10.1055/s-2003-37918

114. Zamir D, Polychuck I, Leibovitz I, Reitblat T, Zamir C, Scharf S. [Nosocomial infections in internal medicine departments]. Harefuah. Israel; 2003;142: 265-268,318.

115. Levin AS, Levy CE, Manrique AEI, Medeiros EAS, Costa SF. Severe nosocomial infections with imipenem-resistant Acinetobacter baumannii treated with ampicillin/sulbactam. Int J Antimicrob Agents. Netherlands; 2003;21: 58–62. doi:10.1016/s0924-8579(02)00276-5

116. Santucci SG, Gobara S, Santos CR, Fontana C, Levin AS. Infections in a burn intensive care unit: experience of seven years. J Hosp Infect. England; 2003;53: 6–13. doi:10.1053/jhin.2002.1340

117. Mody L, McNeil SA, Sun R, Bradley SE, Kauffman CA. Introduction of a waterless alcohol-based hand rub in a long-term-care facility. Infect Control Hosp Epidemiol. United States; 2003;24: 165–171. doi:10.1086/502185

118. Orsi GB, Raponi M, Sticca G, Branca L, Scalise E, Franchi C, et al. [Hospital infection surveillance in 5 Roman intensive care units]. Ann Ig. Italy; 2003;15: 23–34.

119. Nejjari N, Zerhouni F, Bouharrou A, Habzi A, Najdi T, Lahbabi M, et al. [Nosocomial infections caused by Acinetobacter: experience in a neonatal care unit in Casablanca]. Tunis Med. Tunisia; 2003;81: 121–125.

120. Kuo L-C, Yu C-J, Lee L-N, Wang J-L, Wang H-C, Hsueh P-R, et al. Clinical features of pandrug-resistant Acinetobacter baumannii bacteremia at a university hospital in Taiwan. J Formos Med Assoc. Singapore; 2003;102: 601–606.

121. Harbarth S, Garbino J, Pugin J, Romand JA, Lew D, Pittet D. Inappropriate initial antimicrobial therapy and its effect on survival in a clinical trial of immunomodulating therapy for severe sepsis. Am J Med. United States; 2003;115: 529–535. doi:10.1016/j.amjmed.2003.07.005

122. Katzenstein TL. Molecular biological assessment methods and understanding the course of the HIV infection. APMIS Suppl. Denmark; 2003; 1–37.

123. Suka M, Yoshida K, Takezawa J. Impact of intensive care unit-acquired infection on hospital mortality in Japan: A multicenter cohort study. Environ Health Prev Med. England; 2004;9: 53–57. doi:10.1007/BF02897932

124. Gordon A, Isaacs D. Late-onset infection and the role of antibiotic prescribing policies. Curr Opin Infect Dis. United States; 2004;17: 231–236.

125. Struelens MJ, Denis O, Rodriguez-Villalobos H. Microbiology of nosocomial infections: progress and challenges. Microbes Infect. France; 2004;6: 1043–1048. doi:10.1016/j.micinf.2004.05.015

126. Rupp ME, Fitzgerald T, Marion N, Helget V, Puumala S, Anderson JR, et al. Effect of silver-coated urinary catheters: efficacy, cost-effectiveness, and antimicrobial resistance. Am J Infect Control. United States; 2004;32: 445–450. doi:10.1016/j.ajic.2004.05.002

127. Hughes MG, Evans HL, Chong TW, Smith RL, Raymond DP, Pelletier SJ, et al. Effect of an intensive care unit rotating empiric antibiotic schedule on the development of hospital-acquired infections on the non-intensive care unit ward. Crit Care Med. United States; 2004;32: 53–60. doi:10.1097/01.CCM.0000104463.55423.EF

128. Thongpiyapoom S, Narong MN, Suwalak N, Jamulitrat S, Intaraksa P, Boonrat J, et al. Device-associated infections and patterns of antimicrobial resistance in a medical-surgical intensive care unit in a university hospital in Thailand. J Med Assoc Thai. Thailand; 2004;87: 819–824.

129. Bantar C, Vesco E, Heft C, Salamone F, Krayeski M, Gomez H, et al. Replacement of broad-spectrum cephalosporins by piperacillin-tazobactam: impact on sustained high rates of bacterial resistance. Antimicrob Agents Chemother. United States; 2004;48: 392–395. doi:10.1128/aac.48.2.392-395.2004

130. Taylor MD, Napolitano LM. Methicillin-resistant Staphylococcus aureus infections in vascular surgery: increasing prevalence. Surg Infect (Larchmt). United States; 2004;5: 180–187. doi:10.1089/sur.2004.5.180

131. Lazzari S, Allegranzi B, Concia E. Making hospitals safer: the need for a global strategy for infection control in health care settings. World Hosp Health Serv. England; 2004;40: 32,34,36-42.

132. Fukushima R, Inaba T, Iinuma H, Okinaga K. [Perioperative nosocomial infection preventive measures]. Nihon Geka Gakkai Zasshi. Japan; 2004;105: 696–701.

133. Taneja N, Emmanuel R, Chari PS, Sharma M. A prospective study of hospital-acquired infections in burn patients at a tertiary care referral centre in North India. Burns. Netherlands; 2004;30: 665–669. doi:10.1016/j.burns.2004.02.011

134. Ueki S, Kobayashi Y, Kayaba H, Kanda A, Saito N, Chiba T, et al. [Hospital infection control reduced medical care costs by saving dispensable antibiotics]. Rinsho Byori. Japan; 2004;52: 1001–1006.

135. Orrett FA. Resistance patterns among selective Gram-negative bacilli from an intensive care unit in Trinidad, West Indies. Saudi Med J. Saudi Arabia; 2004;25: 478–483.

136. Olvera-Caballero C, Victoria-Morales G. Neurovascular latissimus dorsi free-flap transfer for reconstruction of a major abdominal-wall defect in a 13-month-old child: late follow-up. J Reconstr Microsurg. United States; 2004;20: 237–240. doi:10.1055/s-2004-823111

137. Rowley S, Sinclair S. Working towards an NHS standard for aseptic non-touch technique. Nurs Times. England; 2004;100: 50–52.

138. Martins ST, Moreira M, Furtado GHC, Marino CGJ, Machado FR, Wey SB, et al. Application of control measures for infections caused by multi-resistant gram-negative bacteria in intensive care unit patients. Mem Inst Oswaldo Cruz. Brazil; 2004;99: 331–334. doi:10.1590/s0074-02762004000300017

139. Cowie SE, Ma I, Lee SK, Smith RM, Hsiang YN. Nosocomial MRSA infection in vascular surgery patients: impact on patient outcome. Vasc Endovascular Surg. United States; 2005;39: 327–334. doi:10.1177/153857440503900404

140. Hughes AJ, Ariffin N, Huat TL, Abdul Molok H, Hashim S, Sarijo J, et al. Prevalence of nosocomial infection and antibiotic use at a university medical center in Malaysia. Infect Control Hosp Epidemiol. United States; 2005;26: 100–104. doi:10.1086/502494

141. Sheng WH, Wang JT, Lu DCT, Chie WC, Chen YC, Chang SC. Comparative impact of hospital-acquired infections on medical costs, length of hospital stay and outcome between community hospitals and medical centres. J Hosp Infect. England; 2005;59: 205–214. doi:10.1016/j.jhin.2004.06.003

142. Cassettari VC, Strabelli T, Medeiros EAS. Staphylococcus aureus bacteremia: what is the impact of oxacillin resistance on mortality? Braz J Infect Dis. Brazil; 2005;9: 70–76. doi:/S1413-86702005000100012

143. Panhotra BR, Saxena AK, Al Mulhim AS. Prevalence of methicillin-resistant and methicillin-sensitive Staphylococcus aureus nasal colonization among patients at the time of admission to the hospital. Ann Saudi Med. Saudi Arabia; 2005;25: 304–308. doi:10.5144/0256-4947.2005.304

144. Orsi GB, Raponi M, Franchi C, Rocco M, Mancini C, Venditti M. Surveillance and infection control in an intensive care unit. Infect Control Hosp Epidemiol. United States; 2005;26: 321–325. doi:10.1086/502547

145. Gould IM. The clinical significance of methicillin-resistant Staphylococcus aureus. J Hosp Infect. England; 2005;61: 277–282. doi:10.1016/j.jhin.2005.06.014

146. Gastmeier P, Stamm-Balderjahn S, Hansen S, Nitzschke-Tiemann F, Zuschneid I, Groneberg K, et al. How outbreaks can contribute to prevention of nosocomial infection: analysis of 1,022 outbreaks. Infect Control Hosp Epidemiol. United States; 2005;26: 357–361. doi:10.1086/502552

147. Bauer TT, Lorenz J, Bodmann K-F, Vogel F. [Abbreviated guidelines for prevention, diagnostics, and therapy of nosocomial pneumonia]. Med Klin (Munich). Germany; 2005;100: 355–360. doi:10.1007/s00063-005-1045-x

148. Tasdelen Fisgin N, Darka O, Sunbul M, Coban AY, Sarikaya H, Altun B, et al. [Case report: a nosocomial infection caused by vancomycin resistant enterococcus]. Mikrobiyol Bul. Turkey; 2005;39: 351–355.

149. Jover Saenz A, Barcenilla Gaite F, Barbe Illa E, Garcia Gonzalez M, Lopez Salcedo R, Castellana Perello D, et al. [Nosocomial infection by multiresistant pathogens during one year in a secondary hospital: clinical an epidemiological analysis]. An Med Interna. Spain; 2005;22: 59–64.

150. Coello R, Charlett A, Wilson J, Ward V, Pearson A, Borriello P. Adverse impact of surgical site infections in English hospitals. J Hosp Infect. England; 2005;60: 93–103. doi:10.1016/j.jhin.2004.10.019

151. Resources to aid further learning about infection control. Nursing times. England; 2005. p. 51.

152. Guinan JL, McGuckin M, Shubin A, Tighe J. A descriptive review of malpractice claims for health care-acquired infections in Philadelphia. Am J Infect Control. United States; 2005;33: 310–312. doi:10.1016/j.ajic.2005.03.005

153. Coticchia JM, Dohar JE. Methicillin-resistant Staphylococcus aureus otorrhea after tympanostomy tube placement. Arch Otolaryngol Head Neck Surg. United States; 2005;131: 868–873. doi:10.1001/archotol.131.10.868

154. Hedrick TL, Sawyer RG. Health-care-associated infections and prevention. Surg Clin North Am. United States; 2005;85: 1137–52, ix. doi:10.1016/j.suc.2005.09.013

155. Shorr AF, Kollef MH. Ventilator-associated pneumonia: insights from recent clinical trials. Chest. United States; 2005;128: 583S-591S. doi:10.1378/chest.128.5_suppl_2.583S

156. Zhanel G, Hammond G. Key research issues in Clostridium difficile. Can J Infect Dis Med Microbiol = J Can des Mal Infect la Microbiol medicale. Egypt; 2005;16: 282–285. doi:10.1155/2005/326367

157. Datta A, Gardner A, Bell K. MRSA-free elective orthopaedic surgery.A dedicated elective orthopaedic ward free from MRSA Alexandra Hospital, Redditch, UK. Hip Int. United States; 2005;15: 171–176. doi:10.5301/HIP.2008.923

158. Jones C. Revised structures for the capsular polysaccharides from Staphylococcus aureus Types 5 and 8, components of novel glycoconjugate vaccines. Carbohydr Res. Netherlands; 2005;340: 1097–1106. doi:10.1016/j.carres.2005.02.001

159. Aragon D, Sole M Lou, Brown S. Outcomes of an infection prevention project focusing on hand hygiene and isolation practices. AACN Clin Issues. United States; 2005;16: 121–132.

160. Kumashi P, Girgawy E, Tarrand JJ, Rolston K V, Raad II, Safdar A. Streptococcus pneumoniae bacteremia in patients with cancer: disease characteristics and outcomes in the era of escalating drug resistance (1998-2002). Medicine (Baltimore). United States; 2005;84: 303–312. doi:10.1097/01.md.0000180045.26909.29

161. Vincent J-L, Brun-Buisson C, Niederman M, Haenni C, Harbarth S, Sprumont D, et al. Ethics roundtable debate: A patient dies from an ICU-acquired infection related to methicillin-resistant Staphylococcus aureus--how do you defend your case and your team? Crit Care. England; 2005;9: 5–9. doi:10.1186/cc3016

162. Beghetto E, Nielsen H V, Del Porto P, Buffolano W, Guglietta S, Felici F, et al. A combination of antigenic regions of Toxoplasma gondii microneme proteins induces protective immunity against oral infection with parasite cysts. J Infect Dis. United States; 2005;191: 637–645. doi:10.1086/427660

163. Chastre J. Conference summary: ventilator-associated pneumonia. Respiratory care. United States; 2005. pp. 975–983.

164. Fournier PE, Richet H. The epidemiology and control of Acinetobacter baumannii in health care facilities. Clin Infect Dis. United States; 2006;42: 692–699. doi:10.1086/500202

165. Kaye KS, Engemann JJ, Fulmer EM, Clark CC, Noga EM, Sexton DJ. Favorable impact of an infection control network on nosocomial infection rates in community hospitals. Infect Control Hosp Epidemiol. United States; 2006;27: 228–232. doi:10.1086/500371

166. Albrecht MC, Griffith ME, Murray CK, Chung KK, Horvath EE, Ward JA, et al. Impact of Acinetobacter infection on the mortality of burn patients. J Am Coll Surg. United States; 2006;203: 546–550. doi:10.1016/j.jamcollsurg.2006.06.013

167. Verdier R, Parer S, Jean-Pierre H, Dujols P, Picot M-C. Impact of an infection control program in an intensive care unit in France. Infect Control Hosp Epidemiol. United States; 2006;27: 60–66. doi:10.1086/499150

168. Wibbenmeyer L, Danks R, Faucher L, Amelon M, Latenser B, Kealey GP, et al. Prospective analysis of nosocomial infection rates, antibiotic use, and patterns of resistance in a burn population. J Burn Care Res. England; 2006;27: 152–160. doi:10.1097/01.BCR.0000203359.32756.F7

169. Gomes CC, Vormittag E, Santos CR, Levin AS. Nosocomial infection with cephalosporin-resistant Klebsiella pneumoniae is not associated with increased mortality. Infect Control Hosp Epidemiol. United States; 2006;27: 907–912. doi:10.1086/507276

170. McGregor JC, Perencevich EN, Furuno JP, Langenberg P, Flannery K, Zhu J, et al. Comorbidity risk-adjustment measures were developed and validated for studies of antibiotic-resistant infections. J Clin Epidemiol. United States; 2006;59: 1266–1273. doi:10.1016/j.jclinepi.2006.01.016

171. Gould IM. Costs of hospital-acquired methicillin-resistant Staphylococcus aureus (MRSA) and its control. Int J Antimicrob Agents. Netherlands; 2006;28: 379–384. doi:10.1016/j.ijantimicag.2006.09.001

172. Moreno CA, Rosenthal VD, Olarte N, Gomez WV, Sussmann O, Agudelo JG, et al. Device-associated infection rate and mortality in intensive care units of 9 Colombian hospitals: findings of the International Nosocomial Infection Control Consortium. Infect Control Hosp Epidemiol. United States; 2006;27: 349–356. doi:10.1086/503341

173. Southwood LL. Principles of antimicrobial therapy: what should we be using? Vet Clin North Am Equine Pract. United States; 2006;22: 279–96, vii. doi:10.1016/j.cveq.2006.04.004

174. Henshke-Bar-Meir R, Yinnon AM, Rudensky B, Attias D, Schlesinger Y, Raveh D. Assessment of the clinical significance of production of extended-spectrum beta-lactamases (ESBL) by Enterobacteriaceae. Infection. Germany; 2006;34: 66–74. doi:10.1007/s15010-006-4114-z

175. Flanders SA, Collard HR, Saint S. Nosocomial pneumonia: state of the science. Am J Infect Control. United States; 2006;34: 84–93. doi:10.1016/j.ajic.2005.07.003

176. Srinivasan S, Sheela D, Mathew R, Bazroy J, Kanungo R. Risk factors and associated problems in the management of infections with methicillin resistant Staphylococcus aureus. Indian J Med Microbiol. India; 2006;24: 182–185.

177. Hunter JD. Ventilator associated pneumonia. Postgrad Med J. England; 2006;82: 172–178. doi:10.1136/pgmj.2005.036905

178. Zavascki AP, Barth AL, Goncalves ALS, Moro ALD, Fernandes JF, Martins AF, et al. The influence of metallo-beta-lactamase production on mortality in nosocomial Pseudomonas aeruginosa infections. J Antimicrob Chemother. England; 2006;58: 387–392. doi:10.1093/jac/dkl239

179. Seybold U, Kourbatova E V, Johnson JG, Halvosa SJ, Wang YF, King MD, et al. Emergence of community-associated methicillin-resistant Staphylococcus aureus USA300 genotype as a major cause of health care-associated blood stream infections. Clin Infect Dis. United States; 2006;42: 647–656. doi:10.1086/499815

180. Morange-Saussier V, Giraudeau B, van der Mee N, Lermusiaux P, Quentin R. Nasal carriage of methicillin-resistant staphylococcus aureus in vascular surgery. Ann Vasc Surg. Netherlands; 2006;20: 767–772. doi:10.1007/s10016-006-9088-x

181. Ramirez JA. Controlling multiple-drug-resistant organisms at the hospital level. Expert Opin Pharmacother. England; 2006;7: 1449–1455. doi:10.1517/14656566.7.11.1449

182. Agodi A, Zarrilli R, Barchitta M, Anzaldi A, Di Popolo A, Mattaliano A, et al. Alert surveillance of intensive care unit-acquired Acinetobacter infections in a Sicilian hospital. Clin Microbiol Infect. England; 2006;12: 241–247. doi:10.1111/j.1469-0691.2005.01339.x

183. Svediene S, Ivaskevicius J. [Actualities of adults’ ventilator-associated pneumonia]. Medicina (Kaunas). Switzerland; 2006;42: 91–97.

184. Gilad J, Borer A. Prevention of catheter-related bloodstream infections in the neonatal intensive care setting. Expert Rev Anti Infect Ther. England; 2006;4: 861–873. doi:10.1586/14787210.4.5.861

185. Alvarez Lerma F, Palomar Martinez M, Olaechea Astigarraga P, Cerda Cerda E. [Nosocomial infection surveillance in critically ill patients in the intensive care units]. Medicina clinica. Spain; 2006. p. 798. doi:10.1157/13095822

186. Beam JW, Buckley B. Community-acquired methicillin-resistant Staphylococcus aureus: prevalence and risk factors. J Athl Train. United States; 2006;41: 337–340.

187. Dridi E, Chetoui A, Zaoui A. [Investigation of the prevalence of nosocomial infection in a Tunisian regional hospital]. Sante Publique. France; 2006;18: 187–194.

188. Brahmi N, Blel Y, Kouraichi N, Lahdhiri S, Thabet H, Hedhili A, et al. Impact of ceftazidime restriction on gram-negative bacterial resistance in an intensive care unit. J Infect Chemother Off J Japan Soc Chemother. Netherlands; 2006;12: 190–194. doi:10.1007/s10156-006-0452-0

189. de Lassence A, Hidri N, Timsit J-F, Joly-Guillou M-L, Thiery G, Boyer A, et al. Control and outcome of a large outbreak of colonization and infection with glycopeptide-intermediate Staphylococcus aureus in an intensive care unit. Clin Infect Dis. United States; 2006;42: 170–178. doi:10.1086/498898

190. Blot S, Depuydt P, Vandewoude K, De Bacquer D. Measuring the impact of multidrug resistance in nosocomial infection. Curr Opin Infect Dis. United States; 2007;20: 391–396. doi:10.1097/QCO.0b013e32818be6f7

191. Arda B, Sipahi OR, Yamazhan T, Tasbakan M, Pullukcu H, Tunger A, et al. Short-term effect of antibiotic control policy on the usage patterns and cost of antimicrobials, mortality, nosocomial infection rates and antibacterial resistance. J Infect. England; 2007;55: 41–48. doi:10.1016/j.jinf.2007.02.014

192. Bantar C, Alcazar G, Franco D, Vesco E, Salamone F, Izaguirre M, et al. Impact of antibiotic treatment on bacterial resistance rates from patients with hospital-acquired infection. J Chemother. England; 2007;19: 673–676. doi:10.1179/joc.2007.19.6.673

193. Jaureguy F, Carbonnelle E, Bonacorsi S, Clec’h C, Casassus P, Bingen E, et al. Host and bacterial determinants of initial severity and outcome of Escherichia coli sepsis. Clin Microbiol Infect. England; 2007;13: 854–862. doi:10.1111/j.1469-0691.2007.01775.x

194. Foglia EE, Fraser VJ, Elward AM. Effect of nosocomial infections due to antibiotic-resistant organisms on length of stay and mortality in the pediatric intensive care unit. Infect Control Hosp Epidemiol. United States; 2007;28: 299–306. doi:10.1086/512628

195. Foglia E, Meier MD, Elward A. Ventilator-associated pneumonia in neonatal and pediatric intensive care unit patients. Clin Microbiol Rev. United States; 2007;20: 409–25, table of contents. doi:10.1128/CMR.00041-06

196. Vonberg R-P, Chaberny IF, Kola A, Mattner F, Borgmann S, Dettenkofer M, et al. [Prevention and control of the spread of vancomycin-resistant enterococci: results of a workshop held by the German Society for Hygiene and Microbiology]. Der Anaesthesist. Germany; 2007. pp. 151–157. doi:10.1007/s00101-006-1123-3

197. Mehta A, Rosenthal VD, Mehta Y, Chakravarthy M, Todi SK, Sen N, et al. Device-associated nosocomial infection rates in intensive care units of seven Indian cities. Findings of the International Nosocomial Infection Control Consortium (INICC). J Hosp Infect. England; 2007;67: 168–174. doi:10.1016/j.jhin.2007.07.008

198. Ito W, Kobayashi N, Kayaba H, Takahashi T, Takeda M, Chiba T, et al. [Clinical evaluation after an introduction of our manual for antibiotic use during perioperative period and a notification policy of use of the antibiotics for MRSA]. Rinsho Byori. Japan; 2007;55: 224–229.

199. Lisboa T, Faria M, Hoher JA, Borges LAA, Gomez J, Schifelbain L, et al. [The prevalence of nosocomial infection in Intensive Care Units in the State of Rio Grande do Sul]. Rev Bras Ter intensiva. Brazil; 2007;19: 414–420.

200. Leone S, Fiore M, Felaco FM, Esposito S. [Early and timely therapy: when to interrupt antibiotic therapy in nosocomial acquired pneumonia?]. Le Infez Med. Italy; 2007;15: 16–23.

201. Surasarang K, Narksawat K, Danchaivijitr S, Siripanichgon K, Sujirarat D, Rongrungrueng Y, et al. Risk factors for multi-drug resistant Acinetobacter baumannii nosocomial infection. J Med Assoc Thai. Thailand; 2007;90: 1633–1639.

202. Sligl W, Taylor G, Gibney RN, Rennie R, Chui L. Methicillin-resistant Staphylococcus aureus in a Canadian intensive care unit: Delays in initiating effective therapy due to the low prevalence of infection. Can J Infect Dis Med Microbiol = J Can des Mal Infect la Microbiol medicale. Egypt; 2007;18: 139–143. doi:10.1155/2007/120987

203. Ghanem G, Hachem R, Jiang Y, Chemaly RF, Raad I. Outcomes for and risk factors associated with vancomycin-resistant Enterococcus faecalis and vancomycin-resistant Enterococcus faecium bacteremia in cancer patients. Infect Control Hosp Epidemiol. United States; 2007;28: 1054–1059. doi:10.1086/519932

204. Nguyen DM, Bancroft E, Mascola L, Guevara R, Yasuda L. Risk factors for neonatal methicillin-resistant Staphylococcus aureus infection in a well-infant nursery. Infect Control Hosp Epidemiol. United States; 2007;28: 406–411. doi:10.1086/513122

205. Celebi S, Hacimustafaoglu M, Ozdemir O, Ozakin C. Nosocomial Gram-positive bacterial infections in children: results of a 7 year study. Pediatr Int. Australia; 2007;49: 875–882. doi:10.1111/j.1442-200X.2007.02485.x

206. Gastmeier P, Sohr D, Geffers C, Behnke M, Ruden H. Risk factors for death due to nosocomial infection in intensive care unit patients: findings from the Krankenhaus Infektions Surveillance System. Infect Control Hosp Epidemiol. United States; 2007;28: 466–472. doi:10.1086/510810

207. Hamid MH, Zafar A, Maqbool S. Nosocomial bloodstream infection in a tertiary care paediatric intensive care unit. J Coll Physicians Surg Pak. Pakistan; 2007;17: 416–419. doi:07.2007/JCPSP.416419

208. Richards MJ, Russo PL. Surveillance of hospital-acquired infections in Australia--One Nation, Many States. J Hosp Infect. England; 2007;65 Suppl 2: 174–181. doi:10.1016/S0195-6701(07)60039-5

209. Deja M, Nachtigall I, Halle E, Kastrup M, Guill M Mac, Spies CD. [Strategies in the treatment of infections with antibiotics in intensive care medicine]. Anasthesiol Intensivmed Notfallmed Schmerzther. Germany; 2007;42: 108–115. doi:10.1055/s-2007-971162

210. Cooke FJ, Holmes AH. The missing care bundle: antibiotic prescribing in hospitals. Int J Antimicrob Agents. Netherlands; 2007;30: 25–29. doi:10.1016/j.ijantimicag.2007.03.003

211. Jarvis WR. The Lowbury Lecture. The United States approach to strategies in the battle against healthcare-associated infections, 2006: transitioning from benchmarking to zero tolerance and clinician accountability. The Journal of hospital infection. England; 2007. pp. 3–9. doi:10.1016/S0195-6701(07)60005-X

212. Bennett NJ, Bull AL, Dunt DR, Gurrin LC, Spelman DW, Russo PL, et al. MRSA infections in smaller hospitals, Victoria, Australia. Am J Infect Control. United States; 2007;35: 697–699. doi:10.1016/j.ajic.2006.12.011

213. Weber DJ, Rutala WA, Sickbert-Bennett EE, Samsa GP, Brown V, Niederman MS. Microbiology of ventilator-associated pneumonia compared with that of hospital-acquired pneumonia. Infect Control Hosp Epidemiol. United States; 2007;28: 825–831. doi:10.1086/518460

214. Blot S. Limiting the attributable mortality of nosocomial infection and multidrug resistance in intensive care units. Clin Microbiol Infect. England; 2008;14: 5–13. doi:10.1111/j.1469-0691.2007.01835.x

215. Ebnother C, Tanner B, Schmid F, La Rocca V, Heinzer I, Bregenzer T. Impact of an infection control program on the prevalence of nosocomial infections at a tertiary care center in Switzerland . Infect Control Hosp Epidemiol. United States; 2008;29: 38–43. doi:10.1086/524330

216. Nikbakht M, Nahaei MR, Akhi MT, Asgharzadeh M, Nikvash S. Molecular fingerprinting of meticillin-resistant Staphylococcus aureus strains isolated from patients and staff of two Iranian hospitals. J Hosp Infect. England; 2008;69: 46–55. doi:10.1016/j.jhin.2008.01.034

217. Rosenthal VD, Maki DG, Graves N. The International Nosocomial Infection Control Consortium (INICC): goals and objectives, description of surveillance methods, and operational activities. Am J Infect Control. United States; 2008;36: e1-12. doi:10.1016/j.ajic.2008.06.003

218. Daniels TL, Deppen S, Arbogast PG, Griffin MR, Schaffner W, Talbot TR. Mortality rates associated with multidrug-resistant Acinetobacter baumannii infection in surgical intensive care units. Infect Control Hosp Epidemiol. United States; 2008;29: 1080–1083. doi:10.1086/591456

219. Millar M, Coast J, Ashcroft R. Are meticillin-resistant Staphylococcus aureus bloodstream infection targets fair to those with other types of healthcare-associated infection or cost-effective? J Hosp Infect. England; 2008;69: 1–5. doi:10.1016/j.jhin.2008.01.035

220. Rosenthal VD. Device-associated nosocomial infections in limited-resources countries: findings of the International Nosocomial Infection Control Consortium (INICC). Am J Infect Control. United States; 2008;36: S171.e7-12. doi:10.1016/j.ajic.2008.10.009

221. Harbarth S, Fankhauser C, Schrenzel J, Christenson J, Gervaz P, Bandiera-Clerc C, et al. Universal screening for methicillin-resistant Staphylococcus aureus at hospital admission and nosocomial infection in surgical patients. JAMA. United States; 2008;299: 1149–1157. doi:10.1001/jama.299.10.1149

222. Enoch DA, Summers C, Brown NM, Moore L, Gillham MI, Burnstein RM, et al. Investigation and management of an outbreak of multidrug-carbapenem-resistant Acinetobacter baumannii in Cambridge, UK. J Hosp Infect. England; 2008;70: 109–118. doi:10.1016/j.jhin.2008.05.015

223. Halcomb EJ, Griffiths R, Fernandez R. The role of patient isolation and compliance with isolation practices in the control of nosocomial MRSA in acute care. Int J Evid Based Healthc. Australia; 2008;6: 206–224. doi:10.1111/j.1744-1609.2008.00089.x

224. Klevens RM, Edwards JR, Gaynes RP. The impact of antimicrobial-resistant, health care-associated infections on mortality in the United States. Clin Infect Dis. United States; 2008;47: 927–930. doi:10.1086/591698

225. Salomao R, Rosenthal VD, Grimberg G, Nouer S, Blecher S, Buchner-Ferreira S, et al. Device-associated infection rates in intensive care units of Brazilian hospitals: findings of the International Nosocomial Infection Control Consortium. Rev Panam Salud Publica. United States; 2008;24: 195–202. doi:10.1590/s1020-49892008000900006

226. Cuellar LE, Fernandez-Maldonado E, Rosenthal VD, Castaneda-Sabogal A, Rosales R, Mayorga-Espichan MJ, et al. Device-associated infection rates and mortality in intensive care units of Peruvian hospitals: findings of the International Nosocomial Infection Control Consortium. Rev Panam Salud Publica. United States; 2008;24: 16–24. doi:10.1590/s1020-49892008000700002

227. von Gottberg A, Klugman KP, Cohen C, Wolter N, de Gouveia L, du Plessis M, et al. Emergence of levofloxacin-non-susceptible Streptococcus pneumoniae and treatment for multidrug-resistant tuberculosis in children in South Africa: a cohort observational surveillance study. Lancet (London, England). England; 2008;371: 1108–1113. doi:10.1016/S0140-6736(08)60350-5

228. Halcomb EJ, Griffiths R, Fernandez R. The role of patient isolation and compliance with isolation practices in the control of nosocomial MRSA in acute care. JBI Libr Syst Rev. Australia; 2008;6: 234–264.

229. Pieracci F, Hydo L, Eachempati S, Pomp A, Shou J, Barie PS. Higher body mass index predicts need for insulin but not hyperglycemia, nosocomial infection, or death in critically ill surgical patients. Surg Infect (Larchmt). United States; 2008;9: 121–130. doi:10.1089/sur.2007.039

230. Pawar M, Mehta Y, Purohit A, Trehan N, Rosenthal VD. Resistance in gram-negative bacilli in a cardiac intensive care unit in India: risk factors and outcome. Ann Card Anaesth. India; 2008;11: 20–26.

231. Halcomb EJ, Griffiths R, Fernandez R. Role of MRSA reservoirs in the acute care setting. JBI Libr Syst Rev. Australia; 2008;6: 633–685.

232. van Rijen MM, Kluytmans JAJW. New approaches to prevention of staphylococcal infection in surgery. Curr Opin Infect Dis. United States; 2008;21: 380–384. doi:10.1097/QCO.0b013e328301391d

233. Behta M, Ross B, Chaudhry R. A comprehensive decision support system for the identification, monitoring and management of patients with multi-drug resistant organisms (MDRO). AMIA . Annu Symp proceedings AMIA Symp. United States; 2008; 1218.

234. Dembinski R, Rossaint R. [Ventilator-associated pneumonia]. Anaesthesist. Germany; 2008;57: 825–842. doi:10.1007/s00101-008-1415-x

235. Meier BM, Stone PW, Gebbie KM. Public health law for the collection and reporting of health care-associated infections. Am J Infect Control. United States; 2008;36: 537–551. doi:10.1016/j.ajic.2008.01.015

236. de Carvalho RH, Gontijo Filho PP. Epidemiologically relevant antimicrobial resistance phenotypes in pathogens isolated from critically ill patients in a Brazilian Universitary Hospital. Braz J Microbiol. Brazil; 2008;39: 623–630. doi:10.1590/S1517-83822008000400005

237. Palmer LB, Smaldone GC, Chen JJ, Baram D, Duan T, Monteforte M, et al. Aerosolized antibiotics and ventilator-associated tracheobronchitis in the intensive care unit. Crit Care Med. United States; 2008;36: 2008–2013. doi:10.1097/CCM.0b013e31817c0f9e

238. Loftus RW, Koff MD, Burchman CC, Schwartzman JD, Thorum V, Read ME, et al. Transmission of pathogenic bacterial organisms in the anesthesia work area. Anesthesiology. United States; 2008;109: 399–407. doi:10.1097/ALN.0b013e318182c855

239. Guriz H, Ciftci E, Ayberkin E, Aysev D, Ince E, Arsan S, et al. Stenotrophomonas maltophilia bacteraemia in Turkish children. Ann Trop Paediatr. England; 2008;28: 129–136. doi:10.1179/146532808X302152

240. Atack L, Luke R. Impact of an online course on infection control and prevention competencies. J Adv Nurs. England; 2008;63: 175–180. doi:10.1111/j.1365-2648.2008.04660.x

241. Rosenthal VD, Maki DG, Mehta A, Alvarez-Moreno C, Leblebicioglu H, Higuera F, et al. International Nosocomial Infection Control Consortium report, data summary for 2002-2007, issued January 2008. Am J Infect Control. United States; 2008;36: 627–637. doi:10.1016/j.ajic.2008.03.003

242. Roberts RR, Hota B, Ahmad I, Scott RD 2nd, Foster SD, Abbasi F, et al. Hospital and societal costs of antimicrobial-resistant infections in a Chicago teaching hospital: implications for antibiotic stewardship. Clin Infect Dis. United States; 2009;49: 1175–1184. doi:10.1086/605630

243. Madani N, Rosenthal VD, Dendane T, Abidi K, Zeggwagh AA, Abouqal R. Health-care associated infections rates, length of stay, and bacterial resistance in an intensive care unit of Morocco: findings of the International Nosocomial Infection Control Consortium (INICC). Int Arch Med. England; 2009;2: 29. doi:10.1186/1755-7682-2-29

244. Lautenbach E, Synnestvedt M, Weiner MG, Bilker WB, Vo L, Schein J, et al. Epidemiology and impact of imipenem resistance in Acinetobacter baumannii. Infect Control Hosp Epidemiol. United States; 2009;30: 1186–1192. doi:10.1086/648450

245. Cheong HS, Kang C-I, Lee JA, Moon SY, Joung MK, Chung DR, et al. Clinical significance and outcome of nosocomial acquisition of spontaneous bacterial peritonitis in patients with liver cirrhosis. Clin Infect Dis. United States; 2009;48: 1230–1236. doi:10.1086/597585

246. Larue A, Loos-Ayav C, Jay N, Commun N, Rabaud C, Bollaert P-E. [Impact on morbidity and costs of methicillin-resistant Staphylococcus aureus nosocomial pneumonia in intensive care patients]. Presse Med. France; 2009;38: 25–33. doi:10.1016/j.lpm.2008.06.005

247. Wibbenmeyer L, Appelgate D, Williams I, Light T, Latenser B, Lewis R, et al. Effectiveness of universal screening for vancomycin-resistant enterococcus and methicillin-resistant Staphylococcus aureus on admission to a burn-trauma step-down unit. J Burn Care Res. England; 2009;30: 648–656. doi:10.1097/BCR.0b013e3181abff7e

248. Rodrigues PM de A, Carmo Neto E do, Santos LR de C, Knibel MF. Ventilator-associated pneumonia: epidemiology and impact on the clinical evolution of ICU patients. J Bras Pneumol publicacao Of da Soc Bras Pneumol e Tisilogia. Brazil; 2009;35: 1084–1091. doi:10.1590/s1806-37132009001100005

249. Carlet J, Astagneau P, Brun-Buisson C, Coignard B, Salomon V, Tran B, et al. French national program for prevention of healthcare-associated infections and antimicrobial resistance, 1992-2008: positive trends, but perseverance needed. Infect Control Hosp Epidemiol. United States; 2009;30: 737–745. doi:10.1086/598682

250. Wang Z, Cao B, Liu Y, Gu L, Wang C. Investigation of the prevalence of patients co-colonized or infected with methicillin-resistant Staphylococcus aureus and vancomycin-resistant enterococci in China: a hospital-based study. Chin Med J (Engl). China; 2009;122: 1283–1288.

251. Freixas N, Salles M, Garcia L. [Changes in nosocomial infection control: new challenges and responsibilities for the infection control nurse]. Enferm Infecc Microbiol Clin. Spain; 2009;27: 285–289. doi:10.1016/j.eimc.2008.01.004

252. Shrestha B, Pokhrel BM, Mohapatra TM. Antibiotic susceptibility pattern of nosocomial isolates of staphylococcus aureus in a tertiary care hospital, Nepal. JNMA J Nepal Med Assoc. Nepal; 2009;48: 234–238.

253. Murray CK, Yun HC, Griffith ME, Thompson B, Crouch HK, Monson LS, et al. Recovery of multidrug-resistant bacteria from combat personnel evacuated from Iraq and Afghanistan at a single military treatment facility. Mil Med. England; 2009;174: 598–604. doi:10.7205/milmed-d-03-8008

254. Pofahl WE, Goettler CE, Ramsey KM, Cochran MK, Nobles DL, Rotondo MF. Active surveillance screening of MRSA and eradication of the carrier state decreases surgical-site infections caused by MRSA. J Am Coll Surg. United States; 2009;208: 981–988. doi:10.1016/j.jamcollsurg.2008.12.025

255. Lu P-L, Siu LK, Chen T-C, Ma L, Chiang W-G, Chen Y-H, et al. Methicillin-resistant Staphylococcus aureus and Acinetobacter baumannii on computer interface surfaces of hospital wards and association with clinical isolates. BMC Infect Dis. England; 2009;9: 164. doi:10.1186/1471-2334-9-164

256. Desenclos J-C. RAISIN - a national programme for early warning, investigation and surveillance of healthcare-associated infection in France. Euro Surveill Bull Eur sur les Mal Transm = Eur Commun Dis Bull. Sweden; 2009;14.

257. Lee C-Y, Chen P-Y, Huang F-L, Lin C-F. Microbiologic spectrum and susceptibility pattern of clinical isolates from the pediatric intensive care unit in a single medical center - 6 years’ experience. J Microbiol Immunol Infect. England; 2009;42: 160–165.

258. Kirdar S, Arslan U, Tuncer I, Findik D, Bozdogan B. [Investigation of the clonality and Panton-Valentine leukocidin toxin among nosocomial methicillin-resistant Staphylococcus aureus strains]. Mikrobiyol Bul. Turkey; 2009;43: 529–533.

259. Furtado GHC, Bergamasco MD, Menezes FG, Marques D, Silva A, Perdiz LB, et al. Imipenem-resistant Pseudomonas aeruginosa infection at a medical-surgical intensive care unit: risk factors and mortality. J Crit Care. United States; 2009;24: 625.e9–14. doi:10.1016/j.jcrc.2009.03.006

260. Ahern JW, Alston WK. Use of longitudinal surveillance data to assess the effectiveness of infection control in critical care. Infect Control Hosp Epidemiol. United States; 2009;30: 1109–1112. doi:10.1086/644752

261. Niven DJ, Laupland KB, Gregson DB, Church DL. Epidemiology of Staphylococcus aureus nasal colonization and influence on outcome in the critically ill. J Crit Care. United States; 2009;24: 583–589. doi:10.1016/j.jcrc.2008.10.004

262. Lithgow AE, Kilalang C. Outbreak of nosocomial sepsis in the Special Care Nursery at Port Moresby General Hospital due to multiresistant Klebsiella pneumoniae: high impact on mortality. P N G Med J. Papua New Guinea; 2009;52: 28–34.

263. Mowery NT, Carnevale RJ, Gunter OL, Norris PR, Dossett LA, Dortch MJ, et al. Insulin resistance heralds positive cultures after severe injury. Surg Infect (Larchmt). United States; 2009;10: 503–509. doi:10.1089/sur.2008.086

264. Vaduva DB, Muntean D, Licker M, Velimirovici D, Radulescu M, Vaduva MB, et al. [Gram-positive cocci isolated from urocultures of ambulatory and hospitalized patients with surgical risk]. Bacteriol Virusol Parazitol Epidemiol. Romania; 2009;54: 23–29.

265. Lederer JWJ, Best D, Hendrix V. A comprehensive hand hygiene approach to reducing MRSA health care-associated infections. Jt Comm J Qual patient Saf. Netherlands; 2009;35: 180–185.

266. Kerstein RL, Fellowes C. Novel fit for purpose single use tourniquet: best of both worlds. J Med Eng Technol. England; 2009;33: 475–480. doi:10.1080/03091900902952667

267. Basak S, Mallick SK, Bose S. Extended spectrum beta-lactamase producing Escherichia coli and Klebsiella pneumoniae isolated in a rural hospital. J Indian Med Assoc. India; 2009;107: 855–858.

268. Hassanzadeh P, Motamedifar M, Hadi N. Prevalent bacterial infections in intensive care units of Shiraz University of medical sciences teaching hospitals, Shiraz, Iran. Jpn J Infect Dis. Japan; 2009;62: 249–253.

269. Shete VB, Ghadage DP, Muley VA, Bhore A V. Acinetobacter septicemia in neonates admitted to intensive care units. J Lab Physicians. India; 2009;1: 73–76. doi:10.4103/0974-2727.59704

270. Ben-Ami R, Rodriguez-Bano J, Arslan H, Pitout JDD, Quentin C, Calbo ES, et al. A multinational survey of risk factors for infection with extended-spectrum beta-lactamase-producing enterobacteriaceae in nonhospitalized patients. Clin Infect Dis. United States; 2009;49: 682–690. doi:10.1086/604713

271. Giamarellou H. Multidrug-resistant Gram-negative bacteria: how to treat and for how long. Int J Antimicrob Agents. Netherlands; 2010;36 Suppl 2: S50-4. doi:10.1016/j.ijantimicag.2010.11.014

272. Nguyen GC, Patel H, Chong RY. Increased prevalence of and associated mortality with methicillin-resistant Staphylococcus aureus among hospitalized IBD patients. Am J Gastroenterol. United States; 2010;105: 371–377. doi:10.1038/ajg.2009.581

273. Roberts RR, Scott RD 2nd, Hota B, Kampe LM, Abbasi F, Schabowski S, et al. Costs attributable to healthcare-acquired infection in hospitalized adults and a comparison of economic methods. Med Care. United States; 2010;48: 1026–1035. doi:10.1097/MLR.0b013e3181ef60a2

274. Cummings KL, Anderson DJ, Kaye KS. Hand hygiene noncompliance and the cost of hospital-acquired methicillin-resistant Staphylococcus aureus infection. Infect Control Hosp Epidemiol. United States; 2010;31: 357–364. doi:10.1086/651096

275. Ion-Nedelcu N, Lambru K, Luminita R, Manta C. [Risk of hospital death in nosocomial infection with multi-drug resistant A. baumanii or P. aeruginosa]. Bacteriol Virusol Parazitol Epidemiol. Romania; 2010;55: 29–33.

276. Al-Talib HI, Yean CY, Al-Jashamy K, Hasan H. Methicillin-resistant Staphylococcus aureus nosocomial infection trends in Hospital Universiti Sains Malaysia during 2002-2007. Ann Saudi Med. Saudi Arabia; 2010;30: 358–363. doi:10.4103/0256-4947.67077

277. Paul M, Kariv G, Goldberg E, Raskin M, Shaked H, Hazzan R, et al. Importance of appropriate empirical antibiotic therapy for methicillin-resistant Staphylococcus aureus bacteraemia. J Antimicrob Chemother. England; 2010;65: 2658–2665. doi:10.1093/jac/dkq373

278. Korczak D, Schoffmann C. Medical and health economic evaluation of prevention- and control measures related to MRSA infections or -colonisations at hospitals. GMS Health Technol Assess. Germany; 2010;6: Doc04. doi:10.3205/hta000082

279. Jacoby TS, Kuchenbecker RS, Dos Santos RP, Magedanz L, Guzatto P, Moreira LB. Impact of hospital-wide infection rate, invasive procedures use and antimicrobial consumption on bacterial resistance inside an intensive care unit. J Hosp Infect. England; 2010;75: 23–27. doi:10.1016/j.jhin.2009.11.021

280. de Oliveira AC, Silva RS, Diaz MEP, Iquiapaza RA. Bacterial resistance and mortality in an intensive care unit. Rev Lat Am Enfermagem. Brazil; 2010;18: 1152–1160. doi:10.1590/s0104-11692010000600016

281. Gao Y, Yang Y, Wu Y, Cao W, Zhou Q. [Change of ESBLs-KPN and ESBLs-ECO after antimicrobial intervention]. Zhong Nan Da Xue Xue Bao Yi Xue Ban. China; 2010;35: 165–170. doi:10.3969/j.issn.1672-7347.2010.02.013

282. Fraser S, Brady RR, Graham C, Paterson-Brown S, Gibb AP. Methicillin-resistant Staphylococcus aureus in surgical patients: identification of high-risk populations for the development of targeted screening programmes. Ann R Coll Surg Engl. England; 2010;92: 311–315. doi:10.1308/003588410X12628812459698

283. Gagne D, Bedard G, Maziade PJ. Systematic patients’ hand disinfection: impact on meticillin-resistant Staphylococcus aureus infection rates in a community hospital. J Hosp Infect. England; 2010;75: 269–272. doi:10.1016/j.jhin.2010.02.028

284. Astagneau P, L’Heriteau F. Surveillance of surgical-site infections: impact on quality of care and reporting dilemmas. Curr Opin Infect Dis. United States; 2010;23: 306–310. doi:10.1097/QCO.0b013e32833ae7e3

285. Takayama Y, Okamoto R, Sunakawa K. Definite infective endocarditis: clinical and microbiological features of 155 episodes in one Japanese university hospital. J Formos Med Assoc. Singapore; 2010;109: 788–799. doi:10.1016/S0929-6646(10)60124-6

286. Dent LL, Marshall DR, Pratap S, Hulette RB. Multidrug resistant Acinetobacter baumannii: a descriptive study in a city hospital. BMC Infect Dis. England; 2010;10: 196. doi:10.1186/1471-2334-10-196

287. Sartelli M. A focus on intra-abdominal infections. World J Emerg Surg. England; 2010;5: 9. doi:10.1186/1749-7922-5-9

288. Milstone AM, Bryant KA, Huskins WC, Zerr DM. The past, present, and future of healthcare-associated infection prevention in pediatrics: multidrug-resistant organisms. Infect Control Hosp Epidemiol. United States; 2010;31 Suppl 1: S18-21. doi:10.1086/656001

289. Joseph NM, Sistla S, Dutta TK, Badhe AS, Parija SC. Ventilator-associated pneumonia: a review. Eur J Intern Med. Netherlands; 2010;21: 360–368. doi:10.1016/j.ejim.2010.07.006

290. Dogru A, Sargin F, Celik M, Sagiroglu AE, Goksel MM, Sayhan H. The rate of device-associated nosocomial infections in a medical surgical intensive care unit of a training and research hospital in Turkey: one-year outcomes. Jpn J Infect Dis. Japan; 2010;63: 95–98.

291. Kontopoulou K, Protonotariou E, Vasilakos K, Kriti M, Koteli A, Antoniadou E, et al. Hospital outbreak caused by Klebsiella pneumoniae producing KPC-2 beta-lactamase resistant to colistin. J Hosp Infect. England; 2010;76: 70–73. doi:10.1016/j.jhin.2010.03.021

292. Ben-David D, Maor Y, Keller N, Regev-Yochay G, Tal I, Shachar D, et al. Potential role of active surveillance in the control of a hospital-wide outbreak of carbapenem-resistant Klebsiella pneumoniae infection. Infect Control Hosp Epidemiol. United States; 2010;31: 620–626. doi:10.1086/652528

293. Kullar R, Davis SL, Levine DP, Rybak MJ. Impact of vancomycin exposure on outcomes in patients with methicillin-resistant Staphylococcus aureus bacteremia: support for consensus guidelines suggested targets. Clin Infect Dis. United States; 2011;52: 975–981. doi:10.1093/cid/cir124

294. Kohlenberg A, Schwab F, Behnke M, Gastmeier P. Screening and control of methicillin-resistant Staphylococcus aureus in 186 intensive care units: different situations and individual solutions. Crit Care. England; 2011;15: R285. doi:10.1186/cc10571

295. Anunnatsiri S, Tonsawan P. Risk factors and clinical outcomes of multidrug-resistant Acinetobacter baumannii bacteremia at a university hospital in Thailand. Southeast Asian J Trop Med Public Health. Thailand; 2011;42: 693–703.

296. Shorr AF, Micek ST, Welch EC, Doherty JA, Reichley RM, Kollef MH. Inappropriate antibiotic therapy in Gram-negative sepsis increases hospital length of stay. Crit Care Med. United States; 2011;39: 46–51. doi:10.1097/CCM.0b013e3181fa41a7

297. Kassakian SZ, Mermel LA, Jefferson JA, Parenteau SL, Machan JT. Impact of chlorhexidine bathing on hospital-acquired infections among general medical patients. Infect Control Hosp Epidemiol. United States; 2011;32: 238–243. doi:10.1086/658334

298. Eseonu KC, Middleton SD, Eseonu CC. A retrospective study of risk factors for poor outcomes in methicillin-resistant Staphylococcus aureus (MRSA) infection in surgical patients. J Orthop Surg Res. England; 2011;6: 25. doi:10.1186/1749-799X-6-25

299. Navoa-Ng JA, Berba R, Galapia YA, Rosenthal VD, Villanueva VD, Tolentino MC V, et al. Device-associated infections rates in adult, pediatric, and neonatal intensive care units of hospitals in the Philippines: International Nosocomial Infection Control Consortium (INICC) findings. Am J Infect Control. United States; 2011;39: 548–554. doi:10.1016/j.ajic.2010.10.018

300. Dortch MJ, Fleming SB, Kauffmann RM, Dossett LA, Talbot TR, May AK. Infection reduction strategies including antibiotic stewardship protocols in surgical and trauma intensive care units are associated with reduced resistant gram-negative healthcare-associated infections. Surg Infect (Larchmt). United States; 2011;12: 15–25. doi:10.1089/sur.2009.059

301. Nguyen GC, Leung W, Weizman A V. Increased risk of vancomycin-resistant enterococcus (VRE) infection among patients hospitalized for inflammatory bowel disease in the United States. Inflamm Bowel Dis. England; 2011;17: 1338–1342. doi:10.1002/ibd.21519

302. Song K-H, Kang YM, Sin H-Y, Yoon SW, Seo H-K, Kwon S, et al. Outcome of cefazolin prophylaxis for total knee arthroplasty at an institution with high prevalence of methicillin-resistant Staphylococcus aureus infection. Int J Infect Dis. Canada; 2011;15: e867-70. doi:10.1016/j.ijid.2011.09.009

303. Dettenkofer M, Ammon A, Astagneau P, Dancer SJ, Gastmeier P, Harbarth S, et al. Infection control--a European research perspective for the next decade. The Journal of hospital infection. England; 2011. pp. 7–10. doi:10.1016/j.jhin.2010.07.025

304. Tsuji BT, MacLean RD, Dresser LD, McGavin MJ, Simor AE. Impact of accessory gene regulator (agr) dysfunction on vancomycin pharmacodynamics among Canadian community and health-care associated methicillin-resistant Staphylococcus aureus. Ann Clin Microbiol Antimicrob. England; 2011;10: 20. doi:10.1186/1476-0711-10-20

305. Eryilmaz M, Akin A, Arikan Akan O. [Investigation of the efficacy of some disinfectants against nosocomial Staphylococcus aureus and Enterococcus spp. isolates]. Mikrobiyol Bul. Turkey; 2011;45: 454–460.

306. Gomes MZR, Machado CR, da Conceicao M de S, Ortega JA, Neves SMFM, Lourenco MC da S, et al. Outbreaks, persistence, and high mortality rates of multiresistant Pseudomonas aeruginosa infections in a hospital with AIDS-predominant admissions. Braz J Infect Dis. Brazil; 2011;15: 312–322.

307. Lin J-N, Chen Y-H, Chang L-L, Lai C-H, Lin H-L, Lin H-H. Clinical characteristics and outcomes of patients with extended-spectrum beta-lactamase-producing bacteremias in the emergency department. Intern Emerg Med. Italy; 2011;6: 547–555. doi:10.1007/s11739-011-0707-3

308. Tao L, Hu B, Rosenthal VD, Gao X, He L. Device-associated infection rates in 398 intensive care units in Shanghai, China: International Nosocomial Infection Control Consortium (INICC) findings. Int J Infect Dis. Canada; 2011;15: e774-80. doi:10.1016/j.ijid.2011.06.009

309. Raad II, Mohamed JA, Reitzel RA, Jiang Y, Dvorak TL, Ghannoum MA, et al. The prevention of biofilm colonization by multidrug-resistant pathogens that cause ventilator-associated pneumonia with antimicrobial-coated endotracheal tubes. Biomaterials. Netherlands; 2011;32: 2689–2694. doi:10.1016/j.biomaterials.2010.12.015

310. Brown DQ. Disposable vs reusable electrocardiography leads in development of and cross-contamination by resistant bacteria. Crit Care Nurse. United States; 2011;31: 62–68. doi:10.4037/ccn2011874

311. Chin BS, Kim MS, Han SH, Shin SY, Choi HK, Chae YT, et al. Risk factors of all-cause in-hospital mortality among Korean elderly bacteremic urinary tract infection (UTI) patients. Arch Gerontol Geriatr. Netherlands; 2011;52: e50-5. doi:10.1016/j.archger.2010.05.011

312. Azimi L, Motevallian A, Ebrahimzadeh Namvar A, Asghari B, Lari AR. Nosocomial infections in burned patients in motahari hospital, tehran, iran. Dermatol Res Pract. Egypt; 2011;2011: 436952. doi:10.1155/2011/436952

313. Geva A, Wright SB, Baldini LM, Smallcomb JA, Safran C, Gray JE. Spread of methicillin-resistant Staphylococcus aureus in a large tertiary NICU: network analysis. Pediatrics. United States; 2011;128: e1173-80. doi:10.1542/peds.2010-2562

314. Blazejewski C, Guerry M-J, Preau S, Durocher A, Nseir S. New methods to clean ICU rooms. Infect Disord Drug Targets. United Arab Emirates; 2011;11: 365–375.

315. Wolkewitz M, Frank U, Philips G, Schumacher M, Davey P. Mortality associated with in-hospital bacteraemia caused by Staphylococcus aureus: a multistate analysis with follow-up beyond hospital discharge. J Antimicrob Chemother. England; 2011;66: 381–386. doi:10.1093/jac/dkq424

316. Bonten MJM. Healthcare epidemiology: Ventilator-associated pneumonia: preventing the inevitable. Clin Infect Dis. United States; 2011;52: 115–121. doi:10.1093/cid/ciq075

317. Wu D, Cai J, Liu J. Risk factors for the acquisition of nosocomial infection with carbapenem-resistant Klebsiella pneumoniae. South Med J. United States; 2011;104: 106–110. doi:10.1097/SMJ.0b013e318206063d

318. Garcia Hernandez A, Garcia-Vazquez E, Gomez Gomez J, Canteras M, Hernandez-Torres A, Ruiz Gomez J. [Predictive factors of ESBL versus non-ESBL Escherichia coli bacteraemia and influence of resistance on the mortality of the patients]. Med Clin (Barc). Spain; 2011;136: 56–60. doi:10.1016/j.medcli.2010.05.014

319. Kavanagh KT. Financial incentives to promote health care quality: the hospital acquired conditions nonpayment policy. Soc Work Public Health. United States; 2011;26: 524–541. doi:10.1080/19371918.2011.533554

320. Rivera AM, Boucher HW. Current concepts in antimicrobial therapy against select gram-positive organisms: methicillin-resistant Staphylococcus aureus, penicillin-resistant pneumococci, and vancomycin-resistant enterococci. Mayo Clin Proc. England; 2011;86: 1230–1243. doi:10.4065/mcp.2011.0514

321. Hernandez-Torres A, Garcia-Vazquez E, Gomez J, Canteras M, Ruiz J, Yague G. Multidrug and carbapenem-resistant Acinetobacter baumannii infections: Factors associated with mortality. Med Clin (Barc). Spain; 2012;138: 650–655. doi:10.1016/j.medcli.2011.06.024

322. Xie C, Taylor DM, Howden BP, Charles PGP. Comparison of the bacterial isolates and antibiotic resistance patterns of elderly nursing home and general community patients. Intern Med J. Australia; 2012;42: e157-64. doi:10.1111/j.1445-5994.2011.02436.x

323. Kanj S, Kanafani Z, Sidani N, Alamuddin L, Zahreddine N, Rosenthal V. International nosocomial infection control consortium findings of device-associated infections rate in an intensive care unit of a lebanese university hospital. J Glob Infect Dis. India; 2012;4: 15–21. doi:10.4103/0974-777X.93755

324. Ho C, Lau A, Cimon K, Farrah K, Gardam M. No Title. Ottawa (ON); 2012.

325. Kubler A, Duszynska W, Rosenthal VD, Fleischer M, Kaiser T, Szewczyk E, et al. Device-associated infection rates and extra length of stay in an intensive care unit of a university hospital in Wroclaw, Poland: International Nosocomial Infection Control Consortium’s (INICC) findings. J Crit Care. United States; 2012;27: 105.e5–10. doi:10.1016/j.jcrc.2011.05.018

326. Alp E, Coruh A, Gunay GK, Yontar Y, Doganay M. Risk factors for nosocomial infection and mortality in burn patients: 10 years of experience at a university hospital. J Burn Care Res. England; 2012;33: 379–385. doi:10.1097/BCR.0b013e318234966c

327. Monistrol O, Calbo E, Riera M, Nicolas C, Font R, Freixas N, et al. Impact of a hand hygiene educational programme on hospital-acquired infections in medical wards. Clin Microbiol Infect. England; 2012;18: 1212–1218. doi:10.1111/j.1469-0691.2011.03735.x

328. Chen Y-Y, Chen L-Y, Lin S-Y, Chou P, Liao S-Y, Wang F-D. Surveillance on secular trends of incidence and mortality for device-associated infection in the intensive care unit setting at a tertiary medical center in Taiwan, 2000-2008: a retrospective observational study. BMC Infect Dis. England; 2012;12: 209. doi:10.1186/1471-2334-12-209

329. Kanerva M, Ollgren J, Hakanen AJ, Lyytikainen O. Estimating the burden of healthcare-associated infections caused by selected multidrug-resistant bacteria Finland, 2010. Antimicrob Resist Infect Control. England; 2012;1: 33. doi:10.1186/2047-2994-1-33

330. Conrick-Martin I, O’Gorman J, Lenehan D, Oshodi D, Scanlon N, O’Brien S, et al. Nosocomial infections in a cohort of extracorporeal life support patients. Crit Care Resusc. Australia; 2012;14: 198–201.

331. Gastmeier P, Schwab F, Behnke M, Geffers C. Decreasing healthcare-associated infections (HAI) is an efficient method to decrease healthcare-associated Methicillin-resistant S.aureus (MRSA) infections Antimicrobial resistance data from the German national nosocomial surveillance system KISS. Antimicrob Resist Infect Control. England; 2012;1: 3. doi:10.1186/2047-2994-1-3

332. Chen C-Y, Chen Y-H, Lu P-L, Lin W-R, Chen T-C, Lin C-Y. Proteus mirabilis urinary tract infection and bacteremia: risk factors, clinical presentation, and outcomes. J Microbiol Immunol Infect. England; 2012;45: 228–236. doi:10.1016/j.jmii.2011.11.007

333. Hyllienmark P, Martling C-R, Struwe J, Petersson J. Pathogens in the lower respiratory tract of intensive care unit patients: impact of duration of hospital care and mechanical ventilation. Scand J Infect Dis. England; 2012;44: 444–452. doi:10.3109/00365548.2011.645504

334. El-Kholy A, Saied T, Gaber M, Younan MA, Haleim MMA, El-Sayed H, et al. Device-associated nosocomial infection rates in intensive care units at Cairo University hospitals: first step toward initiating surveillance programs in a resource-limited country. Am J Infect Control. United States; 2012;40: e216-20. doi:10.1016/j.ajic.2011.12.010

335. Bereket W, Hemalatha K, Getenet B, Wondwossen T, Solomon A, Zeynudin A, et al. Update on bacterial nosocomial infections. Eur Rev Med Pharmacol Sci. Italy; 2012;16: 1039–1044.

336. Elhassan HA, Dixon T. MRSA contaminated venepuncture tourniquets in clinical practice. Postgrad Med J. England; 2012;88: 194–197. doi:10.1136/postgradmedj-2011-130411

337. Kaier K, Wilson C, Hulscher M, Wollersheim H, Huis A, Borg M, et al. Implementing strategic bundles for infection prevention and management. Infection. Germany; 2012;40: 225–228. doi:10.1007/s15010-011-0186-5

338. Daneman N, Elligsen M, Walker SAN, Simor A. Duration of hospital admission and the need for empirical antipseudomonal therapy. J Clin Microbiol. United States; 2012;50: 2695–2701. doi:10.1128/JCM.00758-12

339. Bolat F, Uslu S, Bolat G, Comert S, Can E, Bulbul A, et al. Healthcare-associated infections in a Neonatal Intensive Care Unit in Turkey. Indian Pediatr. India; 2012;49: 951–957. doi:10.1007/s13312-012-0249-4

340. Gomes MZR, Oliveira RVC de, Machado CR, Conceicao M de S da, Souza CV de, Lourenco MC da S, et al. Factors associated with epidemic multiresistant Pseudomonas aeruginosa infections in a hospital with AIDS-predominant admissions. Braz J Infect Dis. Brazil; 2012;16: 219–225.

341. Peterson KE, Hacek DM, Robicsek A, Thomson RBJ, Peterson LR. Electronic surveillance for infectious disease trend analysis following a quality improvement intervention. Infect Control Hosp Epidemiol. United States; 2012;33: 790–795. doi:10.1086/666625

342. Scawn N, Saul D, Pathak D, Matata B, Kemp I, Stables R, et al. A pilot randomised controlled trial in intensive care patients comparing 7 days’ treatment with empirical antibiotics with 2 days’ treatment for hospital-acquired infection of unknown origin. Health Technol Assess. England; 2012;16: i–xiii, 1–70. doi:10.3310/hta16360

343. Wiedenkeller B, Modert F, Karleskind I. [The dependence level of the elderly person influences the risk of infection]. Bull Soc Sci Med Grand Duche Luxemb. Luxembourg; 2012; 32–42.

344. Naidu P, Smith S. A review of 11 years of Stenotrophomonas maltophilia blood isolates at a tertiary care institute in Canada. Can J Infect Dis Med Microbiol = J Can des Mal Infect la Microbiol medicale. Egypt; 2012;23: 165–169. doi:10.1155/2012/762571

345. Wang L, Sun Y, Song W, Zhang Z, Liu C. [Changes of drug-resistance of Pseudomonas aeruginosa in pediatric intensive care unit]. Zhonghua er ke za zhi = Chinese J Pediatr. China; 2012;50: 657–663.

346. Ikeda Y, Mamiya T, Nishiyama H, Koseki T, Mouri A, Nabeshima T. Risk factors for extended-spectrum beta-lactamase-producing Escherichia coli infection in hospitalized patients. Nagoya J Med Sci. Japan; 2012;74: 105–114.

347. Rosenthal VD, Bijie H, Maki DG, Mehta Y, Apisarnthanarak A, Medeiros EA, et al. International Nosocomial Infection Control Consortium (INICC) report, data summary of 36 countries, for 2004-2009. Am J Infect Control. United States; 2012;40: 396–407. doi:10.1016/j.ajic.2011.05.020

348. Vital signs: carbapenem-resistant Enterobacteriaceae. MMWR Morb Mortal Wkly Rep. United States; 2013;62: 165–170.

349. Porto JP, Santos RO, Gontijo Filho PP, Ribas RM. Active surveillance to determine the impact of methicillin resistance on mortality in patients with bacteremia and influences of the use of antibiotics on the development of MRSA infection. Rev Soc Bras Med Trop. Brazil; 2013;46: 713–718. doi:10.1590/0037-8682-0199-2013

350. Gonzalez L, Cravoisy A, Barraud D, Conrad M, Nace L, Lemarie J, et al. Factors influencing the implementation of antibiotic de-escalation and impact of this strategy in critically ill patients. Crit Care. England; 2013;17: R140. doi:10.1186/cc12819

351. Gurieva T, Bootsma MCJ, Bonten MJM. Cost and effects of different admission screening strategies to control the spread of methicillin-resistant Staphylococcus aureus. PLoS Comput Biol. United States; 2013;9: e1002874. doi:10.1371/journal.pcbi.1002874

352. Caini S, Hajdu A, Kurcz A, Borocz K. Hospital-acquired infections due to multidrug-resistant organisms in Hungary, 2005-2010. Euro Surveill Bull Eur sur les Mal Transm = Eur Commun Dis Bull. Sweden; 2013;18.

353. Tsai H-Y, Lauderdale T-L, Wang J-T, Chen Y-S, Liu J-W, Huang J-H, et al. Updated antibiotic resistance and clinical spectrum of infections caused by Streptococcus pneumoniae in Taiwan: Emphasis on risk factors for penicillin nonsusceptibilities. J Microbiol Immunol Infect. England; 2013;46: 345–351. doi:10.1016/j.jmii.2012.07.012

354. Apostolopoulou E, Raftopoulos V, Filntisis G, Kithreotis P, Stefanidis E, Galanis P, et al. Surveillance of device-associated infection rates and mortality in 3 Greek intensive care units. Am J Crit Care. United States; 2013;22: e12-20. doi:10.4037/ajcc2013324

355. Fukuta Y, Muder RR, Agha ME, Clarke LG, Wagener MM, Hensler AM, et al. Risk factors for acquisition of multidrug-resistant Acinetobacter baumannii among cancer patients. Am J Infect Control. United States; 2013;41: 1249–1252. doi:10.1016/j.ajic.2013.04.003

356. Li Y, Xu Y, Xu J, Liu X, Li Y. [Impact factors analysis of standardized perioperative antibiotic application on nosocomial infection and drug-resistance bacteria]. Zhonghua Yan Ke Za Zhi. China; 2013;49: 1002–1005.

357. Garcia-Vazquez E, Albendin H, Hernandez-Torres A, Canteras M, Yague G, Ruiz J, et al. [Study of a cohort of patients with Enterococcus spp. Bacteraemia. Risk factors associated to high-level resistance to aminoglycosides]. Rev Esp Quimioter. Spain; 2013;26: 203–213.

358. Cardoso T, Ribeiro O, Aragao I, Costa-Pereira A, Sarmento A. Differences in microbiological profile between community-acquired, healthcare-associated and hospital-acquired infections. Acta Med Port. Portugal; 2013;26: 377–384.

359. Hobday RA, Dancer SJ. Roles of sunlight and natural ventilation for controlling infection: historical and current perspectives. J Hosp Infect. England; 2013;84: 271–282. doi:10.1016/j.jhin.2013.04.011

360. Karvouniaris M, Makris D, Manoulakas E, Zygoulis P, Mantzarlis K, Triantaris A, et al. Ventilator-associated tracheobronchitis increases the length of intensive care unit stay. Infect Control Hosp Epidemiol. United States; 2013;34: 800–808. doi:10.1086/671274

361. Salgado CD, Sepkowitz KA, John JF, Cantey JR, Attaway HH, Freeman KD, et al. Copper surfaces reduce the rate of healthcare-acquired infections in the intensive care unit. Infect Control Hosp Epidemiol. United States; 2013;34: 479–486. doi:10.1086/670207

362. Rothe C, Schlaich C, Thompson S. Healthcare-associated infections in sub-Saharan Africa. J Hosp Infect. England; 2013;85: 257–267. doi:10.1016/j.jhin.2013.09.008

363. Nagao M. A multicentre analysis of epidemiology of the nosocomial bloodstream infections in Japanese university hospitals. Clin Microbiol Infect. England; 2013;19: 852–858. doi:10.1111/1469-0691.12083

364. Li F, Wu L, Cao B, Zhang Y, Li X, Liu Y. Surveillance of the prevalence, antibiotic susceptibility, and genotypic characterization of invasive candidiasis in a teaching hospital in China between 2006 to 2011. BMC Infect Dis. England; 2013;13: 353. doi:10.1186/1471-2334-13-353

365. Petti S, Polimeni A, Dancer SJ. Effect of disposable barriers, disinfection, and cleaning on controlling methicillin-resistant Staphylococcus aureus environmental contamination. Am J Infect Control. United States; 2013;41: 836–840. doi:10.1016/j.ajic.2012.09.021

366. Kapoor K, Jajoo M, Dublish S, Dabas V, Gupta S, Manchanda V. Intravenous colistin for multidrug-resistant gram-negative infections in critically ill pediatric patients. Pediatr Crit Care Med. United States; 2013;14: e268-72. doi:10.1097/PCC.0b013e31828a740f

367. Resende MM, Monteiro SG, Callegari B, Figueiredo PMS, Monteiro CRA V, Monteiro-Neto V. Epidemiology and outcomes of ventilator-associated pneumonia in northern Brazil: an analytical descriptive prospective cohort study. BMC Infect Dis. England; 2013;13: 119. doi:10.1186/1471-2334-13-119

368. Nair GB, Niederman MS. Nosocomial pneumonia: lessons learned. Crit Care Clin. United States; 2013;29: 521–546. doi:10.1016/j.ccc.2013.03.007

369. Afonso E, Llaurado M, Gallart E. The value of chlorhexidine gluconate wipes and prepacked washcloths to prevent the spread of pathogens--a systematic review. Aust Crit Care. Australia; 2013;26: 158–166. doi:10.1016/j.aucc.2013.05.001

370. Lopez-Cerero L, Fernandez-Cuenca F, Pascual A. [The Microbiolgy laboratory in nosocomial infection surveillance and control]. Enferm Infecc Microbiol Clin. Spain; 2013;31: 44–51. doi:10.1016/j.eimc.2012.10.001

371. Liao C-H, Hsueh P-R, Jacoby GA, Hooper DC. Risk factors and clinical characteristics of patients with qnr-positive Klebsiella pneumoniae bacteraemia. J Antimicrob Chemother. England; 2013;68: 2907–2914. doi:10.1093/jac/dkt295

372. Fernandez-Hidalgo N, Almirante B, Gavalda J, Gurgui M, Pena C, de Alarcon A, et al. Ampicillin plus ceftriaxone is as effective as ampicillin plus gentamicin for treating enterococcus faecalis infective endocarditis. Clin Infect Dis. United States; 2013;56: 1261–1268. doi:10.1093/cid/cit052

373. Su J, Wang X, Chen J, Wu G, Jin Y, Xu F, et al. Glycemic variability in gestational diabetes mellitus and its association with beta cell function. Endocrine. United States; 2013;43: 370–375. doi:10.1007/s12020-012-9753-5

374. Dancer SJ. Controlling hospital-acquired infection: focus on the role of the environment and new technologies for decontamination. Clin Microbiol Rev. United States; 2014;27: 665–690. doi:10.1128/CMR.00020-14

375. Kim C-J, Kim H-B, Oh M, Kim Y, Kim A, Oh S-H, et al. The burden of nosocomial staphylococcus aureus bloodstream infection in South Korea: a prospective hospital-based nationwide study. BMC Infect Dis. England; 2014;14: 590. doi:10.1186/s12879-014-0590-4

376. Tsai M-H, Chu S-M, Hsu J-F, Lien R, Huang H-R, Chiang M-C, et al. Risk factors and outcomes for multidrug-resistant Gram-negative bacteremia in the NICU. Pediatrics. United States; 2014;133: e322-9. doi:10.1542/peds.2013-1248

377. Chusri S, Chongsuvivatwong V, Rivera JI, Silpapojakul K, Singkhamanan K, McNeil E, et al. Clinical outcomes of hospital-acquired infection with Acinetobacter nosocomialis and Acinetobacter pittii. Antimicrob Agents Chemother. United States; 2014;58: 4172–4179. doi:10.1128/AAC.02992-14

378. Widner A, Nobles DL, Faulk C, Vos P, Ramsey KM. The impact of a “search and destroy” strategy for the prevention of methicillin-resistant Staphylococcus aureus infections in an inpatient rehabilitation facility. PM R. United States; 2014;6: 121–6; quiz 126. doi:10.1016/j.pmrj.2013.09.013

379. Pradhan NP, Bhat SM, Ghadage DP. Nosocomial infections in the medical ICU: a retrospective study highlighting their prevalence, microbiological profile and impact on ICU stay and mortality. J Assoc Physicians India. India; 2014;62: 18–21.

380. Willemsen I, Nelson-Melching J, Hendriks Y, Mulders A, Verhoeff S, Kluytmans-Vandenbergh M, et al. Measuring the quality of infection control in Dutch nursing homes using a standardized method; the Infection prevention RIsk Scan (IRIS). Antimicrob Resist Infect Control. England; 2014;3: 26. doi:10.1186/2047-2994-3-26

381. Cobos-Trigueros N, Rinaudo M, Sole M, Castro P, Pumarol J, Hernandez C, et al. Acquisition of resistant microorganisms and infections in HIV-infected patients admitted to the ICU. Eur J Clin Microbiol Infect Dis. Germany; 2014;33: 611–620. doi:10.1007/s10096-013-1995-5

382. Patel SJ, Oliveira AP, Zhou JJ, Alba L, Furuya EY, Weisenberg SA, et al. Risk factors and outcomes of infections caused by extremely drug-resistant gram-negative bacilli in patients hospitalized in intensive care units. Am J Infect Control. United States; 2014;42: 626–631. doi:10.1016/j.ajic.2014.01.027

383. Moreno Camacho A, Ruiz Camps I. [Nosocomial infection in patients receiving a solid organ transplant or haematopoietic stem cell transplant]. Enferm Infecc Microbiol Clin. Spain; 2014;32: 386–395. doi:10.1016/j.eimc.2014.05.002

384. Camus C, Salomon S, Bouchigny C, Gacouin A, Lavoue S, Donnio P-Y, et al. Short-term decline in all-cause acquired infections with the routine use of a decontamination regimen combining topical polymyxin, tobramycin, and amphotericin B with mupirocin and chlorhexidine in the ICU: a single-center experience. Crit Care Med. United States; 2014;42: 1121–1130. doi:10.1097/CCM.0000000000000140

385. Oncul O, Oksuz S, Acar A, Ulkur E, Turhan V, Uygur F, et al. Nosocomial infection characteristics in a burn intensive care unit: analysis of an eleven-year active surveillance. Burns. Netherlands; 2014;40: 835–841. doi:10.1016/j.burns.2013.11.003

386. Nicholson L. Healthcare-associated infections: the value of patient isolation. Nurs Stand. England; 2014;29: 35–44. doi:10.7748/ns.29.6.35.e8789

387. Mitt P, Metsvaht T, Adamson V, Telling K, Naaber P, Lutsar I, et al. Five-year prospective surveillance of nosocomial bloodstream infections in an Estonian paediatric intensive care unit. J Hosp Infect. England; 2014;86: 95–99. doi:10.1016/j.jhin.2013.11.002

388. Mann EE, Manna D, Mettetal MR, May RM, Dannemiller EM, Chung KK, et al. Surface micropattern limits bacterial contamination. Antimicrob Resist Infect Control. England; 2014;3: 28. doi:10.1186/2047-2994-3-28

389. Yan L-H, Wei W-Y, Xie Y-B, Xiao Q. New insights into the functions and localization of the homeotic gene CDX2 in gastric cancer. World J Gastroenterol. United States; 2014;20: 3960–3966. doi:10.3748/wjg.v20.i14.3960

390. Pegues DA. Translating and scaling the HHS Action Plan to Prevent Healthcare-associated Infections to the local level: experience of a Los Angeles Health System. Med Care. United States; 2014;52: S60-5. doi:10.1097/MLR.0000000000000054

391. Righi E, Aggazzotti G, Ferrari E, Giovanardi C, Busani S, Rinaldi L, et al. Trends in ventilator-associated pneumonia: impact of a ventilator care bundle in an Italian tertiary care hospital intensive care unit. Am J Infect Control. United States; 2014;42: 1312–1316. doi:10.1016/j.ajic.2014.08.009

392. Charles MP, Kali A, Easow JM, Joseph NM, Ravishankar M, Srinivasan S, et al. Ventilator-associated pneumonia. Australas Med J. Canada; 2014;7: 334–344. doi:10.4066/AMJ.2014.2105

393. Iakovlev S V, Suvorova MP, Kolendo SE, Burmistrova EN, Sergeeva E V, Cherkasova NA, et al. [Clinical efficacy of the antimicrobial drug furamag in nosocomial urinary tract infections]. Ter Arkh. Russia (Federation); 2014;86: 65–72.

394. Zhao H, Yang C, Guo Y, Wang H, Wu Y, An Y. [Monitoring and control of pan-drug resistant Acinetobacter baumannii colonization and infection in a medical intensive care unit]. Zhonghua Wei Zhong Bing Ji Jiu Yi Xue. China; 2014;26: 464–467. doi:10.3760/cma.j.issn.2095-4352.2014.07.004

395. Liu YM, Li B Bin, Zhang YY, Zhang W, Shen H, Li H, et al. Clinical and molecular characteristics of emerging hypervirulent Klebsiella pneumoniae bloodstream infections in mainland China. Antimicrob Agents Chemother. United States; 2014;58: 5379–5385. doi:10.1128/AAC.02523-14

396. Edmond MB, Masroor N, Stevens MP, Ober J, Bearman G. The Impact of Discontinuing Contact Precautions for VRE and MRSA on Device-Associated Infections. Infect Control Hosp Epidemiol. United States; 2015;36: 978–980. doi:10.1017/ice.2015.99

397. Sargenti K, Prytz H, Strand A, Nilsson E, Kalaitzakis E. Healthcare-associated and nosocomial bacterial infections in cirrhosis: predictors and impact on outcome. Liver Int. United States; 2015;35: 391–400. doi:10.1111/liv.12625

398. Ahmed NH, Hussain T, Biswal I. Antimicrobial resistance of bacterial isolates from respiratory secretions of ventilated patients in a multi-specialty hospital. Avicenna J Med. India; 2015;5: 74–78. doi:10.4103/2231-0770.160233

399. Cailes B, Vergnano S, Kortsalioudaki C, Heath P, Sharland M. The current and future roles of neonatal infection surveillance programmes in combating antimicrobial resistance. Early Hum Dev. Ireland; 2015;91: 613–618. doi:10.1016/j.earlhumdev.2015.08.012

400. Chen C-H, Lin L-C, Chang Y-J, Liu C-E, Soon M-S. Long-term effectiveness of infection and antibiotic control programs on the transmission of carbapenem-resistant Acinetobacter calcoaceticus-Acinetobacter baumannii complex in central Taiwan. Med Mal Infect. France; 2015;45: 264–272. doi:10.1016/j.medmal.2015.04.005

401. Xu W, He L, Liu C, Rong J, Shi Y, Song W, et al. The Effect of Infection Control Nurses on the Occurrence of Pseudomonas aeruginosa Healthcare-Acquired Infection and Multidrug-Resistant Strains in Critically-Ill Children. PLoS One. United States; 2015;10: e0143692. doi:10.1371/journal.pone.0143692

402. Saravu K, Prasad M, Eshwara VK, Mukhopadhyay C. Clinico-microbiological profile and outcomes of nosocomial sepsis in an Indian tertiary care hospital--a prospective cohort study. Pathog Glob Health. England; 2015;109: 228–235. doi:10.1179/2047773215Y.0000000026

403. El-Mahallawy HA, Mohsen LM, Wassef M. Milestones along the road of infection prevention in Egypt. Eur J Clin Microbiol Infect Dis. Germany; 2015;34: 1923–1928. doi:10.1007/s10096-015-2444-4

404. Gao F, Wu Y-Y, Zou J-N, Zhu M, Zhang J, Huang H-Y, et al. Impact of a bundle on prevention and control of healthcare associated infections in intensive care unit. J Huazhong Univ Sci Technol Med Sci = Hua zhong ke ji da xue xue bao Yi xue Ying wen ban = Huazhong keji daxue xuebao Yixue Yingdewen ban. China; 2015;35: 283–290. doi:10.1007/s11596-015-1425-2

405. Yesilbag Z, Cagatay AA, Karadeniz A, Basaran S, Orhun G, Ergin Ozcan P, et al. [Is there a relationship between rectal colonization and nosocomial infection of patients in intensive care unit?]. Mikrobiyol Bul. Turkey; 2015;49: 327–339.

406. McMaster J, Booth MG, Smith A, Hamilton K. Meticillin-resistant Staphylococcus aureus in the intensive care unit: its effect on outcome and risk factors for acquisition. J Hosp Infect. England; 2015;90: 327–332. doi:10.1016/j.jhin.2015.04.009

407. Nelson RE, Samore MH, Jones M, Greene T, Stevens VW, Liu C-F, et al. Reducing Time-dependent Bias in Estimates of the Attributable Cost of Health Care-associated Methicillin-resistant Staphylococcus aureus Infections: A Comparison of Three Estimation Strategies. Med Care. United States; 2015;53: 827–834. doi:10.1097/MLR.0000000000000403

408. Buick S, Joffe AM, Taylor G, Conly J. A consensus development conference model for establishing health policy for surveillance and screening of antimicrobial-resistant organisms. Clin Infect Dis. United States; 2015;60: 1095–1101. doi:10.1093/cid/ciu1168

409. Brett M, Bradfute SB. Staphylococcus aureus: Current State of Prevalence, Impact, and Vaccine Development. Curr Pharm Des. United Arab Emirates; 2015;21: 2131–2135. doi:10.2174/1381612821666150310101347

410. Slayton RB, Toth D, Lee BY, Tanner W, Bartsch SM, Khader K, et al. Vital Signs: Estimated Effects of a Coordinated Approach for Action to Reduce Antibiotic-Resistant Infections in Health Care Facilities - United States. MMWR Morb Mortal Wkly Rep. United States; 2015;64: 826–831.

411. Bouscambert M, Valette M, Lina B. Rapid bedside tests for diagnosis, management, and prevention of nosocomial influenza. J Hosp Infect. England; 2015;89: 314–318. doi:10.1016/j.jhin.2014.12.017

412. Dabar G, Harmouche C, Salameh P, Jaber BL, Jamaleddine G, Waked M, et al. Community- and healthcare-associated infections in critically ill patients: a multicenter cohort study. Int J Infect Dis. Canada; 2015;37: 80–85. doi:10.1016/j.ijid.2015.05.024

413. Vergara-Lopez S, Dominguez MC, Conejo MC, Pascual A, Rodriguez-Bano J. Lessons from an outbreak of metallo-beta-lactamase-producing Klebsiella oxytoca in an intensive care unit: the importance of time at risk and combination therapy. J Hosp Infect. England; 2015;89: 123–131. doi:10.1016/j.jhin.2013.12.008

414. Cross AS, Greenberg N, Billington M, Zhang L, DeFilippi C, May RC, et al. Phase 1 testing of detoxified LPS/group B meningococcal outer membrane protein vaccine with and without synthetic CPG 7909 adjuvant for the prevention and treatment of sepsis. Vaccine. Netherlands; 2015;33: 6719–6726. doi:10.1016/j.vaccine.2015.10.072

415. Negi V, Pal S, Juyal D, Sharma MK, Sharma N. Bacteriological Profile of Surgical Site Infections and Their Antibiogram: A Study From Resource Constrained Rural Setting of Uttarakhand State, India. J Clin Diagn Res. India; 2015;9: DC17-20. doi:10.7860/JCDR/2015/15342.6698

416. Ye D, Shan J, Huang Y, Li J, Li C, Liu X, et al. A gloves-associated outbreak of imipenem-resistant Acinetobacter baumannii in an intensive care unit in Guangdong, China. BMC Infect Dis. England; 2015;15: 179. doi:10.1186/s12879-015-0917-9

417. Thi Anh Thu L, Thi Hong Thoa V, Thi Van Trang D, Phuc Tien N, Thuy Van D, Thi Kim Anh L, et al. Cost-effectiveness of a hand hygiene program on health care-associated infections in intensive care patients at a tertiary care hospital in Vietnam. Am J Infect Control. United States; 2015;43: e93-9. doi:10.1016/j.ajic.2015.08.006

418. Zusman S, Axel A, Touderis L, Segal E, Schlaeffer F, Schlaeffer P. [The computerized patient record as a tool to improve monitoring and prevention of hospital acquired infections]. Harefuah. Israel; 2015;154: 233-235,280,281.

419. Baratz MD, Hallmark R, Odum SM, Springer BD. Twenty Percent of Patients May Remain Colonized With Methicillin-resistant Staphylococcus aureus Despite a Decolonization Protocol in Patients Undergoing Elective Total Joint Arthroplasty. Clin Orthop Relat Res. United States; 2015;473: 2283–2290. doi:10.1007/s11999-015-4191-3

420. Spencer M, Barnes S, Parada J, Brown S, Perri L, Uettwiller-Geiger D, et al. A primer on on-demand polymerase chain reaction technology. Am J Infect Control. United States; 2015;43: 1102–1108. doi:10.1016/j.ajic.2015.06.001

421. Oz E, Salturk C, Karakurt Z, Yazicioglu Mocin O, Adiguzel N, Gungor G, et al. Risk factors for multiorgan failure and mortality in severe sepsis patients who need intensive care unit follow-up. Tuberk Toraks. Turkey; 2015;63: 147–157.

422. Aycan IO, Celen MK, Yilmaz A, Almaz MS, Dal T, Celik Y, et al. Bacterial colonization due to increased nurse workload in an intensive care unit. Brazilian J Anesthesiol. Brazil; 2015;65: 180–185. doi:10.1016/j.bjane.2014.05.005

423. Boccia S, Pasquarella C, Colotto M, Barchitta M, Quattrocchi A, Agodi A. Molecular epidemiology tools in the management of healthcare-associated infections: towards the definition of recommendations. Epidemiol Prev. Italy; 2015;39: 21–26.

424. Ou X, Xia H, Li Q, Pang Y, Wang S, Zhao B, et al. A feasibility study of the Xpert MTB/RIF test at the peripheral level laboratory in China. Int J Infect Dis. Canada; 2015;31: 41–46. doi:10.1016/j.ijid.2014.09.011

425. Jang JY, Lee SH, Shim H, Choi JY, Yong D, Lee JG. Epidemiology and Microbiology of Secondary Peritonitis Caused by Viscus Perforation: A Single-Center Retrospective Study. Surg Infect (Larchmt). United States; 2015;16: 436–442. doi:10.1089/sur.2014.148

426. Kwee F, Walker SAN, Elligsen M, Palmay L, Simor A, Daneman N. Outcomes in Documented Pseudomonas aeruginosa Bacteremia Treated with Intermittent IV Infusion of Ceftazidime, Meropenem, or Piperacillin-Tazobactam: A Retrospective Study. Can J Hosp Pharm. Canada; 2015;68: 386–394. doi:10.4212/cjhp.v68i5.1485

427. Taylor JE, Tan K, Lai NM, McDonald SJ. Antibiotic lock for the prevention of catheter-related infection in neonates. Cochrane database Syst Rev. England; 2015; CD010336. doi:10.1002/14651858.CD010336.pub2

428. Hamouda K, Oezkur M, Sinha B, Hain J, Menkel H, Leistner M, et al. Different duration strategies of perioperative antibiotic prophylaxis in adult patients undergoing cardiac surgery: an observational study. J Cardiothorac Surg. England; 2015;10: 25. doi:10.1186/s13019-015-0225-x

429. Aamodt H, Mohn SC, Maselle S, Manji KP, Willems R, Jureen R, et al. Genetic relatedness and risk factor analysis of ampicillin-resistant and high-level gentamicin-resistant enterococci causing bloodstream infections in Tanzanian children. BMC Infect Dis. England; 2015;15: 107. doi:10.1186/s12879-015-0845-8

430. Dananche C, Benet T, Allaouchiche B, Hernu R, Argaud L, Dauwalder O, et al. Targeted screening for third-generation cephalosporin-resistant Enterobacteriaceae carriage among patients admitted to intensive care units: a quasi-experimental study. Crit Care. England; 2015;19: 38. doi:10.1186/s13054-015-0754-7

431. Nair GB, Niederman MS. Ventilator-associated pneumonia: present understanding and ongoing debates. Intensive Care Med. United States; 2015;41: 34–48. doi:10.1007/s00134-014-3564-5

432. Yosipovich R, Aizenshtein E, Shadmon R, Krispel S, Shuster E, Pitcovski J. Overcoming the susceptibility gap between maternal antibody disappearance and auto-antibody production. Vaccine. Netherlands; 2015;33: 472–478. doi:10.1016/j.vaccine.2014.10.043

433. Lim C, Takahashi E, Hongsuwan M, Wuthiekanun V, Thamlikitkul V, Hinjoy S, et al. Epidemiology and burden of multidrug-resistant bacterial infection in a developing country. Elife. England; 2016;5. doi:10.7554/eLife.18082

434. Frost SA, Alogso M-C, Metcalfe L, Lynch JM, Hunt L, Sanghavi R, et al. Chlorhexidine bathing and health care-associated infections among adult intensive care patients: a systematic review and meta-analysis. Crit Care. England; 2016;20: 379. doi:10.1186/s13054-016-1553-5

435. Rosenthal VD. International Nosocomial Infection Control Consortium (INICC) resources: INICC multidimensional approach and INICC surveillance online system. Am J Infect Control. United States; 2016;44: e81-90. doi:10.1016/j.ajic.2016.01.005

436. Nelson RE, Schweizer ML, Perencevich EN, Nelson SD, Khader K, Chiang H-Y, et al. Costs and Mortality Associated With Multidrug-Resistant Healthcare-Associated Acinetobacter Infections. Infect Control Hosp Epidemiol. United States; 2016;37: 1212–1218. doi:10.1017/ice.2016.145

437. Micek ST, Chew B, Hampton N, Kollef MH. A Case-Control Study Assessing the Impact of Nonventilated Hospital-Acquired Pneumonia on Patient Outcomes. Chest. United States; 2016;150: 1008–1014. doi:10.1016/j.chest.2016.04.009

438. Iliyasu G, Daiyab FM, Tiamiyu AB, Abubakar S, Habib ZG, Sarki AM, et al. Nosocomial infections and resistance pattern of common bacterial isolates in an intensive care unit of a tertiary hospital in Nigeria: A 4-year review. J Crit Care. United States; 2016;34: 116–120. doi:10.1016/j.jcrc.2016.04.018

439. Dramowski A, Whitelaw A, Cotton MF. Burden, spectrum, and impact of healthcare-associated infection at a South African children’s hospital. J Hosp Infect. England; 2016;94: 364–372. doi:10.1016/j.jhin.2016.08.022

440. Al-Mousa HH, Omar AA, Rosenthal VD, Salama MF, Aly NY, El-Dossoky Noweir M, et al. Device-associated infection rates, bacterial resistance, length of stay, and mortality in Kuwait: International Nosocomial Infection Consortium findings. Am J Infect Control. United States; 2016;44: 444–449. doi:10.1016/j.ajic.2015.10.031

441. Folgori L, Bernaschi P, Piga S, Carletti M, Cunha FP, Lara PHR, et al. Healthcare-Associated Infections in Pediatric and Neonatal Intensive Care Units: Impact of Underlying Risk Factors and Antimicrobial Resistance on 30-Day Case-Fatality in Italy and Brazil. Infect Control Hosp Epidemiol. United States; 2016;37: 1302–1309. doi:10.1017/ice.2016.185

442. Girerd-Genessay I, Benet T, Vanhems P. Multidrug-Resistant Bacterial Outbreaks in Burn Units: A Synthesis of the Literature According to the ORION Statement. J Burn Care Res. England; 2016;37: 172–180. doi:10.1097/BCR.0000000000000256

443. Babady NE. Hospital-Associated Infections. Microbiol Spectr. United States; 2016;4. doi:10.1128/microbiolspec.DMIH2-0003-2015

444. De Vos D, Pirnay J-P, Bilocq F, Jennes S, Verbeken G, Rose T, et al. Molecular Epidemiology and Clinical Impact of Acinetobacter calcoaceticus-baumannii Complex in a Belgian Burn Wound Center. PLoS One. United States; 2016;11: e0156237. doi:10.1371/journal.pone.0156237

445. Wang J, Gao P, Guo S, Liu Y, Shao L, Kang H, et al. [Analysis of death risk factors for nosocomial infection patients in an ICU: a retrospective review of 864 patients from 2009 to 2015]. Zhonghua Wei Zhong Bing Ji Jiu Yi Xue. China; 2016;28: 704–708. doi:10.3760/cma.j.issn.2095-4352.2016.08.007

446. No Title. Geneva; 2016.

447. Sonmezer MC, Ertem G, Erdinc FS, Kaya Kilic E, Tulek N, Adiloglu A, et al. Evaluation of Risk Factors for Antibiotic Resistance in Patients with Nosocomial Infections Caused by Pseudomonas aeruginosa. Can J Infect Dis Med Microbiol = J Can des Mal Infect la Microbiol medicale. Egypt; 2016;2016: 1321487. doi:10.1155/2016/1321487

448. Mendes ET, Ranzani OT, Marchi AP, Silva MT da, Filho JUA, Alves T, et al. Chlorhexidine bathing for the prevention of colonization and infection with multidrug-resistant microorganisms in a hematopoietic stem cell transplantation unit over a 9-year period: Impact on chlorhexidine susceptibility. Medicine (Baltimore). United States; 2016;95: e5271. doi:10.1097/MD.0000000000005271

449. Fitzpatrick F, Riordan MO. Performance management of Clostridium difficile infection in hospitals - The carrot or stick approach? Anaerobe. England; 2016;37: 8–12. doi:10.1016/j.anaerobe.2015.10.001

450. Deng C, Zhang W, Yuan Y, Yao A, Hu Y, Yu F, et al. Report: Prevention infection of newborn nosocomial and distribution of multiple drug resistant organism of the medicinal. Pak J Pharm Sci. Pakistan; 2016;29: 361–365.

451. Cartelle Gestal M, Zurita J, Gualpa G, Gonzalez C, Paz Y Mino A. Early detection and control of an Acinetobacter baumannii multi-resistant outbreak in a hospital in Quito, Ecuador. J Infect Dev Ctries. Italy; 2016;10: 1294–1298. doi:10.3855/jidc.7544

452. Park C-K, Oh H-J, Choi H-Y, Shin H-J, Lim JH, Oh I-J, et al. Microbiological Characteristics and Predictive Factors for Mortality in Pleural Infection: A Single-Center Cohort Study in Korea. PLoS One. United States; 2016;11: e0161280. doi:10.1371/journal.pone.0161280

453. Kim T, Hong SI, Park SY, Jung J, Chong YP, Kim S-H, et al. Clinical Features and Outcomes of Spontaneous Bacterial Peritonitis Caused by Streptococcus pneumoniae: A Matched Case-Control Study. Medicine (Baltimore). United States; 2016;95: e3796. doi:10.1097/MD.0000000000003796

454. Benattar YD, Omar M, Zusman O, Yahav D, Zak-Doron Y, Altunin S, et al. The Effectiveness and Safety of High-Dose Colistin: Prospective Cohort Study. Clin Infect Dis. United States; 2016;63: 1605–1612. doi:10.1093/cid/ciw684

455. Ledoux G, Six S, Lawson R, Labreuche J, Blazejewski C, Wallet F, et al. Impact of a targeted isolation strategy at intensive-care-unit-admission on intensive-care-unit-acquired infection related to multidrug-resistant bacteria: a prospective uncontrolled before-after study. Clin Microbiol Infect. England; 2016;22: 888.e11-888.e18. doi:10.1016/j.cmi.2016.07.012

456. Ider B-E, Baatar O, Rosenthal VD, Khuderchuluun C, Baasanjav B, Donkhim C, et al. Multicenter study of device-associated infection rates in hospitals of Mongolia: Findings of the International Nosocomial Infection Control Consortium (INICC). Am J Infect Control. United States; 2016;44: 327–331. doi:10.1016/j.ajic.2015.10.010

457. Vianna PG, Dale CRJ, Simmons S, Stibich M, Licitra CM. Impact of pulsed xenon ultraviolet light on hospital-acquired infection rates in a community hospital. Am J Infect Control. United States; 2016;44: 299–303. doi:10.1016/j.ajic.2015.10.009

458. Nelson RE, Stevens VW, Khader K, Jones M, Samore MH, Evans ME, et al. Economic Analysis of Veterans Affairs Initiative to Prevent Methicillin-Resistant Staphylococcus aureus Infections. Am J Prev Med. Netherlands; 2016;50: S58–S65. doi:10.1016/j.amepre.2015.10.016

459. Caselli E, D’Accolti M, Vandini A, Lanzoni L, Camerada MT, Coccagna M, et al. Impact of a Probiotic-Based Cleaning Intervention on the Microbiota Ecosystem of the Hospital Surfaces: Focus on the Resistome Remodulation. PLoS One. United States; 2016;11: e0148857. doi:10.1371/journal.pone.0148857

460. Marani A, Napoli C, Berdini S, Montesano M, Ferretti F, Di Ninno F, et al. Point prevalence surveys on healthcare acquired infections in medical and surgical wards of a teaching hospital in Rome. Ann Ig. Italy; 2016;28: 274–281. doi:10.7416/ai.2016.2106

461. Gallardo-Garcia MM, Sanchez-Espin G, Ivanova-Georgieva R, Ruiz-Morales J, Rodriguez-Bailon I, Vinuela Gonzalez V, et al. Relationship between pathogenic, clinical, and virulence factors of Staphylococcus aureus in infective endocarditis versus uncomplicated bacteremia: a case-control study. Eur J Clin Microbiol Infect Dis. Germany; 2016;35: 821–828. doi:10.1007/s10096-016-2603-2

462. Weiner LM, Fridkin SK, Aponte-Torres Z, Avery L, Coffin N, Dudeck MA, et al. Vital Signs: Preventing Antibiotic-Resistant Infections in Hospitals - United States, 2014. MMWR Morb Mortal Wkly Rep. United States; 2016;65: 235–241. doi:10.15585/mmwr.mm6509e1

463. Mitharwal SM, Yaddanapudi S, Bhardwaj N, Gautam V, Biswal M, Yaddanapudi L. Intensive care unit-acquired infections in a tertiary care hospital: An epidemiologic survey and influence on patient outcomes. Am J Infect Control. United States; 2016;44: e113-7. doi:10.1016/j.ajic.2016.01.021

464. Mayer CL, Haley VB, Giardina R, Hazamy PA, Tsivitis M, Knab R, et al. Lessons learned from initial reporting of carbapenem-resistant Enterobacteriaceae in New York State hospitals, 2013-2014. Am J Infect Control. United States; 2016;44: 131–133. doi:10.1016/j.ajic.2015.09.001

465. Mataseje LF, Abdesselam K, Vachon J, Mitchel R, Bryce E, Roscoe D, et al. Results from the Canadian Nosocomial Infection Surveillance Program on Carbapenemase-Producing Enterobacteriaceae, 2010 to 2014. Antimicrob Agents Chemother. United States; 2016;60: 6787–6794. doi:10.1128/AAC.01359-16

466. Hashemian M, Talaie H, Akbarpour S, Mahdavinejad A, Mozafari N. Central Nervous System Depressants Poisoning and Ventilator Associated Pneumonia: An Underrated Risk Factor at the Toxicological Intensive Care Unit. Iran Red Crescent Med J. Iran; 2016;18: e30989. doi:10.5812/ircmj.30989

467. Ndir A, Diop A, Faye PM, Cisse MF, Ndoye B, Astagneau P. Epidemiology and Burden of Bloodstream Infections Caused by Extended-Spectrum Beta-Lactamase Producing Enterobacteriaceae in a Pediatric Hospital in Senegal. PLoS One. United States; 2016;11: e0143729. doi:10.1371/journal.pone.0143729

468. Okazaki R, Hagiwara S, Kimura T, Tokue Y, Kambe M, Murata M, et al. Metallo-beta-lactamase-producing Klebsiella pneumoniae infection in a non-hospital environment. Acute medicine & surgery. United States; 2016. pp. 32–35. doi:10.1002/ams2.120

469. Mathai AS, Phillips A, Isaac R. Ventilator-associated pneumonia: A persistent healthcare problem in Indian Intensive Care Units! Lung India. India; 2016;33: 512–516. doi:10.4103/0970-2113.188971

470. Atici S, Soysal A, Kepenekli Kadayifci E, Karaaslan A, Akkoc G, Yakut N, et al. Healthcare-associated infections in a newly opened pediatric intensive care unit in Turkey: Results of four-year surveillance. J Infect Dev Ctries. Italy; 2016;10: 254–259. doi:10.3855/jidc.7517

471. Yang D, Xie Z, Xin X, Xue W, Zhang M. A model for predicting nosocomial carbapenem-resistant Klebsiella pneumoniae infection. Biomed reports. England; 2016;5: 501–505. doi:10.3892/br.2016.752

472. Di Pasquale M, Aliberti S, Mantero M, Bianchini S, Blasi F. Non-Intensive Care Unit Acquired Pneumonia: A New Clinical Entity? Int J Mol Sci. Switzerland; 2016;17: 287. doi:10.3390/ijms17030287

473. Huang SS, Septimus E, Hayden MK, Kleinman K, Sturtevant J, Avery TR, et al. Effect of body surface decolonisation on bacteriuria and candiduria in intensive care units: an analysis of a cluster-randomised trial. Lancet Infect Dis. United States; 2016;16: 70–79. doi:10.1016/S1473-3099(15)00238-8

474. Yagmurdur H, Tezcan AH, Karakurt O, Leblebici F. The efficiency of routine endotracheal aspirate cultures compared to bronchoalveolar lavage cultures in ventilator-associated pneumonia diagnosis. Niger J Clin Pract. India; 2016;19: 46–51. doi:10.4103/1119-3077.164327

475. Jiang Y, Resch S, Liu X, Rogers SOJ, Askari R, Klompas M, et al. The Cost of Responding to an Acinetobacter Outbreak in Critically Ill Surgical Patients. Surg Infect (Larchmt). United States; 2016;17: 58–64. doi:10.1089/sur.2015.036

476. van Duin D, Strassle PD, DiBiase LM, Lachiewicz AM, Rutala WA, Eitas T, et al. Timeline of health care-associated infections and pathogens after burn injuries. Am J Infect Control. United States; 2016;44: 1511–1516. doi:10.1016/j.ajic.2016.07.027

477. Chang K-C, Wu J-F, Hsu H-Y, Chen H-L, Ni Y-H, Chang M-H. Entecavir Treatment in Children and Adolescents with Chronic Hepatitis B Virus Infection. Pediatr Neonatol. Singapore; 2016;57: 390–395. doi:10.1016/j.pedneo.2015.09.009

478. Pan S, Xu J-J, Han X-X, Zhang J, Hu Q-H, Chu Z-X, et al. Internet-Based Sex-Seeking Behavior Promotes HIV Infection Risk: A 6-Year Serial Cross-Sectional Survey to MSM in Shenyang, China. Biomed Res Int. United States; 2016;2016: 2860346. doi:10.1155/2016/2860346

479. Rosenthal VD, Al-Abdely HM, El-Kholy AA, AlKhawaja SAA, Leblebicioglu H, Mehta Y, et al. International Nosocomial Infection Control Consortium report, data summary of 50 countries for 2010-2015: Device-associated module. Am J Infect Control. United States; 2016;44: 1495–1504. doi:10.1016/j.ajic.2016.08.007

480. French Intensive Care Society, International congress - Reanimation 2016. Ann Intensive Care. Germany; 2016;6: 50. doi:10.1186/s13613-016-0114-z

481. Fernando SA, Gray TJ, Gottlieb T. Healthcare-acquired infections: prevention strategies. Intern Med J. Australia; 2017;47: 1341–1351. doi:10.1111/imj.13642

482. Ramasethu J. Prevention and treatment of neonatal nosocomial infections. Matern Heal Neonatol Perinatol. England; 2017;3: 5. doi:10.1186/s40748-017-0043-3

483. Ruiz J, Ramirez P, Villarreal E, Gordon M, Saez I, Rodriguez A, et al. Daily bathing strategies and cross-transmission of multidrug-resistant organisms: Impact of chlorhexidine-impregnated wipes in a multidrug-resistant gram-negative bacteria endemic intensive care unit. Am J Infect Control. United States; 2017;45: 1069–1073. doi:10.1016/j.ajic.2017.06.029

484. Duszynska W, Adamik B, Lentka-Bera K, Kulpa K, Nieckula-Schwarz A, Litwin A, et al. Effect of universal chlorhexidine decolonisation on the infection rate in intensive care patients. Anaesthesiol Intensive Ther. Poland; 2017;49: 28–33. doi:10.5603/AIT.2017.0007

485. Storr J, Twyman A, Zingg W, Damani N, Kilpatrick C, Reilly J, et al. Core components for effective infection prevention and control programmes: new WHO evidence-based recommendations. Antimicrob Resist Infect Control. England; 2017;6: 6. doi:10.1186/s13756-016-0149-9

486. Lowe CF, Lloyd-Smith E, Sidhu B, Ritchie G, Sharma A, Jang W, et al. Reduction in hospital-associated methicillin-resistant Staphylococcus aureus and vancomycin-resistant Enterococcus with daily chlorhexidine gluconate bathing for medical inpatients. Am J Infect Control. United States; 2017;45: 255–259. doi:10.1016/j.ajic.2016.09.019

487. Reynaga E, Torres C, Garcia-Nunez M, Navarro M, Vilamala A, Puigoriol E, et al. Clinical impact and prevalence of MRSA CC398 and differences between MRSA-Tet(R) and MRSA-Tet(S) in an area of Spain with a high density of pig farming: a prospective cohort study. Clin Microbiol Infect. England; 2017;23: 678.e1-678.e4. doi:10.1016/j.cmi.2017.03.019

488. Salgado Yepez E, Bovera MM, Rosenthal VD, Gonzalez Flores HA, Pazmino L, Valencia F, et al. Device-associated infection rates, mortality, length of stay and bacterial resistance in intensive care units in Ecuador: International Nosocomial Infection Control Consortium’s findings. World J Biol Chem. United States; 2017;8: 95–101. doi:10.4331/wjbc.v8.i1.95

489. Sahu MK, Siddharth CB, Devagouru V, Talwar S, Singh SP, Chaudhary S, et al. Hospital-acquired Infection: Prevalence and Outcome in Infants Undergoing Open Heart Surgery in the Present Era. Indian J Crit Care Med. India; 2017;21: 281–286. doi:10.4103/ijccm.IJCCM_62_17

490. Kritsotakis EI, Kontopidou F, Astrinaki E, Roumbelaki M, Ioannidou E, Gikas A. Prevalence, incidence burden, and clinical impact of healthcare-associated infections and antimicrobial resistance: a national prevalent cohort study in acute care hospitals in Greece. Infect Drug Resist. New Zealand; 2017;10: 317–328. doi:10.2147/IDR.S147459

491. No Title. Geneva; 2017.

492. Starzyk-Luszcz K, Zielonka TM, Jakubik J, Zycinska K. Mortality Due to Nosocomial Infection with Klebsiella pneumoniae ESBL<sup/>. Adv Exp Med Biol. United States; 2017;1022: 19–26. doi:10.1007/5584_2017_38

493. Agaba P, Tumukunde J, Tindimwebwa JVB, Kwizera A. Nosocomial bacterial infections and their antimicrobial susceptibility patterns among patients in Ugandan intensive care units: a cross sectional study. BMC Res Notes. England; 2017;10: 349. doi:10.1186/s13104-017-2695-5

494. Kossow A, Kampmeier S, Willems S, Berdel WE, Groll AH, Burckhardt B, et al. Control of Multidrug-Resistant Pseudomonas aeruginosa in Allogeneic Hematopoietic Stem Cell Transplant Recipients by a Novel Bundle Including Remodeling of Sanitary and Water Supply Systems. Clin Infect Dis. United States; 2017;65: 935–942. doi:10.1093/cid/cix465

495. Khan ID, Basu A, Kiran S, Trivedi S, Pandit P, Chattoraj A. Device-Associated Healthcare-Associated Infections (DA-HAI) and the caveat of multiresistance in a multidisciplinary intensive care unit. Med journal, Armed Forces India. India; 2017;73: 222–231. doi:10.1016/j.mjafi.2016.10.008

496. Empaire GD, Guzman Siritt ME, Rosenthal VD, Perez F, Ruiz Y, Diaz C, et al. Multicenter prospective study on device-associated infection rates and bacterial resistance in intensive care units of Venezuela: International Nosocomial Infection Control Consortium (INICC) findings. Int Health. England; 2017;9: 44–49. doi:10.1093/inthealth/ihw049

497. Al-Gethamy MM, Faidah HS, Adetunji HA, Haseeb A, Ashgar SS, Mohanned TK, et al. Risk factors associated with multi-drug-resistant Acinetobacter baumannii nosocomial infections at a tertiary care hospital in Makkah, Saudi Arabia - a matched case-control study. J Int Med Res. England; 2017;45: 1181–1189. doi:10.1177/0300060517706284

498. Caudill L, Lawson B. A unified inter-host and in-host model of antibiotic resistance and infection spread in a hospital ward. J Theor Biol. England; 2017;421: 112–126. doi:10.1016/j.jtbi.2017.03.025

499. Meissner A, Hasenclever D, Brosteanu O, Chaberny IF. EFFECT of daily antiseptic body wash with octenidine on nosocomial primary bacteraemia and nosocomial multidrug-resistant organisms in intensive care units: design of a multicentre, cluster-randomised, double-blind, cross-over study. BMJ Open. England; 2017;7: e016251. doi:10.1136/bmjopen-2017-016251

500. Green C, Pamplin JC, Chafin KN, Murray CK, Yun HC. Pulsed-xenon ultraviolet light disinfection in a burn unit: Impact on environmental bioburden, multidrug-resistant organism acquisition and healthcare associated infections. Burns. Netherlands; 2017;43: 388–396. doi:10.1016/j.burns.2016.08.027

501. Mollers M, Lutgens SP, Schoffelen AF, Schneeberger PM, Suijkerbuijk AWM. Cost of Nosocomial Outbreak Caused by NDM-1-Containing Klebsiella pneumoniae in the Netherlands, October 2015-January 2016. Emerg Infect Dis. United States; 2017;23: 1574–1576. doi:10.3201/eid2309.161710

502. Shek K, Patidar R, Kohja Z, Liu S, Gawaziuk JP, Gawthrop M, et al. Rate of contamination of hospital privacy curtains on a burns and plastic surgery ward: a cross-sectional study. J Hosp Infect. England; 2017;96: 54–58. doi:10.1016/j.jhin.2017.03.012

503. Chen Y-C, Lin C-F, Rehn Y-JF, Chen J-C, Chen P-Y, Chen C-H, et al. Reduced nosocomial infection rate in a neonatal intensive care unit during a 4-year surveillance period. J Chin Med Assoc. Netherlands; 2017;80: 427–431. doi:10.1016/j.jcma.2017.02.006

504. Baldesi O, Bailly S, Ruckly S, Lepape A, L’Heriteau F, Aupee M, et al. ICU-acquired candidaemia in France: Epidemiology and temporal trends, 2004-2013 - A study from the REA-RAISIN network. J Infect. England; 2017;75: 59–67. doi:10.1016/j.jinf.2017.03.011

505. Agarwal R, Mohapatra S, Rath GP, Kapil A. Active Surveillance of Health Care Associated Infections in Neurosurgical Patients. J Clin Diagn Res. India; 2017;11: DC01–DC04. doi:10.7860/JCDR/2017/26681.10146

506. Mazzeffi M, Gammie J, Taylor B, Cardillo S, Haldane-Lutterodt N, Amoroso A, et al. Healthcare-Associated Infections in Cardiac Surgery Patients With Prolonged Intensive Care Unit Stay. Ann Thorac Surg. Netherlands; 2017;103: 1165–1170. doi:10.1016/j.athoracsur.2016.12.041

507. Gagnaire J, Gagneux-Brunon A, Pouvaret A, Grattard F, Carricajo A, Favier H, et al. Carbapenemase-producing Acinetobacter baumannii: An outbreak report with special highlights on economic burden. Med Mal Infect. France; 2017;47: 279–285. doi:10.1016/j.medmal.2017.02.006

508. Yoshimura J, Kinoshita T, Yamakawa K, Matsushima A, Nakamoto N, Hamasaki T, et al. Impact of Gram stain results on initial treatment selection in patients with ventilator-associated pneumonia: a retrospective analysis of two treatment algorithms. Crit Care. England; 2017;21: 156. doi:10.1186/s13054-017-1747-5

509. Hearn P, Miliya T, Seng S, Ngoun C, Day NPJ, Lubell Y, et al. Prospective surveillance of healthcare associated infections in a Cambodian pediatric hospital. Antimicrob Resist Infect Control. England; 2017;6: 16. doi:10.1186/s13756-017-0172-5

510. Osorio J, Barreto J, Samboni CF, Candelo LA, Alvarez LC, Benavidez S, et al. [Risk factors for acute kidney injury in patients treated with polymyxin B experience from 139 cases at a tertiary university hospital in Colombia]. Rev Chilena Infectol. Chile; 2017;34: 7–13. doi:10.4067/S0716-10182017000100001

511. Wu X, Ye Y-Z, Wang C-Q, Wang A-M, He L-Y, Yu H. A case report of hepatic abscesses with soft tissue infection caused by methicillin resistant Staphylococcus aureus in a young child. Medicine (Baltimore). United States; 2017;96: e9260. doi:10.1097/MD.0000000000009260

512. Hamill ME, Reed CR, Fogel SL, Bradburn EH, Powers KA, Love KM, et al. Contact Isolation Precautions in Trauma Patients: An Analysis of Infectious Complications. Surg Infect (Larchmt). United States; 2017;18: 273–281. doi:10.1089/sur.2015.094

513. Goel A, Biewald M, Huprikar S, Schiano T, Im GY. A Real-World Evaluation of Repeat Paracentesis-guided Management of Spontaneous Bacterial Peritonitis. J Clin Gastroenterol. United States; 2017;51: 278–284. doi:10.1097/MCG.0000000000000704

514. Tanguy M, Kouatchet A, Tanguy B, Pichard E, Fanello S, Joly-Guillou M-L. Management of an Acinetobacter baumannii outbreak in an intensive care unit. Med Mal Infect. France; 2017;47: 409–414. doi:10.1016/j.medmal.2017.06.003

515. Martin ET, Haider S, Palleschi M, Eagle S, Crisostomo D V, Haddox P, et al. Bathing hospitalized dependent patients with prepackaged disposable washcloths instead of traditional bath basins: A case-crossover study. Am J Infect Control. United States; 2017;45: 990–994. doi:10.1016/j.ajic.2017.03.023

516. Orkibi H, Azoulay B, Snir S, Regev D. In-session behaviours and adolescents’ self-concept and loneliness: A psychodrama process-outcome study. Clin Psychol Psychother. England; 2017;24: O1455–O1463. doi:10.1002/cpp.2103

517. Kung M-L, Tai M-H, Lin P-Y, Wu D-C, Wu W-J, Yeh B-W, et al. Silver decorated copper oxide (Ag@CuO) nanocomposite enhances ROS-mediated bacterial architecture collapse. Colloids Surf B Biointerfaces. Netherlands; 2017;155: 399–407. doi:10.1016/j.colsurfb.2017.04.041

518. Sachdeva D, Singh D, Loomba P, Kaur A, Tandon M, Bishnoi I. Assessment of surgical risk factors in the development of ventilator-associated pneumonia in neurosurgical intensive care unit patients: Alarming observations. Neurol India. India; 2017;65: 779–784. doi:10.4103/neuroindia.NI_814_16

519. Auda IG, Al-Kadmy IMS, Kareem SM, Lafta AK, A’Affus MHO, Khit IAA, et al. RAPD- and ERIC-Based Typing of Clinical and Environmental Pseudomonas aeruginosa Isolates. J AOAC Int. England; 2017;100: 532–536. doi:10.5740/jaoacint.16-0267

520. Marra AR, Schweizer ML, Edmond MB. No-Touch Disinfection Methods to Decrease Multidrug-Resistant Organism Infections: A Systematic Review and Meta-analysis. Infect Control Hosp Epidemiol. United States; 2018;39: 20–31. doi:10.1017/ice.2017.226

521. Marchi AP, Perdigao Neto L V, Martins RCR, Rizek CF, Camargo CH, Moreno LZ, et al. Vancomycin-resistant enterococci isolates colonizing and infecting haematology patients: clonality, and virulence and resistance profile. J Hosp Infect. England; 2018;99: 346–355. doi:10.1016/j.jhin.2017.10.010

522. Gottesman B-S, Shitrit P, Katzir M, Chowers M. Antibiotic Exposure in the Community and Resistance Patterns of Escherichia coli Community-Acquired Bloodstream Infection. Isr Med Assoc J. Israel; 2018;20: 382–384.

523. Spatenkova V, Bradac O, Fackova D, Bohunova Z, Suchomel P. Low incidence of multidrug-resistant bacteria and nosocomial infection due to a preventive multimodal nosocomial infection control: a 10-year single centre prospective cohort study in neurocritical care. BMC Neurol. England; 2018;18: 23. doi:10.1186/s12883-018-1031-6

524. Bearman G, Abbas S, Masroor N, Sanogo K, Vanhoozer G, Cooper K, et al. Impact of Discontinuing Contact Precautions for Methicillin-Resistant Staphylococcus aureus and Vancomycin-Resistant Enterococcus: An Interrupted Time Series Analysis. Infect Control Hosp Epidemiol. United States; 2018;39: 676–682. doi:10.1017/ice.2018.57

525. Chen C-T, Wang Y-C, Kuo S-C, Shih F-H, Chen T-L, How C-K, et al. Community-acquired bloodstream infections caused by Acinetobacter baumannii: A matched case-control study. J Microbiol Immunol Infect. England; 2018;51: 629–635. doi:10.1016/j.jmii.2017.02.004

526. Puchter L, Chaberny IF, Schwab F, Vonberg R-P, Bange F-C, Ebadi E. Economic burden of nosocomial infections caused by vancomycin-resistant enterococci. Antimicrob Resist Infect Control. England; 2018;7: 1. doi:10.1186/s13756-017-0291-z

527. Wieland K, Chhatwal P, Vonberg R-P. Nosocomial outbreaks caused by Acinetobacter baumannii and Pseudomonas aeruginosa: Results of a systematic review. Am J Infect Control. United States; 2018;46: 643–648. doi:10.1016/j.ajic.2017.12.014

528. Viet Hung N, Hang PT, Rosenthal VD, Thi Anh Thu L, Thi Thu Nguyet L, Quy Chau N, et al. Multicenter Study of Device-Associated Infection Rates, Bacterial Resistance, Length of Stay, and Mortality in Intensive Care Units of 2 Cities of Vietnam: International Nosocomial Infection Control Consortium Findings. J Patient Saf. United States; 2018; doi:10.1097/PTS.0000000000000499

529. Wang Z, Qin R-R, Huang L, Sun L-Y. Risk Factors for Carbapenem-resistant Klebsiella pneumoniae Infection and Mortality of Klebsiella pneumoniae Infection. Chin Med J (Engl). China; 2018;131: 56–62. doi:10.4103/0366-6999.221267

530. Decraene V, Ghebrehewet S, Dardamissis E, Huyton R, Mortimer K, Wilkinson D, et al. An outbreak of multidrug-resistant Pseudomonas aeruginosa in a burns service in the North of England: challenges of infection prevention and control in a complex setting. J Hosp Infect. England; 2018;100: e239–e245. doi:10.1016/j.jhin.2018.07.012

531. Li Y, Cao X, Ge H, Jiang Y, Zhou H, Zheng W. Targeted surveillance of nosocomial infection in intensive care units of 176 hospitals in Jiangsu province, China. J Hosp Infect. England; 2018;99: 36–41. doi:10.1016/j.jhin.2017.10.009

532. Teerawattanapong N, Panich P, Kulpokin D, Na Ranong S, Kongpakwattana K, Saksinanon A, et al. A Systematic Review of the Burden of Multidrug-Resistant Healthcare-Associated Infections Among Intensive Care Unit Patients in Southeast Asia: The Rise of Multidrug-Resistant Acinetobacter baumannii. Infect Control Hosp Epidemiol. United States; 2018;39: 525–533. doi:10.1017/ice.2018.58

533. Ershova K, Savin I, Kurdyumova N, Wong D, Danilov G, Shifrin M, et al. Implementing an infection control and prevention program decreases the incidence of healthcare-associated infections and antibiotic resistance in a Russian neuro-ICU. Antimicrob Resist Infect Control. England; 2018;7: 94. doi:10.1186/s13756-018-0383-4

534. Freedberg DE, Zhou MJ, Cohen ME, Annavajhala MK, Khan S, Moscoso DI, et al. Pathogen colonization of the gastrointestinal microbiome at intensive care unit admission and risk for subsequent death or infection. Intensive Care Med. United States; 2018;44: 1203–1211. doi:10.1007/s00134-018-5268-8

535. Tsao L-H, Hsin C-Y, Liu H-Y, Chuang H-C, Chen L-Y, Lee Y-J. Risk factors for healthcare-associated infection caused by carbapenem-resistant Pseudomonas aeruginosa. J Microbiol Immunol Infect. England; 2018;51: 359–366. doi:10.1016/j.jmii.2017.08.015

536. Hardy K, Sunnucks K, Gil H, Shabir S, Trampari E, Hawkey P, et al. Increased Usage of Antiseptics Is Associated with Reduced Susceptibility in Clinical Isolates of Staphylococcus aureus. MBio. United States; 2018;9. doi:10.1128/mBio.00894-18

537. Kumar S, Sen P, Gaind R, Verma PK, Gupta P, Suri PR, et al. Prospective surveillance of device-associated health care-associated infection in an intensive care unit of a tertiary care hospital in New Delhi, India. Am J Infect Control. United States; 2018;46: 202–206. doi:10.1016/j.ajic.2017.08.037

538. Hernandez A, Yague G, Garcia Vazquez E, Simon M, Moreno Parrado L, Canteras M, et al. [Nosocomial infections caused by multiresistant Pseudomonas aeruginosa (carbapenems included): predictive and prognostic factors. A prospective study (2016-2017))]. Rev Esp Quimioter. Spain; 2018;31: 123–130.

539. Doubravska L, Uvizl R, Gabrhelik T, Klementova O, Kolar M. [Hospital-acquired pneumonia in the light of current recommendations - is there a space for improving patient care?]. Klin Mikrobiol Infekc Lek. Czech Republic; 2018;24: 4–9.

540. Mathur P. Prevention of healthcare-associated infections in low- and middle-income Countries: The “bundle approach”. Indian J Med Microbiol. India; 2018;36: 155–162. doi:10.4103/ijmm.IJMM_18_152

541. Naghshbandi RZ, Haghighat S, Mahdavi M. Passive immunization against methicillin resistant Staphylococcus aureus recombinant PBP2a in sepsis model of mice: Comparable results with antibiotic therapy. Int Immunopharmacol. Netherlands; 2018;56: 186–192. doi:10.1016/j.intimp.2018.01.035

542. Hassan EA, Elsherbiny NM, Abd El-Rehim AS, Soliman AMA, Ahmed AO. Health care-associated infections in pre-transplant liver intensive care unit: Perspectives and challenges. J Infect Public Health. England; 2018;11: 398–404. doi:10.1016/j.jiph.2017.09.006

543. Dunne SS, Ahonen M, Modic M, Crijns FRL, Keinanen-Toivola MM, Meinke R, et al. Specialized cleaning associated with antimicrobial coatings for reduction of hospital-acquired infection: opinion of the COST Action Network AMiCI (CA15114). J Hosp Infect. England; 2018;99: 250–255. doi:10.1016/j.jhin.2018.03.006

544. Lee H, Han SB, Kim JH, Kang S, Durey A. Risk factors of urinary tract infection caused by extended spectrum beta-lactamase-producing Escherichia coli in emergency department. Am J Emerg Med. United States; 2018;36: 1608–1612. doi:10.1016/j.ajem.2018.01.046

545. Kozuka R, Hai H, Motoyama H, Hagihara A, Fujii H, Uchida-Kobayashi S, et al. The presence of multiple NS5A RASs is associated with the outcome of sofosbuvir and ledipasvir therapy in NS5A inhibitor-naive patients with chronic HCV genotype 1b infection in a real-world cohort. J Viral Hepat. England; 2018;25: 535–542. doi:10.1111/jvh.12850

546. Walaszek M, Rozanska A, Bulanda M, Wojkowska-Mach J, Team PSOHI. Epidemiology of healthcare-associated infections in Polish intensive care. A multicenter study based on active surveillance. Biomed Pap Med Fac Univ Palacky Olomouc Czech Repub. Czech Republic; 2018;162: 190–197. doi:10.5507/bp.2018.006

547. Caselli E, Brusaferro S, Coccagna M, Arnoldo L, Berloco F, Antonioli P, et al. Reducing healthcare-associated infections incidence by a probiotic-based sanitation system: A multicentre, prospective, intervention study. PLoS One. United States; 2018;13: e0199616. doi:10.1371/journal.pone.0199616

548. Shallcross LJ, Mentzer A, Rahman S, Cooke GS, Sriskandan S, Noursadeghi M. Cohort study protocol: Bioresource in Adult Infectious Diseases (BioAID). Wellcome open Res. England; 2018;3: 97. doi:10.12688/wellcomeopenres.14690.1

549. Chen I-L, Lee C-H, Ting S-W, Wang LY-C. Prediction of imipenem-resistant microorganisms among the nosocomial critically ill patients with Gram-negative bacilli septicemia: a simple risk score. Infect Drug Resist. New Zealand; 2018;11: 283–293. doi:10.2147/IDR.S157200

550. Kolpa M, Walaszek M, Gniadek A, Wolak Z, Dobros W. Incidence, Microbiological Profile and Risk Factors of Healthcare-Associated Infections in Intensive Care Units: A 10 Year Observation in a Provincial Hospital in Southern Poland. Int J Environ Res Public Health. Switzerland; 2018;15. doi:10.3390/ijerph15010112

551. Walter J, Haller S, Quinten C, Karki T, Zacher B, Eckmanns T, et al. Healthcare-associated pneumonia in acute care hospitals in European Union/European Economic Area countries: an analysis of data from a point prevalence survey, 2011 to 2012. Euro Surveill Bull Eur sur les Mal Transm = Eur Commun Dis Bull. Sweden; 2018;23. doi:10.2807/1560-7917.ES.2018.23.32.1700843

552. Moffa MA, Walsh TL, Tang A, Bremmer DN. Impact of an antimicrobial stewardship program on healthcare-associated Clostridium difficile rates at a community-based teaching hospital. J Infect Prev. England; 2018;19: 191–194. doi:10.1177/1757177418767760

553. Kumari M, Rastogi N, Malhotra R, Mathur P. Clinico-microbiological profile of healthcare associated pneumonia in critically ill patients at level-I trauma centre of India. J Lab Physicians. India; 2018;10: 406–409. doi:10.4103/JLP.JLP_85_18

554. Dayyab FM, Iliyasu G, Aminu A, Habib ZG, Tiamiyu AB, Tambuwal SH, et al. A prospective study of hospital-acquired infections among adults in a tertiary hospital in north-western Nigeria. Trans R Soc Trop Med Hyg. England; 2018;112: 36–42. doi:10.1093/trstmh/try020

555. Steinhaus N, Al-Talib M, Ive P, Boyles T, Bamford C, Davies M-A, et al. The management and outcomes of Staphylococcus aureus bacteraemia at a South African referral hospital: A prospective observational study. Int J Infect Dis. Canada; 2018;73: 78–84. doi:10.1016/j.ijid.2018.06.004

556. Joshi SC, Diwan V, Joshi R, Sharma M, Pathak A, Shah H, et al. “How Can the Patients Remain Safe, If We Are Not Safe and Protected from the Infections”? A Qualitative Exploration among Health-Care Workers about Challenges of Maintaining Hospital Cleanliness in a Resource Limited Tertiary Setting in Rural India. Int J Environ Res Public Health. Switzerland; 2018;15. doi:10.3390/ijerph15091942

557. Le TS, Nguyen TH, Vo HP, Doan VC, Nguyen HL, Tran MT, et al. Protective Effects of Bacteriophages against Aeromonas hydrophila Species Causing Motile Aeromonas Septicemia (MAS) in Striped Catfish. Antibiot (Basel, Switzerland). Switzerland; 2018;7. doi:10.3390/antibiotics7010016

558. Dadar M, Tiwari R, Karthik K, Chakraborty S, Shahali Y, Dhama K. Candida albicans - Biology, molecular characterization, pathogenicity, and advances in diagnosis and control - An update. Microb Pathog. England; 2018;117: 128–138. doi:10.1016/j.micpath.2018.02.028

559. Liu WJ, Zou R, Hu Y, Zhao M, Quan C, Tan S, et al. Clinical, immunological and bacteriological characteristics of H7N9 patients nosocomially co-infected by Acinetobacter Baumannii: a case control study. BMC Infect Dis. England; 2018;18: 664. doi:10.1186/s12879-018-3447-4

560. Walaszek M, Rozanska A, Bulanda M, Wojkowska-Mach J, Team PS of HI. Alarming results of nosocomial bloodstream infections surveillance in Polish intensive care units. Przegl Epidemiol. Poland; 2018;72: 33–44.

561. Edmiston CEJ, Spencer M, Leaper D. Antiseptic Irrigation as an Effective Interventional Strategy for Reducing the Risk of Surgical Site Infections. Surg Infect (Larchmt). United States; 2018;19: 774–780. doi:10.1089/sur.2018.156

562. Li X-K, Zhang S-F, Xu W, Xing B, Lu Q-B, Zhang P-H, et al. Vascular endothelial injury in severe fever with thrombocytopenia syndrome caused by the novel bunyavirus. Virology. United States; 2018;520: 11–20. doi:10.1016/j.virol.2018.05.001

563. Morales Barroso I, Lopez-Cerero L, Navarro MD, Gutierrez-Gutierrez B, Pascual A, Rodriguez-Bano J. Intestinal colonization due to Escherichia coli ST131: risk factors and prevalence. Antimicrob Resist Infect Control. England; 2018;7: 135. doi:10.1186/s13756-018-0427-9

564. Dat VQ, Duong BD, Nhan DT, Hai NH, Anh NTL, Thu HHK, et al. Viral load suppression and acquired HIV drug resistance in adults receiving antiretroviral therapy in Viet Nam: results from a nationally representative survey. West Pacific Surveill response J WPSAR. Philippines; 2018;9: 16–24. doi:10.5365/wpsar.2018.9.1.008

565. Narayanan N, Rai R, Vaidya P, Desai A, Bhowmick T, Weinstein MP. Comparison of linezolid and daptomycin for the treatment of vancomycin-resistant enterococcal bacteremia. Ther Adv Infect Dis. England; 2019;6: 2049936119828964. doi:10.1177/2049936119828964

566. Xie X, Lyu J, Hussain T, Li M. Drug Prevention and Control of Ventilator-Associated Pneumonia. Front Pharmacol. Switzerland; 2019;10: 298. doi:10.3389/fphar.2019.00298

567. Jia H, Li L, Li W, Hou T, Ma H, Yang Y, et al. Impact of Healthcare-Associated Infections on Length of Stay: A Study in 68 Hospitals in China. Biomed Res Int. United States; 2019;2019: 2590563. doi:10.1155/2019/2590563

568. Gao B, Li X, Yang F, Chen W, Zhao Y, Bai G, et al. Molecular Epidemiology and Risk Factors of Ventilator-Associated Pneumonia Infection Caused by Carbapenem-Resistant Enterobacteriaceae. Front Pharmacol. Switzerland; 2019;10: 262. doi:10.3389/fphar.2019.00262

569. Pau MC, Pantaleo A, Tsamesidis I, Hoang H, Tuan Tran A, Hanh Nguyen TL, et al. Clinical impact of the two ART resistance markers, K13 gene mutations and DPC3 in Vietnam. PLoS One. United States; 2019;14: e0214667. doi:10.1371/journal.pone.0214667

570. Caselli E, Arnoldo L, Rognoni C, D’Accolti M, Soffritti I, Lanzoni L, et al. Impact of a probiotic-based hospital sanitation on antimicrobial resistance and HAI-associated antimicrobial consumption and costs: a multicenter study. Infect Drug Resist. New Zealand; 2019;12: 501–510. doi:10.2147/IDR.S194670

571. Edgeworth JD, Batra R, Wulff J, Harrison D. Reductions in methicillin-resistant Staphylococcus aureus,C. difficile infection and intensive care unit acquired bloodstream infection across the United Kingdom following implementation of a national infection control campaign. Clin Infect Dis. United States; 2019; doi:10.1093/cid/ciz720

572. El Mekes A, Zahlane K, Ait Said L, Tadlaoui Ouafi A, Barakate M. The clinical and epidemiological risk factors of infections due to multi-drug resistant bacteria in an adult intensive care unit of University Hospital Center in Marrakesh-Morocco. J Infect Public Health. England; 2019; doi:10.1016/j.jiph.2019.08.012

573. Castro-Lima VAC de, Borges IC, Joelsons D, Sales VVT, Guimaraes T, Ho YL, et al. Impact of human immunodeficiency virus infection on mortality of patients who acquired healthcare associated-infection in critical care unit. Medicine (Baltimore). United States; 2019;98: e15801. doi:10.1097/MD.0000000000015801

574. Callejas-Diaz A, Fernandez-Perez C, Ramos-Martinez A, Munez-Rubio E, Sanchez-Romero I, Vargas Nunez JA. Impact of Pseudomonas aeruginosa bacteraemia in a tertiary hospital: Mortality and prognostic factors. Med Clin (Barc). Spain; 2019;152: 83–89. doi:10.1016/j.medcli.2018.04.020

575. Zingg W, Park BJ, Storr J, Ahmad R, Tarrant C, Castro-Sanchez E, et al. Technology for the prevention of antimicrobial resistance and healthcare-associated infections; 2017 Geneva IPC-Think Tank (Part 2). Antimicrob Resist Infect Control. England; 2019;8: 83. doi:10.1186/s13756-019-0538-y

576. Salmanov A, Vozianov S, Kryzhevsky V, Litus O, Drozdova A, Vlasenko I. Prevalence of healthcare-associated infections and antimicrobial resistance in acute care hospitals in Kyiv, Ukraine. J Hosp Infect. England; 2019;102: 431–437. doi:10.1016/j.jhin.2019.03.008

577. Palacios-Baena ZR, Delgado-Valverde M, Valiente Mendez A, Almirante B, Gomez-Zorrilla S, Borrell N, et al. Impact of De-escalation on Prognosis of Patients With Bacteremia due to Enterobacteriaceae: A Post Hoc Analysis From a Multicenter Prospective Cohort. Clin Infect Dis. United States; 2019;69: 956–962. doi:10.1093/cid/ciy1032

578. Jia H, Li W, Hou T, Ma H, Yang Y, Wu A, et al. The Attributable Direct Medical Cost of Healthcare Associated Infection Caused by Multidrug Resistance Organisms in 68 Hospitals of China. Biomed Res Int. United States; 2019;2019: 7634528. doi:10.1155/2019/7634528

579. Pintado V, Pazos R, Jimenez-Mejias ME, Rodriguez-Guardado A, Diaz-Pollan B, Cabellos C, et al. Staphylococcus aureus meningitis in adults: A comparative cohort study of infections caused by meticillin-resistant and meticillin-susceptible strains. J Hosp Infect. England; 2019;102: 108–115. doi:10.1016/j.jhin.2018.11.008

580. Peters L, Olson L, Khu DTK, Linnros S, Le NK, Hanberger H, et al. Multiple antibiotic resistance as a risk factor for mortality and prolonged hospital stay: A cohort study among neonatal intensive care patients with hospital-acquired infections caused by gram-negative bacteria in Vietnam. PLoS One. United States; 2019;14: e0215666. doi:10.1371/journal.pone.0215666

581. Jaradat RW, Lahlouh AB, Alshogran OY, Aldabbour BA, Balusha AA. Nosocomial Infections among Patients with Intracranial Hemorrhage: A Retrospective Data Analysis of Predictors and Outcomes. Clin Neurol Neurosurg. Netherlands; 2019;182: 158–166. doi:10.1016/j.clineuro.2019.05.016

582. Raka L, Spahija G, Gashi-Gecaj A, Hamza A, Haxhiu E, Rashiti A, et al. Point prevalence survey of healthcare-associated infections and antimicrobial use in Kosovo hospitals. Infect Dis Rep. Italy; 2019;11: 7975. doi:10.4081/idr.2019.7975

583. Tran DM, Larsson M, Olson L, Hoang NTB, Le NK, Khu DTK, et al. High prevalence of colonisation with carbapenem-resistant Enterobacteriaceae among patients admitted to Vietnamese hospitals: Risk factors and burden of disease. J Infect. England; 2019;79: 115–122. doi:10.1016/j.jinf.2019.05.013

584. Huang SS, Septimus E, Kleinman K, Moody J, Hickok J, Heim L, et al. Chlorhexidine versus routine bathing to prevent multidrug-resistant organisms and all-cause bloodstream infections in general medical and surgical units (ABATE Infection trial): a cluster-randomised trial. Lancet (London, England). England; 2019;393: 1205–1215. doi:10.1016/S0140-6736(18)32593-5

585. Mal PB, Sarfaraz S, Herekar F, Ambreen R. Clinical manifestation and outcomes of multi-drug resistant (MDR) Raoultella terrigena infection - A case series at Indus Health Network, Karachi, Pakistan. IDCases. Netherlands; 2019. p. e00628. doi:10.1016/j.idcr.2019.e00628

586. Bonnet V, Dupont H, Glorion S, Aupee M, Kipnis E, Gerard JL, et al. Influence of bacterial resistance on mortality in intensive care units: a registry study from 2000 to 2013 (IICU Study). J Hosp Infect. England; 2019;102: 317–324. doi:10.1016/j.jhin.2019.01.011

587. Dahiya S, Chhillar AK, Sharma N, Choudhary P, Punia A, Balhara M, et al. Candida auris and Nosocomial Infection. Curr Drug Targets. United Arab Emirates; 2019; doi:10.2174/1389450120666190924155631

588. Park J-W, Lee H, Park SY, Kim TH. Epidemiological, clinical, and microbiological characteristics of carbapenemase-producing Enterobacteriaceae bloodstream infection in the Republic of Korea. Antimicrob Resist Infect Control. England; 2019;8: 48. doi:10.1186/s13756-019-0497-3

589. Chen YY, Chen IH, Chen CS, Sun SM. Incidence and mortality of healthcare-associated infections in hospitalized patients with moderate to severe burns. J Crit Care. United States; 2019;54: 185–190. doi:10.1016/j.jcrc.2019.08.024

590. Mularoni A, Martucci G, Douradinha B, Campanella O, Hazen B, Medaglia A, et al. Epidemiology and successful containment of a carbapenem-resistant Enterobacteriaceae outbreak in a Southern Italian Transplant Institute. Transpl Infect Dis. Denmark; 2019;21: e13119. doi:10.1111/tid.13119

591. Xu YL, Hu LM, Xie ZZ, Dong YW, Dong L. [Impact of antimicrobial stewardship program on antimicrobial usage and detection rate of multidrug-resistant gram-negative bacteria]. Zhonghua er ke za zhi = Chinese J Pediatr. China; 2019;57: 553–558. doi:10.3760/cma.j.issn.0578-1310.2019.07.012

592. Baviskar AS, Khatib KI, Rajpal D, Dongare HC. Nosocomial infections in surgical intensive care unit: A retrospective single-center study. Int J Crit Illn Inj Sci. India; 2019;9: 16–20. doi:10.4103/IJCIIS.IJCIIS_57_18

593. Barbato D, Castellani F, Angelozzi A, Isonne C, Baccolini V, Migliara G, et al. Prevalence survey of healthcare-associated infections in a large teaching hospital. Ann Ig. Italy; 2019;31: 423–435. doi:10.7416/ai.2019.2304

594. Bharadwaj S, Ho SK, Khong KC, Seet A, Yeo KC, Chan XY, et al. Eliminating MRSA transmission in a tertiary neonatal unit-A quality improvement initiative. Am J Infect Control. United States; 2019; doi:10.1016/j.ajic.2019.06.001

595. Wu D, Wu C, Zhang S, Zhong Y. Risk Factors of Ventilator-Associated Pneumonia in Critically III Patients. Front Pharmacol. Switzerland; 2019;10: 482. doi:10.3389/fphar.2019.00482

596. Phodha T, Riewpaiboon A, Malathum K, Coyte PC. Excess annual economic burdens from nosocomial infections caused by multi-drug resistant bacteria in Thailand. Expert Rev Pharmacoecon Outcomes Res. England; 2019;19: 305–312. doi:10.1080/14737167.2019.1537123

597. Drohan SE, Levin SA, Grenfell BT, Laxminarayan R. Incentivizing hospital infection control. Proc Natl Acad Sci U S A. United States; 2019;116: 6221–6225. doi:10.1073/pnas.1812231116

598. Boyd DA, Mataseje LF, Pelude L, Mitchell R, Bryce E, Roscoe D, et al. Results from the Canadian Nosocomial Infection Surveillance Program for detection of carbapenemase-producing Acinetobacter spp. in Canadian hospitals, 2010-16. J Antimicrob Chemother. England; 2019;74: 315–320. doi:10.1093/jac/dky416

599. Fang Q, Feng Y, Feng P, Wang X, Zong Z. Nosocomial bloodstream infection and the emerging carbapenem-resistant pathogen Ralstonia insidiosa. BMC Infect Dis. England; 2019;19: 334. doi:10.1186/s12879-019-3985-4

600. Willemsen A, Cobbold R, Gibson J, Wilks K, Lawler S, Reid S. Infection control practices employed within small animal veterinary practices-A systematic review. Zoonoses Public Health. Germany; 2019;66: 439–457. doi:10.1111/zph.12589

601. Wang J, Liu F, Tan JBX, Harbarth S, Pittet D, Zingg W. Implementation of infection prevention and control in acute care hospitals in Mainland China - a systematic review. Antimicrob Resist Infect Control. England; 2019;8: 32. doi:10.1186/s13756-019-0481-y

602. Mitchell R, Taylor G, Rudnick W, Alexandre S, Bush K, Forrester L, et al. Trends in health care-associated infections in acute care hospitals in Canada: an analysis of repeated point-prevalence surveys. CMAJ. Canada; 2019;191: E981–E988. doi:10.1503/cmaj.190361

603. Gao L, Lee JS, Hubner S, Hulke BS, Qu Y, Rieseberg LH. Genetic and phenotypic analyses indicate that resistance to flooding stress is uncoupled from performance in cultivated sunflower. New Phytol. England; 2019;223: 1657–1670. doi:10.1111/nph.15894

604. Liu Z, Gu Y, Li X, Liu Y, Ye Y, Guan S, et al. Identification and Characterization of NDM-1-producing Hypervirulent (Hypermucoviscous) Klebsiella pneumoniae in China. Ann Lab Med. Korea (South); 2019;39: 167–175. doi:10.3343/alm.2019.39.2.167

605. Jeon J, Park J-H, Yong D. Efficacy of bacteriophage treatment against carbapenem-resistant Acinetobacter baumannii in Galleria mellonella larvae and a mouse model of acute pneumonia. BMC Microbiol. England; 2019;19: 70. doi:10.1186/s12866-019-1443-5

606. Carmeli Y, Troillet N, Karchmer AW, Samore MH. Health and economic outcomes of antibiotic resistance in Pseudomonas aeruginosa. Arch Intern Med. 1999; doi:10.1001/archinte.159.10.1127

607. Rubin RJ, Harrington CA, Poon A, Dietrich K, Greene JA, Moiduddin A. The economic impact of Staphylococcus aureus infection in New York City Hospitals. Emerging Infectious Diseases. 1999. doi:10.3201/eid0501.990102

608. Austin DJ, Bonten MJ, Weinstein RA, Slaughter S, Anderson RM. Vancomycin-resistant enterococci in intensive-care hospital settings: transmission dynamics, persistence, and the impact of infection control programs. Proc Natl Acad Sci United States Am Sci. 1999; doi:10.1073/pnas.96.12.6908

609. Kirkland KB, Briggs JP, Trivette SL, Wilkinson WE, Sexton DJ. The Impact of Surgical-Site Infections in the 1990s: Attributable Mortality, Excess Length of Hospitalization, And Extra Costs. Infect Control &#x0026; Hosp Epidemiol. 2015/01/02. Cambridge University Press; 1999;20: 725–730. doi:DOI: 10.1086/501572

610. Carmeli Y, Eliopoulos GM, Samore MH. Antecedent Treatment with Different Antibiotic Agents as a Risk Factor for Vancomycin- Resistant Enterococcus. 2002;8: 802–807.

611. Pelz RK, Lipsett PA, Swoboda SM, Diener-West M, Powe NR, Brower RG, et al. Vancomycin-sensitive and vancomycin-resistant enterococcal infections in the ICU: Attributable costs and outcomes. Intensive Care Med. 2002; doi:10.1007/s00134-002-1276-8

612. Carmeli Y, Eliopoulos G, Mozaffari E, Samore M. Health and economic outcomes of vancomycin-resistant enterococci. Arch Intern Med. 2002; doi:10.1001/archinte.162.19.2223

613. Quach C, Weiss K, Moore D, Rubin E, McGeer A, Low DE. Clinical aspects and cost of invasive Streptococcus pneumoniae infections in children: Resistant vs. susceptible strains. Int J Antimicrob Agents. 2002; doi:10.1016/S0924-8579(02)00127-9

614. Engemann JJ, Carmeli Y, Cosgrove SE, Fowler VG, Bronstein MZ, Trivette SL, et al. Adverse Clinical and Economic Outcomes Attributable to Methicillin Resistance among Patients with Staphylococcus aureus Surgical Site Infection. Clin Infect Dis. 2003; doi:10.1086/367653

615. Eliopoulos GM, Cosgrove SE, Carmeli Y. The Impact of Antimicrobial Resistance on Health and Economic Outcomes. Clin Infect Dis. 2003; doi:10.1086/375081

616. Livermore DM. Bacterial Resistance: Origins, Epidemiology, and Impact. Clin Infect Dis. 2003; doi:10.1086/344654

617. Kaye KS, Engemann JJ, Mozaffari E, Carmeli Y. Reference group choice and antibiotic resistance outcomes. Emerg Infect Dis. 2004; doi:10.3201/eid1006.020665

618. Wilson SJ, Knipe CJ, Zieger MJ, Gabehart KM, Goodman JE, Volk HM, et al. Direct costs of multidrug-resistant Acinetobacter baumannii in the burn unit of a public teaching hospital. Am J Infect Control. 2004; doi:10.1016/j.ajic.2004.02.008

619. Wilson SJ, Knipe CJ, Zieger MJ, Gabehart KM. Direct costs of multidrug-resistant Acinetobacter baumannii in the burn unit of a public teaching hospital. 2004; 342–344. doi:10.1016/j.ajic.2004.02.008

620. Kopp BJ, Nix DE, Armstrong EP. Clinical and economic analysis of methicillin-susceptible and -resistant Staphylococcus aureus infections. Ann Pharmacother. 2004; doi:10.1345/aph.1E028 [1,1]

621. Weese JS. Methicillin-resistant Staphylococcus aureus in horses and horse personnel. Veterinary Clinics of North America - Equine Practice. 2004. doi:10.1016/j.cveq.2004.07.009

622. Tenover FC. Mechanisms of antimicrobial resistance in bacteria. Am J Infect Control. 2006; doi:10.1016/j.ajic.2006.05.219

623. Aloush V, Navon-Venezia S, Seigman-Igra Y, Cabili S, Carmeli Y. Multidrug-resistant Pseudomonas aeruginosa: Risk factors and clinical impact. Antimicrob Agents Chemother. 2006; doi:10.1128/AAC.50.1.43-48.2006

624. Kurth T, Walker AM, Glynn RJ, Chan KA, Gaziano JM, Berger K, et al. Results of multivariable logistic regression, propensity matching, propensity adjustment, and propensity-based weighting under conditions of nonuniform effect. Am J Epidemiol. 2006; doi:10.1093/aje/kwj047

625. Jackson D. The power of the standard test for the presence of heterogeneity in meta-analysis. Stat Med. John Wiley & Sons, Ltd; 2006;25: 2688–2699. doi:10.1002/sim.2481

626. Coia JE, Duckworth GJ, Edwards DI, Farrington M, Fry C, Humphreys H, et al. Guidelines for the control and prevention of meticillin-resistant Staphylococcus aureus (MRSA) in healthcare facilities. J Hosp Infect. 2006; doi:10.1016/j.jhin.2005.10.014

627. Shorr AF, Tabak YP, Gupta V, Johannes RS, Liu LZ, Kollef MH. Morbidity and cost burden of methicillin-resistant Staphylococcus aureus in early onset ventilator-associated pneumonia. Crit Care. 2006; doi:10.1186/cc4934

628. Schwarzer G. meta: An R package for meta-analysis. R News. 2007;7: 40–45.

629. Higgins CF. Multiple molecular mechanisms for multidrug resistance transporters. Nature. 2007. doi:10.1038/nature05630

630. Lodise TP, McKinnon PS. Burden of methicillin-resistant Staphylococcus aureus: Focus on clinical and economic outcomes. Pharmacotherapy. 2007. doi:10.1592/phco.27.7.1001

631. Alekshun MN, Levy SB. Molecular Mechanisms of Antibacterial Multidrug Resistance. Cell. 2007. doi:10.1016/j.cell.2007.03.004

632. Shah NS, Wright A, Bai GH, Barrera L, Boulahbal F, Martín-Casabona N, et al. Worldwide emergence of extensively drug-resistant tuberculosis. Emerg Infect Dis. 2007; doi:10.3201/eid1303.061400

633. Vandijck DM, Blot SI, Decruyenaere JM, Vanholder RC, De Waele JJ, Lameire NH, et al. Costs and length of stay associated with antimicrobial resistance in acute kidney injury patients with bloodstream infection. Acta Clin Belg. 2008; doi:10.1179/acb.2008.005

634. Goodman ER, Piatt R, Bass R, Onderdonk AB, Yokoe DS, Huang SS. Impact of an Environmental Cleaning Intervention on the Presence of Methicillin-Resistant Staphylococcus aureus and Vancomycin-Resistant Enterococci on Surfaces in Intensive Care Unit Rooms. Infect Control Hosp Epidemiol. 2008; doi:10.1086/588566

635. Depuydt PO, Vandijck DM, Bekaert MA, Decruyenaere JM, Blot SI, Vogelaers DP, et al. Determinants and impact of multidrug antibiotic resistance in pathogens causing ventilator-associated-pneumonia. Crit Care. 2008;12: R142. doi:10.1186/cc7119

636. Falagas ME, Karageorgopoulos DE. Pandrug Resistance (PDR), Extensive Drug Resistance (XDR), and Multidrug Resistance (MDR) among Gram-Negative Bacilli: Need for International Harmonization in Terminology. Clin Infect Dis. 2008;46: 1121–1122. Available: http://dx.doi.org/10.1086/528867

637. Gordon RJ, Lowy FD. Pathogenesis of Methicillin‐Resistant Staphylococcus aureus Infection. Clin Infect Dis. 2008; doi:10.1086/533591

638. Hidron AI, Edwards JR, Patel J, Horan TC, Sievert DM, Pollock DA, et al. Antimicrobial-Resistant Pathogens Associated With Healthcare-Associated Infections: Annual Summary of Data Reported to the National Healthcare Safety Network at the Centers for Disease Control and Prevention, 2006–2007. Infect Control Hosp Epidemiol. 2008; doi:10.1086/591861

639. Patel G, Huprikar S, Factor SH, Jenkins SG, Calfee DP. Outcomes of Carbapenem-Resistant Klebsiella pneumoniae Infection and the Impact of Antimicrobial and Adjunctive Therapies. Infect Control Hosp Epidemiol. 2008; doi:10.1086/592412

640. Depuydt PO, Vandijck DM, Bekaert MA, Decruyenaere JM, Blot SI, Vogelaers DP, et al. Determinants and impact of multidrug antibiotic resistance in pathogens causing ventilator-associated-pneumonia. 2008;12: 1–10. doi:10.1186/cc7119

641. Giske CG, Monnet DL, Cars O, Carmeli Y. Clinical and economic impact of common multidrug-resistant gram-negative bacilli. Antimicrobial Agents and Chemotherapy. 2008. doi:10.1128/AAC.01169-07

642. Peikes DN, Moreno L, Orzol SM. Propensity Score Matching. Am Stat. 2008; doi:10.1198/000313008X332016

643. Jain A, Dixit P. Multidrug resistant to extensively drug resistant tuberculosis: What is next? Journal of Biosciences. 2008. doi:10.1007/s12038-008-0078-8

644. Roberts RR, Hota B, Ahmad I, Scott R. Douglas II, Foster SD, Abbasi F, et al. Hospital and Societal Costs of Antimicrobial-Resistant Infections in a Chicago Teaching Hospital: Implications for Antibiotic Stewardship. Clin Infect Dis. 2009;49: 1175–1184. Available: http://dx.doi.org/10.1086/605630

645. Nikaido H. Multidrug Resistance in Bacteria. Annu Rev Biochem. 2009; doi:10.1146/annurev.biochem.78.082907.145923

646. Woodford N, Livermore DM. Infections caused by Gram-positive bacteria: a review of the global challenge. J Infect. 2009; doi:10.1016/S0163-4453(09)60003-7

647. Lode HM. Clinical impact of antibiotic-resistant Gram-positive pathogens. Clinical Microbiology and Infection. 2009. doi:10.1111/j.1469-0691.2009.02738.x

648. Resch A, Wilke M, Fink C. The cost of resistance: Incremental cost of methicillin-resistant Staphylococcus aureus (MRSA) in German hospitals. Eur J Heal Econ. 2009; doi:10.1007/s10198-008-0132-3

649. Nickerson EK, Hongsuwan M, Limmathurotsakul D, Wuthiekanun V, West TE, Teerawatanasuk N, et al. Staphylococcus aureus Bacteraemia in a Tropical Setting : Patient Outcome and Impact of Antibiotic Resistance. 2009;4. doi:10.1371/journal.pone.0004308

650. Zhang Y, Yew WW. Mechanisms of drug resistance in Mycobacterium tuberculosis. International Journal of Tuberculosis and Lung Disease. 2009. doi:10.1016/S0171-2985(11)80466-3

651. Lister PD, Wolter DJ, Hanson ND. Antibacterial-resistant Pseudomonas aeruginosa: Clinical impact and complex regulation of chromosomally encoded resistance mechanisms. Clinical Microbiology Reviews. 2009. doi:10.1128/CMR.00040-09

652. Stone PW. Economic burden of healthcare-associated infections: an American perspective. Expert Rev Pharmacoecon Outcomes Res. Taylor & Francis; 2009;9: 417–422. doi:10.1586/erp.09.53

653. Liberati A, Altman DG, Tetzlaff J, Mulrow C, Gøtzsche PC, Ioannidis JPA, et al. The PRISMA statement for reporting systematic reviews and meta-analyses of studies that evaluate health care interventions: explanation and elaboration. Journal of clinical epidemiology. 2009. doi:10.1016/j.jclinepi.2009.06.006

654. Roberts RR, Hota B, Ahmad I, Scott II RD, Foster SD, Abbasi F, et al. Hospital and Societal Costs of Antimicrobial‐Resistant Infections in a Chicago Teaching Hospital: Implications for Antibiotic Stewardship. Clin Infect Dis. 2009; doi:10.1086/605630

655. Hirsch EB, Tam VH. Impact of multidrug-resistant Pseudomonas aeruginosa infection on patient outcomes. Expert Review of Pharmacoeconomics and Outcomes Research. 2010. doi:10.1586/erp.10.49

656. Mauldin PD, Salgado CD, Hansen IS, Durup DT, Bosso JA. Attributable hospital cost and length of stay associated with health care-associated infections caused by antibiotic-resistant gram-negative bacteria. Antimicrob Agents Chemother. 2010; doi:10.1128/AAC.01041-09 [1,1]

657. Resistance to antifungal agents: Mechanisms and clinical impact. Chinese J Infect Chemother. 2010; doi:10.1086/524071

658. Parvizi J, Pawasarat IM, Azzam KA, Joshi A, Hansen EN, Bozic KJ. Periprosthetic joint infection: The economic impact of methicillin-resistant infections. J Arthroplasty. 2010; doi:10.1016/j.arth.2010.04.011

659. Otto M. Basis of Virulence in Community-Associated Methicillin-Resistant Staphylococcus aureus. Annu Rev Microbiol. 2010; doi:10.1146/annurev.micro.112408.134309

660. Gordon NC, Wareham DW. Multidrug-resistant Acinetobacter baumannii: mechanisms of virulence and resistance. International Journal of Antimicrobial Agents. 2010. doi:10.1016/j.ijantimicag.2009.10.024

661. Stang A. Critical evaluation of the Newcastle-Ottawa scale for the assessment of the quality of nonrandomized studies in meta-analyses. European Journal of Epidemiology. 2010. doi:10.1007/s10654-010-9491-z

*662. Mauldin PD, Salgado CD, Hansen IS, Durup DT, Bosso JA. Attributable Hospital Cost and Length of Stay Associated with Health Care-Associated Infections Caused by Antibiotic-Resistant Gram-Negative Bacteria ᰔ. 2010;54: 109–115. doi:10.1128/AAC.01041-09*

663. Blot S, Vandijck D, Lizy C, Annemans L, Vogelaers D. Estimating the Length of Hospitalization Attributable to Multidrug Antibiotic Resistance. 2010;54: 4046–4047. doi:10.1128/AAC.00689-10

664. Zaki SA, Karande S. Multidrug-resistant typhoid fever: a review. J Infect Dev Ctries. 2011; doi:10.3855/jidc.1405

665. Lye DC, Earnest A, Ling ML, Lee T, Yong H, Fisher DA, et al. The impact of multidrug resistance in healthcare-associated and nosocomial Gram-negative bacteraemia on mortality and length of stay : cohort study. European Society of Clinical Infectious Diseases; 2011; doi:10.1111/j.1469-0691.2011.03606.x

666. Stewardson AJ, Allignol A, Beyersmann J, Graves N, Schumacher M, Meyer R, et al. The health and economic burden of bloodstream infections caused by antimicrobial-susceptible and non-susceptible Enterobacteriaceae and Staphylococcus aureus in European hospitals , 2010 and 2011 : a multicentre retrospective cohort study. 2011;

667. de Kraker MEA, Wolkewitz M, Davey PG, Koller W, Berger J, Nagler J, et al. Burden of antimicrobial resistance in European hospitals: Excess mortality and length of hospital stay associated with bloodstream infections due to Escherichia coli resistant to third-generation cephalosporins. J Antimicrob Chemother. 2011; doi:10.1093/jac/dkq412

668. Kollef MH, Zilberberg MD, Shorr AF, Vo L, Schein J, Micek ST, et al. Epidemiology , microbiology and outcomes of bacteremia : A multicenter cohort study. J Infect. Elsevier Ltd; 2011;62: 130–135. doi:10.1016/j.jinf.2010.12.009

669. Kraker MEA De, Davey PG, Grundmann H. Mortality and Hospital Stay Associated with Resistant Staphylococcus aureus and Escherichia coli Bacteremia : Estimating the Burden of Antibiotic Resistance in Europe. 2011;8. doi:10.1371/journal.pmed.1001104

670. Horcajada JP, Shaw E, Padilla B, Pintado V, Calbo E, Benito N, et al. bacteraemic urinary tract infections in hospitalized patients : a prospective multicentre cohort study in the era of antimicrobial resistance. 2012; doi:10.1111/1469-0691.12089

671. Magiorakos AP, Srinivasan A, Carey RB, Carmeli Y, Falagas ME, Giske CG, et al. Multidrug-resistant, extensively drug-resistant and pandrug-resistant bacteria: An international expert proposal for interim standard definitions for acquired resistance. Clin Microbiol Infect. 2012; doi:10.1111/j.1469-0691.2011.03570.x

672. Wells GA et al. The Newcastle-Ottawa Scale (NOS) for assessing the quality if nonrandomized studies in meta-analyses. Evid based public Heal. 2012; doi:10.2307/632432

673. Karki S, Cheng AC. Impact of non-rinse skin cleansing with chlorhexidine gluconate on prevention of healthcare-associated infections and colonization with multi-resistant organisms: A systematic review. Journal of Hospital Infection. 2012. doi:10.1016/j.jhin.2012.07.005

674. Casali N, Nikolayevskyy V, Balabanova Y, Ignatyeva O, Kontsevaya I, Harris SR, et al. Microevolution of extensively drug-resistant tuberculosis in Russia. Genome Res. 2012; doi:10.1101/gr.128678.111

675. Neidell MJ, Cohen B, Furuya Y, Hill J, Jeon CY, Glied S, et al. Costs of healthcare-and community-associated infections with antimicrobial-resistant versus antimicrobial-susceptible organisms. Clin Infect Dis. 2012; doi:10.1093/cid/cis552

676. Kovalchik S. T u t o r i a l O n M e t a - A n a l y s i s I n R. 2013; 1–204.

677. Hartling L, Milne A, Hamm MP, Vandermeer B, Ansari M, Tsertsvadze A, et al. Testing the Newcastle Ottawa Scale showed low reliability between individual reviewers. J Clin Epidemiol. 2013; doi:10.1016/j.jclinepi.2013.03.003

678. Campoccia D, Montanaro L, Arciola CR. A review of the biomaterials technologies for infection-resistant surfaces. Biomaterials. 2013. doi:10.1016/j.biomaterials.2013.07.089

679. Stewardson A, Fankhauser C, Augelis G De, Rohner P, Safran E, Schrenzel J, et al. Burden of Bloodstream Infection Caused by Extended-Spectrum β-Lactamase–Producing Enterobacteriaceae Determined Using Multistate Modeling at a Swiss University Hospital and a Nationwide Predictive Model. Infect Control Hosp Epidemiol. 2013; doi:10.1086/669086

680. Tadros M, Williams V, Coleman BL, Mcgeer AJ, Haider S, Iacovides H, et al. Epidemiology and Outcome of Pneumonia Caused by Methicillin-Resistant Staphylococcus aureus ( MRSA ) in Canadian Hospitals. 2013;8: 4–11. doi:10.1371/journal.pone.0075171

681. Uhlemann AC, Otto M, Lowy FD, DeLeo FR. Evolution of community- and healthcare-associated methicillin-resistant Staphylococcus aureus. Infect Genet Evol. 2014; doi:10.1016/j.meegid.2013.04.030

682. Stygall J, Newman S. Hospital acquired infection. Cambridge Handbook of Psychology, Health and Medicine, Second Edition. 2014. doi:10.1017/CBO9780511543579.182

683. Matteelli A, Roggi A, Carvalho ACC. Extensively drug-resistant tuberculosis: Epidemiology and management. Clinical Epidemiology. 2014. doi:10.2147/CLEP.S35839

684. Perez KK, Olsen RJ, Musick WL, Cernoch PL, Davis JR, Peterson LE, et al. Integrating rapid diagnostics and antimicrobial stewardship improves outcomes in patients with antibiotic-resistant Gram-negative bacteremia. J Infect. 2014; doi:10.1016/j.jinf.2014.05.005

685. Tedja R, Nowacki A, Fraser T, Fatica C, Griffiths L, Gordon S, et al. The impact of multidrug resistance on outcomes in ventilator-associated pneumonia. Am J Infect Control. 2014;42: 542–545. doi:https://doi.org/10.1016/j.ajic.2013.12.009

686. Lalloo UG, Pillay S, Mngqibisa R, Ambaram A. Update on extensively drug-resistant tuberculosis. Clin Pulm Med. 2014; doi:10.1097/CPM.0000000000000058

687. Nathwani D, Raman G, Sulham K, Gavaghan M, Menon V. Clinical and economic consequences of hospital-acquired resistant and multidrug-resistant Pseudomonas aeruginosa infections: A systematic review and meta-analysis. Antimicrob Resist Infect Control. 2014; doi:10.1186/2047-2994-3-32

688. Tedja R, Nowacki A, Fraser T, Fatica C, Grif L, Gordon S, et al. American Journal of Infection Control The impact of multidrug resistance on outcomes in ventilator-associated pneumonia. 2014;42: 542–545. doi:10.1016/j.ajic.2013.12.009

689. Bartoletti M, Giannella M, Caraceni P, Domenicali M, Ambretti S, Tedeschi S, et al. Epidemiology and outcomes of bloodstream infection in patients with cirrhosis. European Association for the Study of the Liver; 2014;61: 51–58. doi:10.1016/j.jhep.2014.03.021

690. Blair JMA, Webber MA, Baylay AJ, Ogbolu DO, Piddock LJ V. Molecular mechanisms of antibiotic resistance. Nature Reviews Microbiology. 2015. doi:10.1038/nrmicro3380

691. Gulen TA, Guner R, Celikbilek N, Keske S, Tasyaran M. Clinical importance and cost of bacteremia caused by nosocomial multi drug resistant acinetobacter baumannii. Int J Infect Dis. International Society for Infectious Diseases; 2015;38: 32–35. doi:10.1016/j.ijid.2015.06.014

692. Martin-Loeches I, Torres A, Rinaudo M, Terraneo S, de Rosa F, Ramirez P, et al. Resistance patterns and outcomes in intensive care unit (ICU)-acquired pneumonia. Validation of European Centre for Disease Prevention and Control (ECDC) and the Centers for Disease Control and Prevention (CDC) classification of multidrug resistant organi. J Infect. 2015;70: 213–222. doi:https://doi.org/10.1016/j.jinf.2014.10.004

693. Micek ST, Wunderink RG, Kollef MH, Chen C, Rello J, Chastre J, et al. An international multicenter retrospective study of Pseudomonas aeruginosa nosocomial pneumonia: Impact of multidrug resistance. Crit Care. 2015; doi:10.1186/s13054-015-0926-5

694. Gulen TA, Guner R, Celikbilek N, Keske S, Tasyaran M. Clinical importance and cost of bacteremia caused by nosocomial multi drug resistant acinetobacter baumannii. Int J Infect Dis. 2015; doi:10.1016/j.ijid.2015.06.014

695. Kopp BJ, Nix DE, Armstrong EP. and -Resistant Staphylococcus aureus Infections. 2015; doi:10.1345/aph.1E028

696. Martin-loeches I, Torres A, Rinaudo M, Terraneo S, Rosa F De, Ramirez P, et al. Resistance patterns and outcomes in intensive care unit ( ICU ) -acquired pneumonia . Validation of European Centre for Disease Prevention and Control ( ECDC ) and the Centers for Disease Control and Prevention ( CDC ) classification of multidrug resistan. 2015; doi:10.1016/j.jinf.2014.10.004

697. Riu M, Chiarello P, Terradas R, Sala M, Garcia-Alzorriz E, Castells X, et al. Cost attributable to nosocomial bacteremia. Analysis according to microorganism and antimicrobial sensitivity in a university hospital in Barcelona. PLoS One. 2016; doi:10.1371/journal.pone.0153076

698. Friedman ND, Temkin E, Carmeli Y. The negative impact of antibiotic resistance. Clinical Microbiology and Infection. 2016. doi:10.1016/j.cmi.2015.12.002

699. Jasovský D, Littmann J, Zorzet A, Cars O. Antimicrobial resistance-a threat to the world’s sustainable development. Ups J Med Sci. 2016/07/13. Taylor & Francis; 2016;121: 159–164. doi:10.1080/03009734.2016.1195900

700. Baym M, Stone LK, Kishony R. Multidrug evolutionary strategies to reverse antibiotic resistance. Science. 2016. doi:10.1126/science.aad3292

701. Nelson RE, Schweizer ML, Perencevich EN, Nelson SD, Khader K, Chiang H, et al. Costs and Mortality Associated With Multidrug-Resistant Healthcare-Associated Acinetobacter Infections. 2016;37. doi:10.1017/ice.2016.145

702. Uematsu H, Yamashita K, Kunisawa S, Fushimi K, Imanaka Y. The economic burden of methicillin-resistant Staphylococcus aureus in community-onset pneumonia inpatients. Am J Infect Control. 2016; doi:10.1016/j.ajic.2016.05.008

703. Department of Health. Antimicrobial Resistance Empirical and Statistical Evidence-Base. Public Heal Engl. 2016;

704. Hoaglin DC. Misunderstandings about Q and ‘Cochran’s Q test’’ in meta-analysis.’ Stat Med. John Wiley & Sons, Ltd; 2016;35: 485–495. doi:10.1002/sim.6632

705. Resch A, Wilke ÆM, Fink ÆC. The cost of resistance : Incremental cost of methicillin-resistant Staphylococcus aureus ( MRSA ) in German hospitals The cost of resistance : incremental cost of methicillin-resistant Staphylococcus aureus ( MRSA ) in German hospitals. 2016; doi:10.1007/s10198-008-0132-3

706. Suwantarat N, Carroll KC. Epidemiology and molecular characterization of multidrug-resistant Gram-negative bacteria in Southeast Asia. Antimicrob Resist Infect Control. 2016; doi:10.1186/s13756-016-0115-6

707. Huebner C, Roggelin M, Flessa S. Economic burden of multidrug-resistant bacteria in nursing homes in Germany : a cost analysis based on empirical data. 2016; 1–6. doi:10.1136/bmjopen-2015-008458

708. O-Neill J. Tackling drug-resistant infections globally: final report and recommendations. 2016.

709. Thaden JT, Park LP, Maskarinec SA, Ruffin F, Fowler VG, Duin V. crossm Cohort Study Show Increased Mortality Caused by Pseudomonas aeruginosa Compared to Other Bacteria. 2017;61: 1–11.

710. Borgatta B, Gattarello S, Mazo CA, Imbiscuso AT, Larrosa MN. The clinical significance of pneumonia in patients with respiratory specimens harbouring multidrug-resistant Pseudomonas aeruginosa : a 5-year retrospective study following 5667 patients in four general ICUs. European Journal of Clinical Microbiology & Infectious Diseases; 2017; 2155–2163. doi:10.1007/s10096-017-3039-z

711. Abat C, Rolain J, Dubourg G, Fournier P, Chaudet H, Raoult D. Evaluating the Clinical Burden and Mortality Attributable to Antibiotic Resistance : The Disparity of Empirical Data and Simple Model Estimations. 2017;65. doi:10.1093/cid/cix346

712. Fernández-Barat L, Ferrer M, De Rosa F, Gabarrús A, Esperatti M, Terraneo S, et al. Intensive care unit-acquired pneumonia due to Pseudomonas aeruginosa with and without multidrug resistance. J Infect. 2017;74: 142–152. doi:https://doi.org/10.1016/j.jinf.2016.11.008

713. Dicks K V., Anderson DJ, Baker AW, Sexton DJ, Lewis SS. Clinical Outcomes and Healthcare Utilization Related to Multidrug-Resistant Gram-Negative Infections in Community Hospitals. Infect Control Hosp Epidemiol. 2017; doi:10.1017/ice.2016.230

714. Thaden JT, Li Y, Ruffin F, Maskarinec SA, Hill-rorie JM, Wanda LC, et al. crossm Increased Costs Associated with Bacteria Are Due Primarily to Patients with Hospital-Acquired Infections. 2017;61: 1–10.

715. Uematsu H, Yamashita K, Kunisawa S, Fushimi K, Imanaka Y. Estimating the disease burden of methicillin- resistant Staphylococcus aureus in Japan : Retrospective database study of Japanese hospitals. 2017; 1–12. doi:10.1371/journal.pone.0179767

716. Thaden JT, Li Y, Ruffin F, Maskarinec SA, Hill-Rorie JM, Wanda LC, et al. Increased costs associated with bloodstream infections caused by multidrug-resistant gram-negative bacteria are due primarily to patients with hospital-acquired infections. Antimicrob Agents Chemother. 2017; doi:10.1128/AAC.01709-16

717. Nelson RE, Slayton RB, Stevens VW, Jones MM, Khader K, Rubin MA, et al. Attributable Mortality of Healthcare-Associated Infections Due to Multidrug-Resistant Gram-Negative Bacteria and Methicillin-Resistant Staphylococcus Aureus. 2017;38. doi:10.1017/ice.2017.83

718. Brady M, Oza A, Cunney R, Burns K. Attributable mortality of hospital-acquired bloodstream infections in Ireland. J Hosp Infect. Elsevier Ltd; 2017;96: 35–41. doi:10.1016/j.jhin.2017.02.006

719. Baker AW, Dicks K V, Durkin MJ. HHS Public Access. 2017;37: 519–526. doi:10.1017/ice.2016.13.Epidemiology

720. Fern L, Rosa F De, Gabarr A. Intensive care unit-acquired pneumonia due to Pseudomonas aeruginosa with and without multidrug resistance *. 2017; 142–152. doi:10.1016/j.jinf.2016.11.008

721. Roumbelaki M, Ioannidou E. Prevalence , incidence burden , and clinical impact of healthcare-associated infections and antimicrobial resistance : a national prevalent cohort study in acute care hospitals in Greece. 2017;

722. Adrie C, Ibn W, Schwebel C, Darmon M, Mourvillier B, Dumenil A, et al. Attributable mortality of ICU-acquired bloodstream infections : Impact of the source , causative micro-organism , resistance profile and antimicrobial therapy. 2017; doi:10.1016/j.jinf.2016.11.001

723. Europe. W. Fact sheets on sustainable development goals: health targets. 2017.

724. Engler-Hüsch S, Heister T, Mutters NT, Wolff J, Kaier K. In-hospital costs of community-acquired colonization with multidrug-resistant organisms at a German teaching hospital. BMC Health Serv Res. 2018; doi:10.1186/s12913-018-3549-0

725. Zhen X, Li Y, Chen Y, Dong P, Liu S, Dong H. Effect of multiple drug resistance on total medical costs among patients with intra- abdominal infections in China. 2018; 1–12.

726. Mueller M, D’Addario M, Egger M, Cevallos M, Dekkers O, Mugglin C, et al. Methods to systematically review and meta-analyse observational studies: a systematic scoping review of recommendations. BMC Med Res Methodol. 2018;18: 44. doi:10.1186/s12874-018-0495-9

727. Magira EE, Islam S, Niederman MS. Multi-drug resistant organism infections in a medical ICU : Association to clinical features and impact upon. Med Intensiva (English Ed. Elsevier Espa&ntilde;a, S.L.U. and SEMICYUC; 2018;42: 225–234. doi:10.1016/j.medine.2017.07.003

728. Publishing O. Stemming the Superbug Tide: Just A Few Dollars More. 2018.

729. Pouwels KB, Vansteelandt S, Batra R, Edgeworth JD. Intensive care unit ( ICU ) -acquired bacteraemia and ICU mortality and discharge : addressing time-varying confounding using appropriate methodology. J Hosp Infect. Elsevier Ltd; 2018;99: 42–47. doi:10.1016/j.jhin.2017.11.011

730. Cassini A, Högberg LD, Plachouras D, Quattrocchi A, Hoxha A, Simonsen GS, et al. Attributable deaths and disability-adjusted life-years caused by infections with antibiotic-resistant bacteria in the EU and the European Economic Area in 2015: a population-level modelling analysis. Lancet Infect Dis. 2019;19: 56–66. doi:https://doi.org/10.1016/S1473-3099(18)30605-4

731. Investigation O. Health and Economic Outcomes of Antibiotic Resistance in. 2019;159: 1127–1132.

732. Chen Z, Xu Z, Wu H, Chen L, Gao S, Chen Y. The impact of carbapenem-resistant Pseudomonas aeruginosa on clinical and economic outcomes in a Chinese tertiary care hospital : A propensity score − matched analysis. AJIC Am J Infect Control. Elsevier Inc.; 2019;47: 677–682. doi:10.1016/j.ajic.2018.10.025

733. Jia H, Li W, Hou T, Ma H, Yang Y, Wu A, et al. The Attributable Direct Medical Cost of Healthcare Associated Infection Caused by Multidrug Resistance Organisms in 68 Hospitals of China. 2019;2019.
