# Supplementary material for: Impact of multi-drug resistant bacteria on economic and clinical outcomes of healthcare-associated infections in adults: Systematic review and meta-analysis
Source: PLoS One. 2020 Jan 10;15(1):e0227139. doi: 10.1371/journal.pone.0227139 (PMC6953842; doi:10.1371/journal.pone.0227139)
Supplement: S1 Table — (DOCX) [file pone.0227139.s005.docx]

**Table S1.** Summary of Newcastle-Ottawa risk of bias judgements for each study.

| Study | Year | Selection | Comparability | Exposure | Total | AHQR Class |
| --- | --- | --- | --- | --- | --- | --- |
| R. K. Pelz et al. | 2002 | 3* | 1* | 2* | 6* | Good |
| L. F. Barat et al. | 2017 | 2* | 2* | 2* | 6* | Fair |
| B. J. Kopp et al. | 2004 | 4* | 1* | 0* | 6* | Poor |
| R. Tedja et al. | 2014 | 3* | 2* | 3* | 8* | Good |
| P. O. Depuydt | 2008 | 3* | 1* | 2* | 6* | Good |
| I. M. Loeches et al. | 2014 | 2* | 1* | 2* | 5* | Fair |
| P. D. Mauldin et al. | 2010 | 3* | 1* | 1* | 5* | Poor |
| J. J. Engemann et al. | 2003 | 2* | 2* | 2* | 6* | Fair |
| E. E. Magira et al. | 2017 | 3* | 1* | 3* | 7* | Good |
| Y. Carmeli et al. | 1999 | 3* | 2* | 2* | 7* | Good |
| M. Riu et al. | 2016 | 2* | 1* | 1* | 4* | Poor |
| R. R. Roberts et al. | 2009 | 4* | 1* | 2* | 7* | Good |
| S.T. Micek et al. | 2015 | 3* | 1* | 2* | 6* | Good |
| Z. Chen et al. | 2018 | 3* | 2* | 2* | 7* | Good |
| A. Resch et al. | 2009 | 2* | 1* | 3* | 6* | Fair |
| M. J. Neidell et al. | 2012 | 3* | 1* | 1* | 5* | Poor |
| L. Puchter et al. | 2018 | 3* | 1* | 2* | 6* | Fair |
| R. Nelson et al. | 2018 | 3* | 1* | 2* | 6* | Fair |
| E. Cowie et al. | 2005 | 2* | 1* | 1* | 4* | Poor |
| Bonnet et al. | 2019 | 4* | 2* | 2* | 8* | Good |

AHQR Class: Agency for Healthcare Research and Quality standards.
