# Supplementary material for: Impact of multi-drug resistant bacteria on economic and clinical outcomes of healthcare-associated infections in adults: Systematic review and meta-analysis
Source: PLoS One. 2020 Jan 10;15(1):e0227139. doi: 10.1371/journal.pone.0227139 (PMC6953842; doi:10.1371/journal.pone.0227139)
Supplement: S2 Table — (DOCX) [file pone.0227139.s006.docx]

**Table S2.** Quantitative summary of included studies

| Id | Authors | Year | Pathogens | Case Definition (MDR) | N Cases | N Controls | Control method |
| --- | --- | --- | --- | --- | --- | --- | --- |
| 1 | R. K. Pelz et al. | 2002 | EF | VR | 12 | 22 | LRM |
| 2 | L. F. Barat et al. | 2017 | PA | ECDC/CDC | 22 | 42 | LRM |
| 3 | B. J. Kopp et al. | 2004 | SA | MR | 36 | 36 | PS |
| 4 | R. Tedja et al. | 2014 | SA; PA; KP; AB | ECDC/CDC | 49 | 58 | LRM |
| 5 | P. O. Depuydt | 2008 | SA; EF; PA | MR; VR; LER | 52 | 140 | LRM |
| 6 | I. M. Loeches et al. | 2014 | SA; EF; PA | ECDC/CDC | 89 | 82 | LRM |
| 7 | P. D. Mauldin et al. | 2010 | Gram-Negative | Any | 103 | 559 | LRM |
| 8 | J. J. Engemann et al. | 2003 | SA | MR | 121 | 165 | LRM |
| 9 | E. E. Magira et al. | 2017 | AB; EC; PA; KP; EF; SA | ECDC/CDC | 127 | 177 | LRM |
| 10 | Y. Carmeli et al. | 1999 | PA | Any | 144 | 345 | LRM |
| 11 | M. Riu et al. | 2016 | EC; KP; PA; SA | ECDC/CDC | 167 | 404 | PS |
| 12 | R. R. Roberts et al. | 2009 | SA; EF; EC; PA | MR; VR; C3R; IMR | 169 | 169 | PS & LRM |
| 13 | S.T. Micek et al. | 2015 | PA | ECDC/CDC | 226 | 514 | LRM |
| 14 | Z. Chen et al. | 2018 | PA | C3R | 270 | 270 | PS |
| 15 | A. Resch et al. | 2009 | SA | MR | 1,026 | 1,026 | PS |
| 16 | M. J. Neidell et al. | 2012 | AB; EF; KP; PA; SA | OXR; VR; IMR; LER; AR | 1,083 | 692 | LRM |
| 17 | L. Puchter et al. | 2018 | EF | VR/CDC | 42 | 42 | MM |
| 18 | R. Nelson et al. | 2018 | AB; PA; EF | NLP | 149 | 256 | LRM |
| 19 | E. Cowie et al. | 2005 | SA | CDC | 22 | 15 | LRM |
| 20 | Bonnet et al. | 2019 | All | EUCAST | 2,213 | 3,312 | LRM |

Abbreviations: MDR: Multidrug resistance; EF: Enterococci; PA: Pseudomonas Aeruginosa; SA: Staphylococcus aureus; KP: Klebsiella pneumoniae; AB: Acinetobacter baumannii; EC: Escherichia coli; VR: Vancomycin-resistant; ECDC/CDC: interim definition by the European Center for Disease Control and Center for Disease Control; LER: Levofloxacin-resistant; C3R: 3^rd^ generation cephalosporin resistance; IMR: Imipenem/meropenem-resistant; OXR: Oxacilin-resistant; AR: ampicillin-resistant; LRM: multivariate linear/logistic regression; PS: Propensity score matching; MM: Manual matching; NLP: Natural language processing.
