# Supplementary material for: Impact of multi-drug resistant bacteria on economic and clinical outcomes of healthcare-associated infections in adults: Systematic review and meta-analysis
Source: PLoS One. 2020 Jan 10;15(1):e0227139. doi: 10.1371/journal.pone.0227139 (PMC6953842; doi:10.1371/journal.pone.0227139)
Supplement: S3 Table — (DOCX) [file pone.0227139.s007.docx]

**Table S3.** Search strategy by database

| Database/Search Engine | Search Strategy | Results |
| --- | --- | --- |
| Pubmed | ("antimicrobial resistance"[MeSH Major Topic] OR “antibiotic stewardship”[All Fields] OR "resistance"[All Fields] OR "MDR"[All Fields] OR "VRE"[All Fields] OR "MRSA"[All Fields] OR "CRE"[All Fields] OR "PDR"[All Fields] OR "multidrug-resistant"[All Fields] OR "antibiotic resistance"[All Fields]) AND ("Acquired Infection"[All Fields] OR "HAI"[All Fields] OR "Nosocomial Infection"[All Fields]) AND ("Mortality"[All Fields] OR "Length of Stay"[All Fields] OR "Cost"[All Fields] OR "Outcome"[All Fields] OR "clinical impact"[All Fields] OR "impact"[All Fields]) AND ("case control"[All Fields] OR "Cohort"[All Fields] OR "control"[All Fields] OR “matching”[MeSH]) | 629 |
| Google Scholar | “Antimicrobial resistance” "outcome" "nosocomial" "hospital acquired" "healthcare acquired" | 510 |
